# Synthesis and Structure–Activity Relationships of Aristoyagonine Derivatives as Brd4 Bromodomain Inhibitors with X-ray Co-Crystal Research

**DOI:** 10.3390/molecules26061686

**Published:** 2021-03-17

**Authors:** Minjin Yoo, Tae Hyun Park, Miyoun Yoo, Yeongrin Kim, Joo-Youn Lee, Kyu Myung Lee, Seong Eon Ryu, Byung Il Lee, Kwan-Young Jung, Chi Hoon Park

**Affiliations:** 1 Department of Medicinal Chemistry and Pharmacology, University of Science & Technology, Daejeon 34113, Korea; yminjin10@gmail.com (M.Y.); kyr@krict.re.kr (Y.K.); 2 Research Institute, National Cancer Center, Goyang-si, Gyeonggi 10408, Korea; 74977@ncc.re.kr; 3 Department of Bioengineering, Hanyang University, Seoul 04763, Korea; ryuse@hanyang.ac.kr; 4 Therapeutics & Biotechnology Division, Korea Research Institute of Chemical Technology, Daejeon 34114, Korea; miyounyoo@gmail.com (M.Y.); leejy@krict.re.kr (J.-Y.L.); kmlee@krict.re.kr (K.M.L.); 5 Department of Cancer Biomedical Science, National Cancer Center Graduate School of Cancer Science and Policy, Goyang-si, Gyeonggi 10408, Korea

**Keywords:** Brd4, aristoyagonine, epigenetic, crystal structure, SAR

## Abstract

Epigenetic regulation is known to play a key role in progression of anti-cancer therapeutics. Lysine acetylation is an important mechanism in controlling gene expression. There has been increasing interest in bromodomain owing to its ability to modulate transcription of various genes as an epigenetic ‘reader.’ Herein, we report the design, synthesis, and X-ray studies of novel aristoyagonine (benzo[6,7]oxepino[4,3,2-*cd*]isoindol-2(1*H*)-one) derivatives and investigate their inhibitory effect against Brd4 bromodomain. Five compounds **8ab**, **8bc**, **8bd**, **8be**, and **8bf** have been discovered with high binding affinity over the Brd4 protein. Co-crystal structures of these five inhibitors with human Brd4 bromodomain demonstrated that it has a key binding mode occupying the hydrophobic pocket, which is known to be the acetylated lysine binding site. These novel Brd4 bromodomain inhibitors demonstrated impressive inhibitory activity and mode of action for the treatment of cancer diseases.

## 1. Introduction

Gene transcription is regulated by multiple steps, including epigenetic modification. Epigenetic regulation is the dynamic and reversible modification of histones and DNA [1]. Among the various types of epigenetic modification [2], lysine acetylation plays a significant role in chromatin structure regulation [3]. Aberrant lysine acetylation is known to cause cancer via dysregulation of gene expression [4]. Most of the histone modifications are performed in three distinct steps: adding; removing; and reading [5,6]. The proteins involved in these steps are well studied in acetyl modification of lysine. Histone acetyltransferase (HAT) adds acetyl groups, while histone deacetylase removes acetyl groups; bromodomain reads the acetyl group [7].

Bromodomain, a conserved 110 amino acid module, is widely adapted in more than 50 cellular proteins [8]. These proteins exist in the nucleus or cytoplasm and have diverse functions and structures; they include chromatin-modifying proteins, helicases, chromatin remodelers, and transcriptional co-activators. There are several bromodomain subfamilies; Brd2, Brd3, Brd4, and the testis-specific protein Brdt belong to the bromodomain and extra-terminal (BET) family [9]. BET proteins have a conserved modular structure including a pair of bromodomain. It preferentially binds to ε-*N*-acetylated lysine residue of histone through its binding pocket [10]. Subsequently, transcription and chromatin remodeling proteins, including HATs, histone methyltransferases (HMTs), transcription initiation factors, and ATP-dependent helicases, are recruited by Brd4 to chromatin [11]. Brd4 acts as an important mediator of transcriptional elongation through recruiting the positive transcription elongation factor complex (P-TEFb) [12,13].

**Figure 1:**
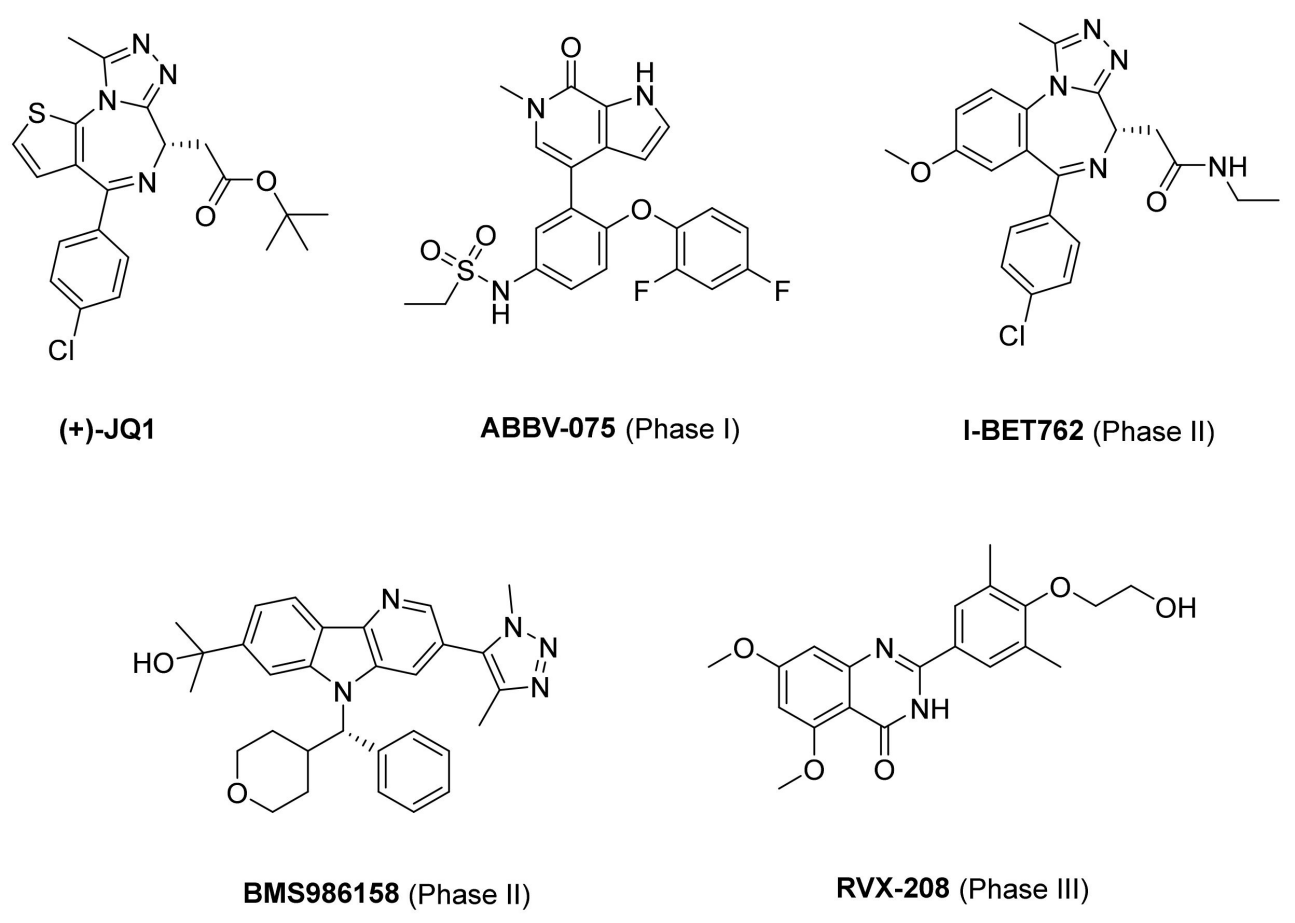
Chemical structures of representative BET bromodomain inhibitors.

Brd4 is fused with NUclear protein in Testis (NUT) in most of the patients with NUT midline carcinoma (NMC) [14,15]. As Brd4-nut knockdown significantly suppresses the proliferation of NMC cell line, Brd4-nut gene translocation is believed to be the driving force for NMC initiation [16]. Besides NMC, Zuber et al. found that Brd4 is a potent target for acute myeloid leukemia through RNAi screen [17]. They reported that JQ-1, which abrogates the interaction between Brd4 bromodomain and histone peptide with acetylated lysine, suppressed AML proliferation by considerably downregulating c-Myc protein level. Subsequent studies found that JQ-1 is an effective anti-cancer agent in most leukemic tumors [18]. Many novel bromodomain inhibitors have been developed in previous studies (Figure 1) [19,20,21,22].

In our previous study, we reported that aristoyagonine efficiently inhibited the binding of Brd4 bromodomain to acetylated histone peptide [23]. This was the first study to report effective natural product derivatives against bromodomain. In the present study, we aimed to synthesize aristoyagonine derivatives (benzo[6,7]oxepino[4,3,2-*cd*]isoindol-2(1*H*)-one compounds) and determine their enzymatic and cellular activities. In addition, we aimed to elucidate the crystal structures of bromodomain in complex with aristoyagonine derivatives and identify the inhibitor binding modes.

## 2. Results and Discussion

### 2.1. Design

**Figure 2:**
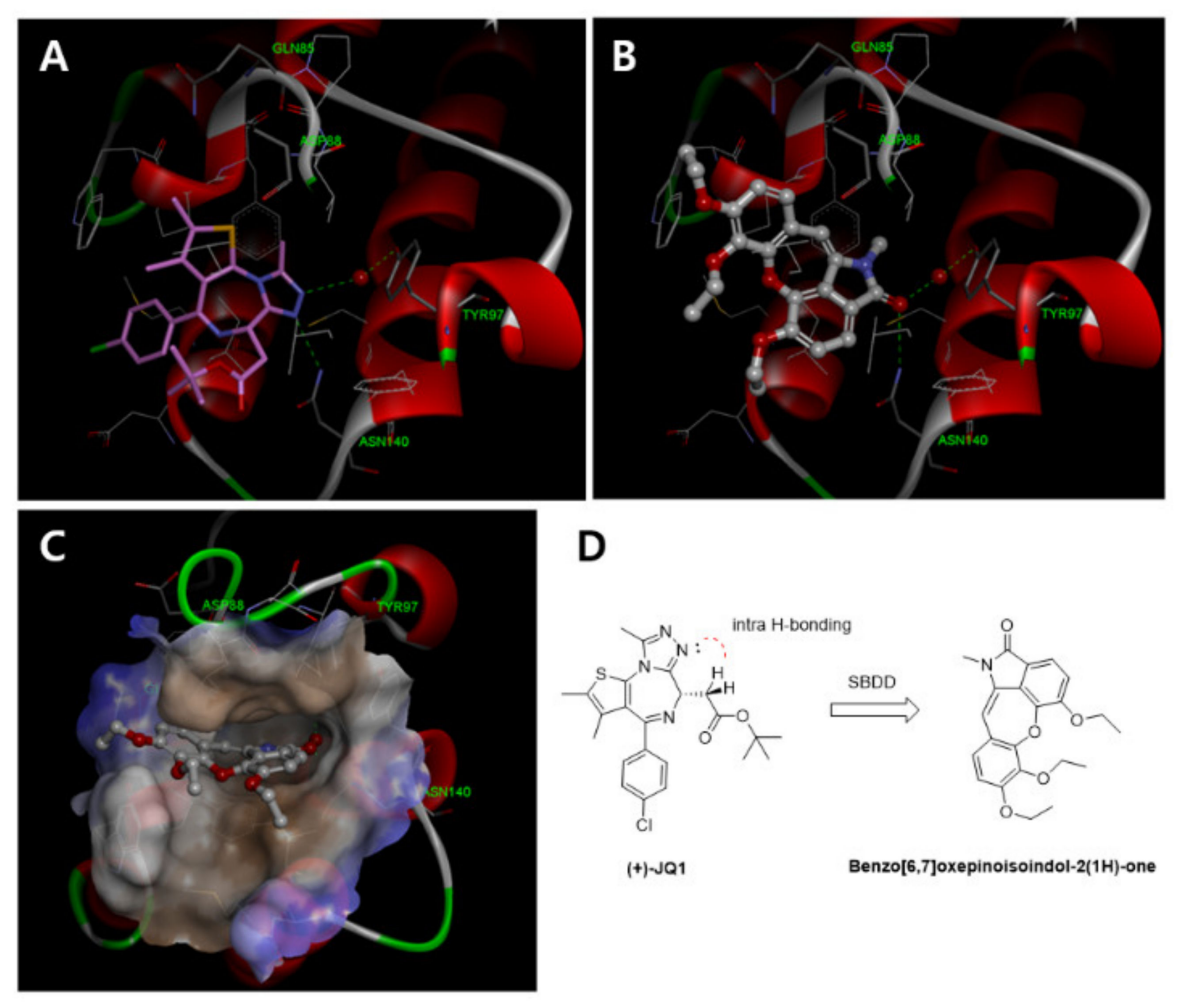
Binding pocket of Brd4 bromodomain and ligand structure-based drug design (SBDD). (**A**) Crystal structure of the bromodomain of human Brd4 in complex with the inhibitor JQ-1(PDB code: 3MXF, human 1.6 Å). (**B**) Molecular docking of designed compound of aristoyanine with Brd4 bromodomain binding pocket. (**C**) Designed docking model with Gaussian surface. (**D**) Novel compound benzo[6,7]oxepinoisoindol-2(1*H*)one scaffold derived from JQ-1.

We previously reported that aristoyagonine exhibited Brd4 bromodomain inhibitory activity [23]. Based on the X-ray co-crystal structure (PBD code: 3MXF, human 1.6 Å) of JQ-1-bromodomain, we performed a docking study to reveal the binding mode between bromodomain and aristoyagonine. X-ray co-crystal structure showed two hydrogen bonds between bromodomain and JQ-1. The nitrogen atom of triazolodiazepine binds to the amino acid residue Asn140 via hydrogen bonding, and Tyr97 forms a hydrogen bond to the nitrogen atom of triazolodiazepine through a water molecule. The methyl group of triazole ring of JQ-1 shows a hydrophobic interaction with Val87 and Phe83; the dimethylthiophen group can be stabilized by hydrophobic interaction with Pro82 and Leu92. Molecular analysis revealed that a binding pocket was generated around the key functional site between bromodomain and inhibitor JQ-1; then, aristoyagonine was applied to the docking model. The docking model revealed that the amino acid residue Asn140 forms a direct hydrogen bond with the carbonyl oxygen of the isoindolinone moiety of aristoyagonine; it forms another hydrogen bond with Tyr97 via a water molecule. It was confirmed that the methyl group of indole is inside a hydrophobic pocket composed of Val87 and Phe83, and the fused ring forms a hydrophobic interaction with Pro82 and Leu92. Structure-based drug design in JQ-1 predicted an intra-hydrogen bonding between the nitrogen atom present in 1,3,4-triazole and hydrogen atom of diazepine-CH_2_ (Figure 2D). Therefore, JQ-1 is considered to bind the bromodomain in a similar manner as that to a four fused ring compound. Therefore, we hypothesized that benzo[6,7]oxepinoisoindole-2(1*H*)one, the skeleton of aristoyagonine, could be developed as a novel Brd4 inhibitor. 

### 2.2. Chemistry

**Scheme 1:**
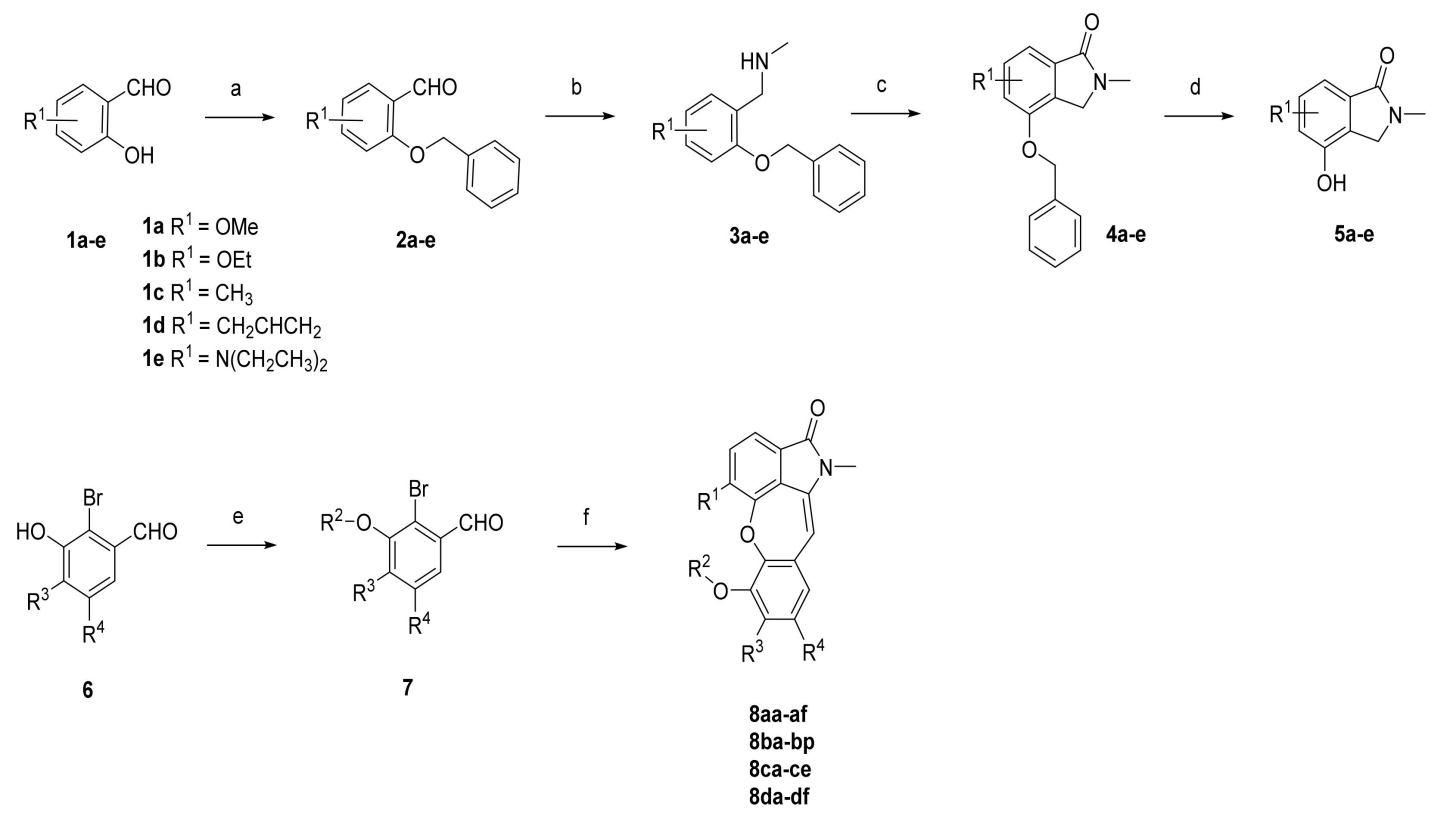
Synthesis procedure of benzo[6,7]oxepino[4,3,2-*cd*]isoindol-2(1*H*)-one derivatives [24]. Reaction scheme. Reagents and conditions: (a) benzyl bromide, K_2_CO_3_, Acetone, 70 °C, 9 h; (b) (i) 40% MeNH_2_ in MeOH, 48 h; (ii) NaBH_4_, MeOH, 0 °C, 18 h; (c) CO(g), Cu(OAc)_2_, Pd(OAc)_2_, Toluene, O_2_(g), 130 °C, 18 h; (d) 10 wt % Pd/C, H_2_, MeOH/EtOAc = 1:1 ratio, r.t., 12 h; (e) R^2^—X, K_2_CO_3_, DMF, r.t., 12 h; (f) **5a**–**e**, CuBr, Cs_2_CO_3_, 4Å molecular sieves, pyridine, 100 °C, 24 h.

Based on molecular modeling, it was found that Asn140, an important amino acid residue of Brd4 bromodomain, forms a strong hydrogen bond with the carbonyl oxygen of compounds. We fixed the carbonyl group on the synthesized compounds to maintain a strong hydrogen bonding between Brd4 bromodomain and compounds. To synthesize the designed compounds containing benzo[6,7]oxepinoisoindol-2(1*H*)one scaffold, we followed the one-pot Cu-catalyzed etherification/aldol condensation cascade reaction [24]. Subunits were synthesized as described in Scheme 1. With salicylaldehyde derivatives (**1a**–**e**) as starting compounds, nucleophilic substitution reactions with benzyl bromide in the presence of excess K_2_CO_3_ yielded *O*-benzyl protected derivatives (**2a**–**e**). Treatment of methylamine in the presence of anhydrous methanol solvent resulted in reductive amination, while reduction by NaBH_4_ yielded the desired compounds (**3a**–**e**). Ring cyclization using CO gas in the presence of Cu(OAc)_2_ and a catalytic amount of Pd(OAc)_2_ afforded 2-methylisoindolin-1-one backbone (**4a**–**e**). Debenzylation of *O*-benzyl-2-methylisoindolin-2-one performed using Pd/C in H_2_ atmosphere afforded the top subunits (**5a**–**e**). The bottom subunits were synthesized from substituted 2-bromo-3-hydroxybenzaldehyde (**6**) as the starting compound with several alkyl/aryl halides to yield the desired compound (**7**). To obtain the final compounds, the top subunits **5a**–**e** were treated by Cu-catalyzed etherification and aldol condensation with the respective bottom subunit in the presence of CuBr and Cs_2_CO_3_.

### 2.3. Biological Activity

All the novel synthesized benzo[6,7]oxepino[4,3,2-*cd*]isoindol-2(1*H*)-one derivatives were first evaluated for inhibitory activity of Brd4 bromodomain enzyme at a concentration of 10 µM. IC_50_ value of iBet762 in our Brd4 enzyme assay was 0.15–0.30 µM. The structures of these compounds are presented in Table 1, Table 2, Table 3 and Table 4 along with the results of alpha assay and cell cytotoxicity assay. Ty82, which expresses NUT- Brd4 fusion protein, is used for cytotoxicity assay, because the proliferation of Ty82 is dependent on Brd4 activity. Notably, three compounds (**8ac**, **8ad,** and **8af**) demonstrated an important pattern of inhibitory activity. **8ad** with a bulky aromatic group at the R^3^ position in the presence of 3-phenoxypropane had an IC_50_ of 7.08 µM, whereas **8ac** with 4-methoxyphenethyl group displayed a two-fold increased IC_50_ value. Replacement of R^1^ with ethoxy group (**8af**) instead of methoxy in **8ac** afforded a weak inhibitory effect. Evaluation of the activity of these three compounds revealed that it is important for the oxygen atom present in the functional group at the R^2^ position to be directed outward. Retaining ethoxy group at the R^1^ position and introducing methoxy group at the R^2^ and R^3^ positions (**8ae**) slightly increased the inhibitory activity compared with **8af**. 

The ethoxy substituent in **8ab** in the middle of the scaffold resulted in four-fold increased activity compared with **8ae**, indicating the importance of size and position of functional groups on the benzo[6,7]oxepino[4,3,2-*cd*]isoindol-2(1*H*)-one scaffold in maintaining inhibitory activity. 

Notably, the activity increased in **8aa** (IC_50_ = 0.83 µM) in which the R^3^ functional group methoxy moiety was replaced with simple hydrogen atom (Table 1). From the results presented in Table 1, we confirmed that hydrogen atom, which has the smallest size, is the best group at R^2^ and R^3^ positions to maintain inhibitory activity. Therefore, in the second-round derivatization of compounds, hydrogen atom was substituted at R^2^ and R^3^ positions. Diverse derivatives were synthesized by fixing hydrogen atom at the R^2^ position (Table 2). We synthesized the derivatives of novel compounds by fixing R^2^ and R^3^ with hydrogen, and derivatization was performed with substitutions at R^4^ position. 

Compound **8ba** in which isobutoxy group was introduced showed an IC_50_ of 16.7 µM, but the activity dramatically increased 40-fold when the functional group was methoxy (**8bc)**. In compound **8bd**, which has a –OH group that functions as a hydrogen donor or acceptor, the IC_50_ values in enzyme assay and cell cytotoxicity assay were 0.41 and 0.54 µM, respectively. Notably, the inhibitory activity was maintained in compound **8be** (IC_50_ = 0.86 µM), where *N,N*-diethylaminoethoxy group was introduced as a hydrogen bond acceptor. Compound **8bf**, which has a pyridine group at a position adjacent to the oxygen atom at R^4^ position, showed inhibitory activity against bromodomain (IC_50_ = 0.91 µM), whereas the benzyl group-substituted compound **8bg** did not. 

Fluorine or trifluoromethyl group is widely used to improve metabolic stability in drug discovery and pharmaceutical science. We introduced the –CF_3_ group instead of oxygen atom at R^4^ position to assess the change in inhibitory activity. The replacement of methoxy group of **8bc** with –CF_3_ (**8bb**) showed no inhibitory activity. To confirm this dramatic reduction in biological activity, we synthesized R^1^ = ethoxy compounds (**8bj**) while maintaining the same functional groups at the R^4^ position. The presence of methoxy group at R^1^ position (**8bk**) displayed an IC_50_ of 0.86 µM, whereas the substitution of methoxy with trifluoromethyl group (**8bj**) reduced the inhibitory activity (IC_50_ = 9 µM) against bromodomain. Compounds **8bh** and **8bp** were synthesized to evaluate the activity of derivatives containing oxygen atom at positions 3 and 4 with methylene (–CH_2_–) linkage; both the compounds exhibited modest inhibition. Through the biological evaluation of compounds in Table 2, we found that compounds with alkoxy group—including hydroxyl, methoxy, isobutoxy, and pyridinmethoxy—at R^4^ position showed inhibitory activity. However, the presence of benzyloxy group at R^4^ position abolished the inhibitory effect against bromodomain.

The compounds presented in Table 3 were synthesized to evaluate their inhibitory activity by substituting nitrogen atom, an electron withdrawing group similar to oxygen atom and has stronger basicity, at the R^1^ position. Compound **8ca** in which isobutoxy group was introduced at the R^4^ position showed no inhibitory activity. Compound **8cb**, which had a hydroxyl group at the R^4^ position, displayed weaker inhibitory effects than **8bd** and **8bl**, which have methoxy or epoxy groups at R^1^ position. We reduced the double bond (C=C) presented in the oxepino ring located at the center of the benzo[6,7]oxepino[4,3,2-*cd*]isoindol-2(1*H*)-one scaffold (**8cc**). The inhibitory effect of **8cc** is similar to that of **8cb**. From the IC_50_ values of compounds **8ca**–**8ce** in Table 3, we found that an oxygen atom is suitable for the R^1^ position. 

The compounds presented in Table 4 were synthesized to study the inhibitory activity on bromodomain by introducing carbon chain instead of heteroatoms at R^1^ position. Considering the length of the substituent at R^1^ in the compounds presented in Table 1, Table 2 and Table 3, we substituted n-propyl group at the R^1^ position. Isobutoxy (**8da**), –CF_3_ (**8db**), pyridinemethoxy (**8dc**), and benzyloxy (**8dd**) groups were introduced at the R^4^ position. Compounds having a carbon chain at R^1^ showed similar inhibitory activity to compounds having methoxy or ethoxy at R^1^ position.

**Figure 3:**
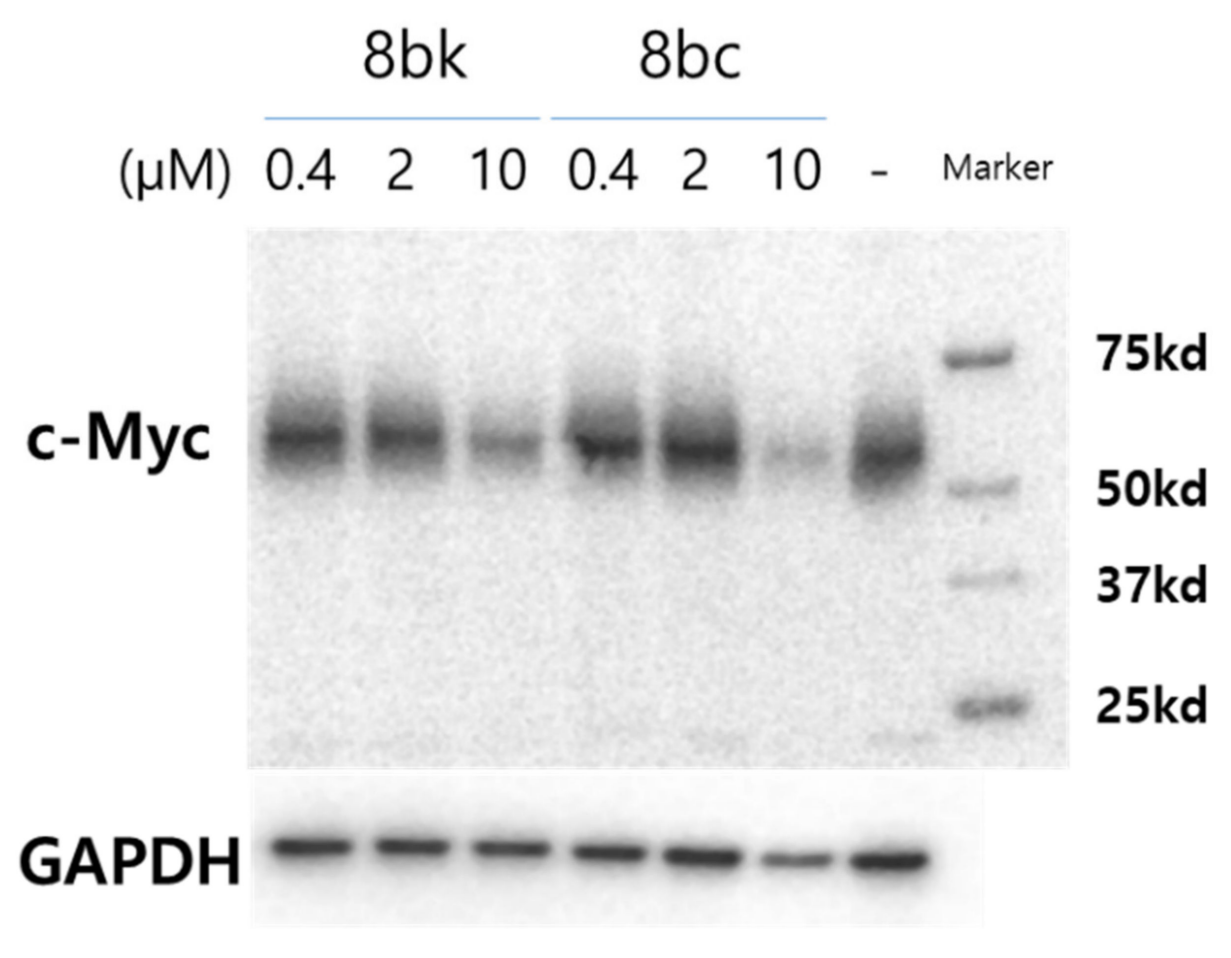
NALM6 cells were treated with **8bk** or **8bc** compounds. After 18 h, cell lysates were prepared, and western blot was conducted to see c-MYC level.

It is well known that c-MYC expression is controlled by the binding of bromodomain to lysine-acetylated histone [17]. Our derivatives downregulated the expression of c-MYC. As shown in Figure 3, c-MYC level was significantly decreased in **8bk**- or **8bc**-treated B-cell lymphoma cell line, NALM6. Our data clearly shows aristoyagonine derivatives with good activity against Brd4 have excellent cytotoxicity against Ty82.

### 2.4. Crystal Structures of Brd4 Bromodomain in Complex with Aristoyagonine Derivatives 

To verify the specific binding of inhibitors to Brd4 bromodomain and obtain the structural information to develop further optimized inhibitors, we determined five three-dimensional structures of Brd4 bromodomain–inhibitor complexes (Table S1). The overall structure of BRD4 bromodomain comprises four α-helices connected by highly variable loops, such as BC and ZA loops (Supplementary Figure S1). These two loops and their neighboring regions form a highly hydrophobic pocket, which is known to be the acetylated lysine binding pocket [9]. All electron density maps for the five compounds were found in the acetylated lysine binding pocket including JQ-1 and I-BET762 derivatives, L5S and H5C, bound bromodomain structures (Supplementary Figure S1) [9,25].

### 2.5. Structural Comparison of Aristoyagonine Derivatives with Other Known Brd4 Bromodomain Inhibitors

**Figure 4:**
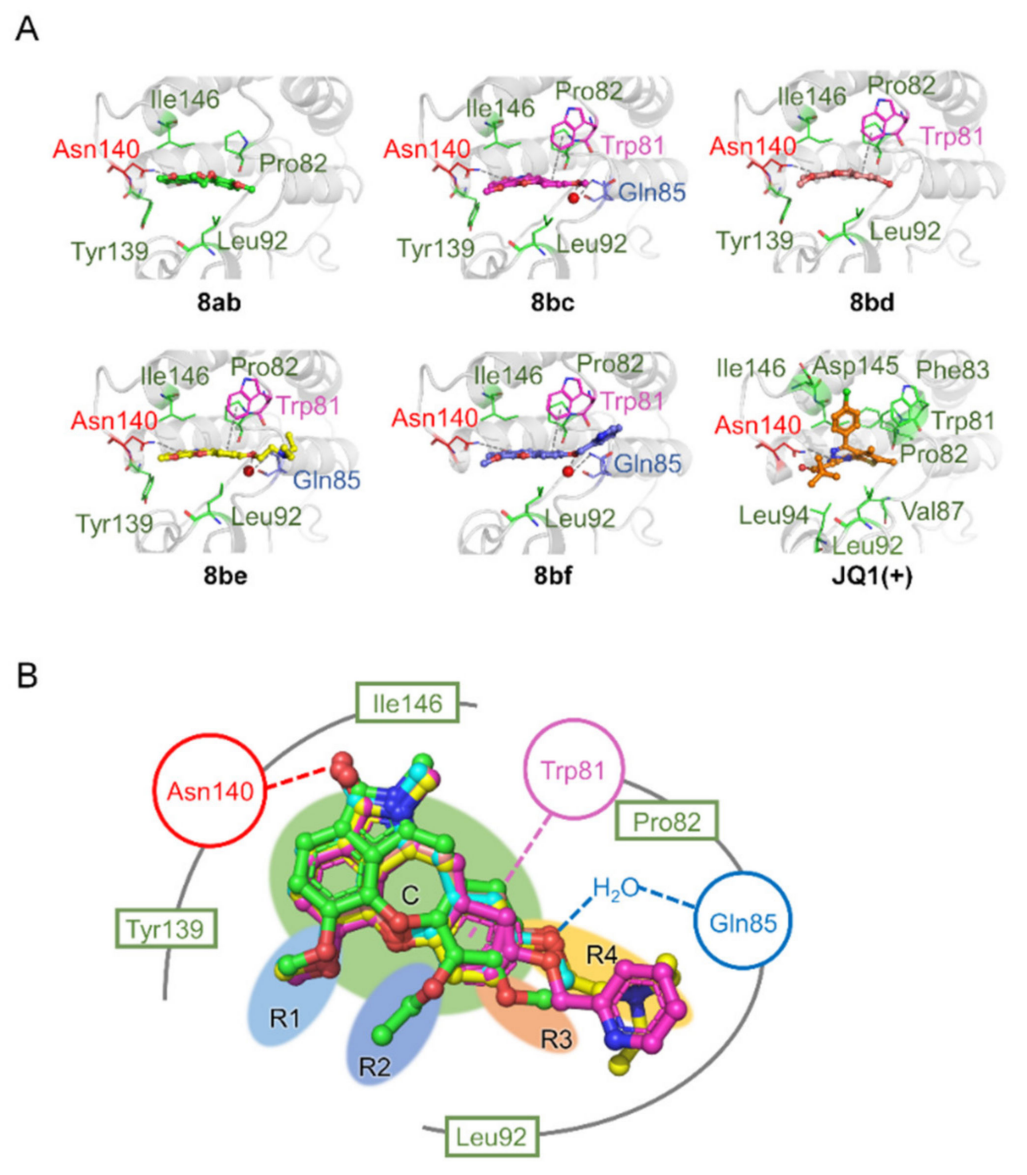
Binding modes of the five benzo[6,7]oxepino[4,3,2-*cd*]isoindol-2(1*H*)-one inhibitors and JQ-1. (**A**) Detailed view of binding modes of the five benzo[6,7]oxepino[4,3,2-*cd*]isoindol-2(1*H*)-one inhibitors (**8ab**, **8bc**, **8bd**, **8be**, and **8bf**) and JQ-1 (PDB entry:3MXF). In each figure, the residues that interacted with each inhibitor are also presented. Residues interacting with the inhibitors are indicated in green, red, blue, and purple: Hydrophilic residues are indicated in green; the residue of Asn140 forms hydrogen bond is presented in red; the water bridge in ocean blue; π-stacking is presented by purple. (**B**) Overlapped views of the five benzo[6,7]oxepino[4,3,2-*cd*]isoindol-2(1*H*)-one inhibitors are presented along with the interactions between each inhibitor and protein residues. The core structure C of inhibitors and chemical moieties (R^1^–R^4^) are shown.

In this structural study, all five novel compounds formed a hydrogen bond with the amine group of Asn140 and oxygen atom of the carbonyl group in the core structure (benzo[6,7]oxepino[4,3,2-*cd*]isoindol-2(1*H*)-one) of the synthesized compounds (Figure 4). This hydrogen bond has been found in most of the bromodomain binding inhibitors, including JQ-1 and I-BET762 derivatives; Asn140 has been known as the key residue for acetylated lysine interaction [9,24]. In addition, these inhibitors form several hydrophobic interactions with Pro82, Leu92, Tyr139, and Ile146 (Figure 4). However, the five benzo[6,7]oxepino[4,3,2-*cd*]isoindol-2(1*H*)-one inhibitors formed fewer hydrophobic interactions with protein residues than JQ-1, which is likely due to the chemical structural planarity of inhibitors compared with JQ-1. The inhibitors adopt relatively large planar chemical structures in the core scaffold of inhibitors (Supplementary Figure S1), and the other moieties (functional groups in R^1^–R^4^) are relatively small. In contrast, JQ-1 has a relatively small planar structure in the core of the inhibitor (13-methyl-3-thia-1,8,11,12-tetraazatricyclo[8.3.0.02,6]trideca-2(6),4,7,10,12-pentaene). Moreover, two other bulk moieties of JQ-1 are stretched out, fully occupying the whole acetylated lysine binding pocket and forming additional hydrophobic interactions with the protein residues Phe83, Val87, Leu94, and Asp145 around the Kac binding pocket (Figure 4A) [9].

In contrast to **8ab** structure, the structures of **8bc**, **8bf**, and **8be** contain an ordered water molecule forming a water-mediated interaction between the side chain amine of Gln85 and oxygen atom of 6-ether at R^4^ position. The position of R^4^ seems to be critical for these water-mediated interactions, considering the absence of a corresponding water molecule in **8ab**, which has a methoxy group at R^3^ position instead of R^4^ position (Supplementary Figure S1). The formation of π-stacking interaction between phenol ring of the core structure and indole group of Trp81 is also a remarkable feature of these inhibitors. In JQ-1 and I-BET762 derivatives-bound Brd4 bromodomain structures, a methyl group (4-methyl) present at the corresponding region of the phenol ring in our inhibitors forms hydrophobic interaction with Trp81 instead of π-stacking in our inhibitors (Figure 4).

## 3. Materials and Methods

### 3.1. General

Unless otherwise stated, all reactions were performed under an inert (N_2_) atmosphere. Reagents and solvents were reagent grade and purchased from Sigma-Aldrich (Milwaukee, WI, USA), Alfa Aesar (Ward Hill, MA, USA) and TCI Tokyo (Tokyo, Japan). Flash column chromatography was performed using silica gel 60 (230–400 mesh, Merck, Darmstadt, Germany) with the indicated solvents. Thin-layer chromatography was performed using 0.25 mm silica gel plates (Merck). NMR spectra were obtained with a Bruker (Billerica, MA, USA) ultra-shield spectrometer at a ^1^H frequency of 300 MHz or 500 MHz and a ^13^C frequency of 125 MHz. Chemical shifts are reported in parts per million (ppm). Data for ^1^H NMR are reported as follows: chemical shift (δ ppm) (integration, multiplicity, coupling constant (Hz)). Multiplicities are reported as follows: s = singlet, d = doublet, t = triplet, q = quartet, m = multiplet. The residual solvent peak was used as an internal reference. The mass spectra were obtained on an AcouityTM waters A06UPD9BM (Santa Clara, CA, USA), and Agilent Technologies SG12109048 (Santa Clara, CA, USA). Prior to biological testing, final compounds were confirmed to be >95% pure by UPLC chromatography using a Waters ACQUITY H-class system fitted with a C18 reversed-phase column (ACQUITY UPLC BEH C18: 2.1 mm × 50 mm, Part No. 186002350) according to the following conditions with solvents (A) H_2_O + 0.1% formic acid, (B) CH_3_CN + 0.1% formic acid, (C) MeOH + 0.1% formic acid; (Ι) a gradient of 95% A to 95% B over 5 min; (II) a gradient of 95% A to 95% C over 5 min.

### 3.2. Alpha-Screen Enzyme Assay

Bromodomain was prepared as previously described. The alpha-screen assay was performed in accordance with the manufacturer’s protocol (PerkinElmer, Waltham, MA, USA), by using a buffer (50 mM HEPES, 100 mM NaCl, 0.1% BSA, pH 7.4 supplemented with 0.05% CHAPS) and OptiPlateTM-384 plate (PerkinElmer, USA). Briefly, 2.5 μL of compound solution and 5μL of peptide solution [SGRGK(Ac)GGK(Ac)GLGK(Ac)GGAK(Ac)RHRK-biotin] were added to 5 μL of glutathione-S-transferase (GST) and His-tagged bromodomain in OptiPlateTM-384 plate. Streptavidin-coated donor beads and anti-GST alpha-screen acceptor beads were added under low-light condition. Plate was incubated at 25 °C for 60 min using a Thermomixer C (Eppendorf, Hamburg, Germany), and read using a Fusion-Alpha™ Multilabel Reader (PerkinElmer, USA). The alpha-screen results were confirmed by using alpha-screen TruHit kits (PerkinElmer, USA).

### 3.3. Western Blot

For immunoblotting, cells were washed in PBS, lysed in 1× sample buffer (50 mmol/L Tris-HCl (pH 6.8), 10% glycerol, 2% SDS, and 3% β-mercaptoethanol), and boiled for 10 min. Lysates were subjected to SDS-PAGE followed by blotting with the indicated antibodies and detection by western blotting substrate ECL reagent (Thermo Fisher Scientific, Waltham, MA, USA). Images were produced using a SensiQ-2000 (Lugen Sci Co., Seoul, KOREA). The following antibodies were obtained from Cell Signaling Technology (Danvers, MA, USA): c-Myc (catalog no. 5605). Tubulin antibody (catalog no. T6199) was purchased from Sigma-Aldrich. HRP-conjugated anti-mouse (catalog no. NCI1430KR), and HRP-conjugated anti-rabbit (catalog no. NCI1460KR) antibodies were obtained from Thermo Scientific.

### 3.4. Cell Cytotoxic Assay

For the viability experiments, Ty82 cells were seeded in 96-well plates at 30% confluency and exposed to chemicals the next day. After 72 h, WST-1 reagent was added, and absorbance at 450 nm was measured by using a Spectramax spectrophotometer (Molecular Devices, San Jose, CA, USA) in accordance with the manufacturer’s instructions. The CC_50_ values were calculated by using GraphPad Prism version 5 for Windows. The curves were fitted using a nonlinear regression model with a log (inhibitor) versus response formula.

### 3.5. Protein Expression and Purification 

The Brd4 bromodomain gene (residues 44−168) was amplified by polymerase chain reaction and subcloned into the BamHI and XhoI site of pHis vector (a modified pET28b) vector. The resulting recombinant Brd4 bromodomain includes a TEV (Tobacco Etch Virus) protease cleavable polyhistidine (6×His) tag at the N-terminus. 

The recombinant protein was expressed in Escherichia coli BL21(DE3) cells (Merck Millipore, Burlinton, MA, USA) cultured in Terrific Broth. After grown to an OD_600_ of 0.6 at 37 °C, recombinant protein was induced with 0.1 mM isopropyl-d-thiogalactoside (IPTG) at 18 °C and cells were further cultured for about 15 h and harvested. Cells were resuspended in lysis buffer (50 mM HEPES-OH pH 7.5, 500 mM NaCl, 5% glycerol, 5 mM β-mercaptoethanol, and 1 mM phenylmethylsulfonyl fluoride) and lysed using high pressure homogenizer (PICOMAX, Seoul, Korea) for three times with 1000 bar pressure. After centrifugation at 13,000× *g* for 1 h, the supernatant was applied to Ni-NTA resin (GE healthcare, Chicago, IL, USA) and incubated with gentle agitation for 1 h under ice-cold condition. The Ni-NTA resin was washed using wash buffer (50 mM HEPES-OH pH 7.5, 500 mM NaCl, 5% glycerol, 5 mM β-mercaptoethanol, and 30 mM Imidazole) and protein was eluted with elution buffer (50 mM HEPES-OH pH 7.5, 500 mM NaCl, 5% glycerol, 5 mM β-mercaptoethanol, and 300 mM Imidazole). The eluted protein was incubated with TEV protease for overnight at 4 °C to remove the fusion tag. Protein was further purified using a HiLoad 16/600 Superdex 75 prep grade column (GE Healthcare, USA) with final storage buffer (10 mM HEPES-OH pH 7.5, 500 mM NaCl, 5% (*v*/*v*) glycerol, and 10 mM dithiothreitol). Protein was concentrated to 14 mg/mL using Amicon Centrifugal Filter Units (Merck Millipore, Burlinton, MA, USA) and stored at −80 °C for crystallization.

### 3.6. Crystallization and Structure Solution

All crystallization experiments were performed using the sitting drop vapor diffusion method at 14 °C. Before crystallization, the Brd4 bromodomain protein stock was diluted 10 times with the final storage buffer and added with 0.7 mM inhibitor compounds (approximately 1:10 protein:compound molar ratio) and incubated at 4 °C for overnight. The mixed protein solution was reconcentrated and mixed with crystallization reservoir solution (6 M sodium formate and 10% glycerol). The protein:reservior solution ratio was 1:1 (*v*/*v*) and crystals were grown within 3 days.

Diffraction data were collected using a Dectris Pilatus3 6M CCD detector at the BL-11C beamline of Pohang Light Source (Pohang, Korea). Crystals were protected by the cryo-buffer (crystallized reservoir solution supplemented with 2.5 mM of each inhibitor compound and 30% (*v*/*v*) glycerol). The raw data were processed and scaled using the HKL2000 program suite [26]. The phase was calculated by molecular replacement with the program PHASER [27] using a Brd4 bromodomain structure (PBD entry, 2OSS) as a search model [9]. Further model building was completed with the program COOT [28] and refinement was conducted using the phenix.refine in Phenix program suite [29]. The coordinates and cif restraint files of the inhibitors were generated using the program Maestro (Schrödinger, New York, NY, USA) and the eLBOW in Phenix program suite [30,31], respectively. All data collection and refinement statistics are listed in Table S1.

### 3.7. Synthesis 

Compounds **2a**–**e** [32]. To a flask containing a solution of 4-hydroxybenzaldehyde (**1**, 10.0 g) in acetone (150 mL) were added K_2_CO_3_ (1.2 eq.) and benzyl bromide (2.0 eq.). The reaction flask was stirred for 9 h at 70 °C. The solvent was removed with a rotary evaporator and transferred to a separatory funnel with H_2_O and CH_2_Cl_2_. The combined organic layers were dried over anhydrous MgSO_4_, filtered and concentrated in vacuum. The residue was purified by silica gel flash column chromatography to afford benzyloxybenzaldehyde (**2**).

*2-(Benzyloxy)-3-methoxybenzaldehyde* (**2a**). Rf 0.25 (hexane/EtOAc = 9/1). Yield = 82% (13.0 g). ^1^H-NMR (CDCl_3_, 300 MHz) δ 10.24 (1H, s, CHO), 7.40–7.32 (6H, m, *Ar*), 7.18–7.10 (2H, m, *Ar*), 5.17 (2H, s, CH_2_), 3.93 (3H, s, CH_3_); LCMS (ESI^+^) *m/z* calcd. for C_15_H_14_O_3_ ([M + H^+^]) 243.09, found 243.12

*2-(Benzyloxy)-3-ethoxybenzaldehyde* (**2b**). Rf 0.25 (hexane/EtOAc = 9/1). Yield = 89% (13.7 g). ^1^H-NMR (CDCl_3_, 300 MHz) δ 10.26 (1H, s, CHO), 7.41–7.33 (6H, m, *Ar*), 7.18–7.10 (2H, m, *Ar*), 5.19 (2H, s, CH_2_), 4.18–4.11 (2H, q, *J* = 6.9 Hz, CH_2_), 1.53–1.48 (3H, t, *J* = 6.9 Hz, CH_3_); LCMS (ESI^+^) *m/z* calcd. for C_16_H_16_O_3_ ([M + H^+^]) 256.11, found 256.17

*2-(Benzyloxy)-3-methylbenzaldehyde* (**2c**). Rf 0.29 (hexane/EtOAc = 9/1). Yield = 99% (17.5 g). ^1^H-NMR (CDCl_3_, 300 MHz) δ 10.26 (1H, s, CHO), 7.69 (1H, d, *J* = 7.7 Hz, *Ar*), 7.47 (1H, d, *J* = 7.4 Hz, *Ar*), 7.42–7.37 (5H, m, *Ar*), 7.17 (1H, t, *J* = 7.4 Hz, *Ar*), 4.96 (2H, s, CH_2_), 2.35 (3H, s, CH_3_); LCMS (ESI^+^) *m/z* calcd. for C_15_H_14_O_2_ ([M + H^+^]) 227.10, found 227.12

*3-Allyl-2-(benzyloxy)benzaldehyde* (**2d**). Rf 0.24 (hexane/EtOAc = 12/1). Yield = 84% (13.1 g). ^1^H-NMR (CDCl_3_, 300 MHz) δ 10.30 (1H, s, CHO), 7.78 (1H, m, *Ar*), 7.54 (1H, m, *Ar*), 7.45–7.39 (5H, m, *Ar*), 7.28 (1H, m, *Ar*), 6.01 (1H, m, CH), 5.17 (2H, m, CH_2_), 5.01 (2H, s, CH_2_), 3.53 (2H, m, CH_2_); LCMS (ESI^+^) *m/z* calcd. for C_17_H_16_O_2_ ([M + H^+^]) 253.12, found 253.14

*2-(Benzyloxy)-3-(diethylamino)benzaldehyde* (**2e**). Rf 0.28 (hexane/EtOAc = 7/3). Yield = 99% (14.5 g). ^1^H-NMR (CDCl_3_, 300 MHz) δ 10.27 (1H, s, CHO), 7.77 (1H, d, *J* = 8.9 Hz, *Ar*), 7.49–7.35 (5H, m, *Ar*), 6.29 (1H, dd, *J* = 8.9 Hz, *J* = 1.86 Hz, *Ar*), 6.08 (1H, d, *J* = 2.2 Hz, *Ar*), 5.19 (2H, s, CH_2_), 3.38 (4H, q, *J* = 7.1 Hz, 2×CH_2_), 1.17 (6H, t, *J* = 7.1 Hz, 2×CH_3_); LCMS (ESI^+^) *m/z* calcd. for C_18_H_21_NO_2_ ([M + H^+^]) 284.16, found 284.17

Compounds **3a**–**e** [24]. Benzaldehyde (**2**, 38.5 mmol) was mixed in methylamine (40% in MeOH, 25 mL, 0.19 mol) at r.t. under a N_2_ atmosphere. The mixture was stirred at r.t. for 48 h, until the benzylidene methanamine formation was completed. The methanol was removed with a rotary evaporator and the mixture was extracted with CH_2_Cl_2_ from H_2_O. The combined organic layers were washed with brine, dried over MgSO_4_. Straightly, benzylidene methanamine in MeOH was carefully treated with solid NaBH_4_ (2.18 g, 57.8 mmol) at 0 °C. The reaction flask was flushed with N_2_ and stirred at 0 °C for 1 h. The methanol was evaporated and partitioned between EtOAc and H_2_O. The organic layers were collected, dried over MgSO_4_. The solution was evaporated in vacuum and purified by silica gel flash column chromatography to afford methylamine derivatives (**3**).

*1-(2-(Benzyloxy)-3-methoxyphenyl)-N-methylmethanamine* (**3a**). Rf 0.22 (DCM/EtOAc = 9/1). Yield = 95% (9.9 g). ^1^H-NMR (CDCl_3_, 300 MHz) δ 7.44–7.32 (5H, m, *Ar*), 7.07–7.01 (1H, m, *Ar*), 6.92–6.88 (2H, m, *Ar*), 5.05 (2H, s, CH_2_), 3.89 (3H, s, CH_3_), 3.68 (2H, s, CH_2_), 2.32 (3H, s, CH_3_); LCMS (ESI^+^) *m/z* calcd. for C_18_H_21_NO_2_ ([M + H^+^]) 258.14, found 258.17

*1-(2-(Benzyloxy)-3-ethoxyphenyl)-N-methylmethanamine* (**3b**). Rf 0.22 (DCM/EtOAc = 9/1). Yield = 97% (14.0 g). ^1^H-NMR (CDCl_3_, 300 MHz) δ 7.50–7.47 (2H, m, *Ar*), 7.43–7.35 (3H, m, *Ar*), 7.06–7.01 (1H, m, *Ar*), 6.90–6.87 (2H, m, *Ar*), 5.08 (2H, s, CH_2_), 4.17 (2H, q, *J* = 6.9 Hz, CH_2_), 3.68 (2H, s, CH_2_), 2.34 (3H, s, CH_3_), 1.50 (3H, t, *J* = 6.9 Hz, CH_3_); LCMS (ESI^+^) *m/z* calcd. for C_17_H_21_NO_2_ ([M + H^+^]) 272.16, found 272.18

*1-(2-(Benzyloxy)-3-methylphenyl)-N-methylmethanamine* (**3c**). Rf 0.26 (DCM/EtOAc = 9/1). Yield = 88% (16.3 g). ^1^H-NMR (CDCl_3_, 300 MHz) δ 7.53–7.39 (5H, m, *Ar*), 7.22–7.15 (2H, m, *Ar*), 7.06 (1H, m, *Ar*), 4.91 (2H, s, CH_2_), 3.78 (2H, s, CH_2_), 2.42 (3H, s, CH_3_), 2.38 (3H, s, CH_3_); LCMS (ESI^+^) *m/z* calcd. for C_16_H_19_NO ([M + H^+^]) 242.1, found 242.7

*1-(3-Allyl-2-(Benzyloxyphenyl)-N-methylmethanamine* (**3d**). Rf 0.27 (DCM/EtOAc = 15/1). Yield = 81% (11.2 g). ^1^H-NMR (CDCl_3_, 300 MHz) δ 7.47–7.32 (5H, m, *Ar*), 7.25 (1H, m, *Ar*), 7.17–7.05 (2H, m, *Ar*), 6.06 (1H, m, CH), 5.10 (1H, m, CH), 5.05 (1H, m, CH), 4.88 (2H, s, CH_2_), 3.78 (2H, s, CH_2_), 3.46 (2H, m, CH_2_), 2.39 (3H, s, CH_3_); LCMS (ESI^+^) *m/z* calcd. for C1_8_H_21_NO ([M + H^+^]) 268.2, found 268.7

*2-(Benzyloxy)-N,N-diethyl-3-((methylamino)methylaniline* (**3e**). Rf 0.20 (DCM/EtOAc = 6/1). Yield = 84% (12.7 g). ^1^H-NMR (CDCl_3_, 300 MHz) δ 7.44–7.30 (5H, m, *Ar*), 7.10 (1H, m, *Ar*), 6.23 (2H, m, *Ar*), 5.10 (2H, s, CH_2_), 3.75 (2H, s, CH_2_), 3.30 (4H, q, *J*= 7.0 Hz, 2×CH_2_), 2.38 (3H, s, CH_3_), 1.10 (6H, t, *J* = 7.0 Hz, 2×CH_3_); LCMS (ESI^+^) *m/z* calcd. for C_19_H_26_N_2_O ([M + H^+^]) 299.2, found 299.6

Compounds **4a**–**e** [24]. A stirred mixture of freshly prepared amine (**3**, 40.4 mmol), Pd(OAc)_2_ (0.9 g, 4.05 mmol), and Cu(OAc)_2_ (7.3 g, 40.5 mmol) in toluene (130 mL) was refluxed in an oil bath at 120 °C in an atmosphere of carbon monoxide (ca. 1~1.5L) containing air (corresponding to 0.3 mmol of O_2_) delivered from a balloon for 20 h. After cooled to room temperature, the mixture was diluted with EtOAc and filtered through a short silica gel pad. The solution was concentrated in vacuo, and toluene was removed in a rotary evaporator. The residue was purified by silica gel flash column chromatography to afford 2-methylisoinolin-1-one (**4**). 

*4-(Benzyloxy)-5-methoxy-2-methylisoindolin-1-one* (**4a**). Rf 0.23 (hexane/EtOAc = 5/1). Yield = 60.4% (6.6 g). ^1^H-NMR (CDCl_3_, 300 MHz) δ 7.54 (1H, d, *J* = 8.2 Hz, *Ar*), 7.37–7.26 (5H, m, *Ar*), 7.03 (1H, d, *J* = 8.2 Hz, Ar), 5.11 (2H, s, CH_2_), 4.02 (2H, s, CH_2_), 3.96 (3H, s, CH_3_), 3.07 (3H, s, CH_3_); LCMS (ESI^+^) *m/z* calcd. for C_17_H_17_NO_3_ ([M + H^+^]) 284.1, found 284.3

*4-(Benzyloxy)-5-ethoxy-2-methylisoindolin-1-one* (**4b**). Rf 0.23 (hexane/EtOAc = 5/1). Yield = 84% (12.9 g). ^1^H-NMR (CDCl_3_, 300 MHz) δ 7.53 (1H, d, *J* = 8.2, *Ar*), 7.38–7.33 (5H, m, *Ar*), 7.03 (1H, d, *J* = 8.2, *Ar*), 5.13 (2H, s, CH_2_), 4.21 (2H, q, *J* = 6.9 Hz, CH_2_), 4.05 (2H, s, CH_2_), 3.08 (3H, s, CH_3_), 1.51 (3H, t, *J* = 6.9 Hz, CH_3_); LCMS (ESI^+^) *m/z* calcd. for C_18_H_19_NO_3_ ([M + H^+^]) 298.2, found 298.5

*4-(Benzyloxy)-5-methyl-2-methylisoindolin-1-one* (**4c**). Rf 0.28 (hexane/EtOAc = 5/1). Yield = 62% (11.3 g). ^1^H-NMR (CDCl_3_, 300 MHz) δ 7.50 (1H, d, *J* = 7.4 Hz, *Ar*), 7.41–7.37 (5H, m, *Ar*), 7.29 (1H, d, *J* = 7.4 Hz, *Ar*), 5.01 (2H, s, CH_2_), 4.22 (2H, s, CH_2_), 3.12 (3H, s, CH_3_), 2.37 (3H, s, CH_3_); LCMS (ESI^+^) *m/z* calcd. for C_17_H_17_NO_2_ ([M + H^+^]) 268.2, found 268.2

*5-Allyl-4-(benzyloxy)-2-methylisoindolin-1-one* (**4d**). Rf 0.32 (hexane/EtOAc = 6/1). Yield = 73% (8.9 g). ^1^H-NMR (CDCl_3_, 300 MHz) δ 7.58 (1H, d, *J* = 7.6 Hz, *Ar*), 7.42–7.40 (5H, m, *Ar*), 7.34 (1H, d, *J* = 7.6 Hz, *Ar*), 6.00 (1H, m, CH), 5.14 (2H, m, CH_2_), 5.05 (2H, s, CH_2_), 4.29 (2H, s, CH_2_), 3.53 (2H, m, CH_2_), 3.17 (3H, s, CH_3_); LCMS (ESI^+^) *m/z* calcd. for C_19_H_19_NO_2_ ([M + H^+^]) 294.2, found 294.3

*4-(Benzyloxy)-5-(diethylamino)-2-methylisoindolin-1-one* (**4e**). Rf 0.18 (hexane/EtOAc = 6/4). Yield = 32% (4.5 g). ^1^H-NMR (CDCl_3_, 300 MHz) δ 7.46–7.41 (5H, m, *Ar*), 6.76 (1H, d, *J* = 1.9 Hz, *Ar*), 6.37 (1H, d, *J* = 1.9 Hz, *Ar*), 5.16 (2H, s, CH_2_), 4.29 (2H, s, CH_2_), 3.36 (4H, q, *J* = 7.0 Hz, 2×CH_2_), 3.19 (3H, s, CH_3_), 1.14 (6H, t, *J* = 7.0 Hz, 2×CH_3_); LCMS (ESI^+^) *m/z* calcd. for C_20_H_24_N_2_O_2_ ([M + H^+^]) 325.2, found 325.7

Compounds **5a**–**e** [24]. To a solution of methylisoindolin-1-one (**4**, 6.70 mmol) in MeOH/EtOAc (1:1, 20 mL) was added Pd/C (10 wt %). The reaction mixture was stirred under 40 psi until the absorption of hydrogen ceased for 4 h. After the Pd/C catalyst was filtered off through a Celite pad, the solvent was removed on a rotary evaporator. The mixture was transferred to a separatory funnel with CH_2_Cl_2_. The organic layers were washed with brine, dried over MgSO_4_. The residue was treated with ether and resulting solid was collected by filtration to afford isoindolinone (**5**). 

*4-Hydroxy-5-methoxy-2-methylisoindolin-1-one* (**5a**). Rf 0.22 (DCM/MeOH = 19/1). Yield = 92.6% (4.2 g). ^1^H-NMR (CDCl_3_, 300 MHz) δ 7.39 (1H, d, *J* = 8.1 Hz, *Ar*), 6.97 (1H, d, *J* = 8.1 Hz, *Ar*), 5.82 (1H, s, OH), 4.34 (2H, s, CH_2_), 3.96 (3H, s, CH_3_), 3.17 (3H, s, CH_3_); LCMS (ESI^+^) *m/z* calcd. for C_10_H_11_NO_3_ ([M + H^+^]) 194.0, found 194.1

*4-Hydroxy-5-ethoxy-2-methylisoindolin-1-one* (**5b**). Rf 0.23 (DCM/MeOH = 19/1). Yield = 90% (8.1 g). ^1^H-NMR (DMSO-*d_6_*, 300 MHz) δ 7.11 (1H, d, *J* = 8.1 Hz, Ar), 7.05 (1H, d, *J* = 8.1 Hz, Ar), 4.30 (2H, s, CH_2_), 4.12 (2H, q, *J* = 6.9 Hz, CH_2_), 3.02 (3H, s, CH_3_), 1.36 (3H, t, *J* = 6.9 Hz, CH_3_); LCMS (ESI^+^) *m/z* calcd. for C_11_H_13_NO_3_ ([M + H^+^]) 208.1, found 208.1

*4-Hydroxy-5-methyl-2-methylisoindolin-1-one* (**5c**). Rf 0.25 (DCM/MeOH = 19/1). Yield = 81% (6.1 g). ^1^H-NMR (DMSO-*d_6_*, 300 MHz) δ 9.37 (1H, br, OH), 7.20 (1H, d, *J* = 7.4 Hz, *Ar*), 7.06 (1H, d, *J* = 7.4 Hz, *Ar*), 4.33 (2H, s, CH_2_), 3.05 (3H, s, CH_3_), 2.24 (3H, s, CH_3_); LCMS (ESI^+^) *m/z* calcd. for C_10_H_11_NO_2_ ([M + H^+^]) 178.0, found 178.2

*5-allyl-4-hydroxy-2-methylisoindolin-1-one* (**5d**). Rf 0.20 (DCM/MeOH = 17/1). Yield = 76% (4.7 g). ^1^H-NMR (DMSO-*d_6_*, 300 MHz) δ 9.34 (1H, br, OH), 7.18 (1H, d, *J* = 7.5 Hz, *Ar*), 7.09 (1H, d, *J* = 7.5 Hz, *Ar*), 4.34 (2H, s, CH_2_), 3.05 (3H, s, CH_3_), 2.61 (2H, t, *J* = 7.2 Hz, CH_2_), 1.58 (2H, m, CH_2_), 0.90 (3H, t, *J* = 7.2 Hz, CH_3_); LCMS (ESI^+^) *m/z* calcd. for C_12_H_13_NO_2_ ([M + H^+^]) 204.1, found 204.3

*5-(Diethylamino)-4-hydroxy-2-methylisoindolin-1-one* (**5e**). Rf 0.15 (DCM/MeOH = 15/1). Yield = 38% (1.2 g). ^1^H-NMR (DMSO-*d_6_*, 300 MHz) δ 9.59 (1H, br, OH), 6.38 (1H, d, *J* = 1.8 Hz, Ar), 6.31 (1H, d, *J* = 1.8 Hz, Ar), 4.18 (2H, s, CH_2_), 3.32 (4H, q, *J* = 6.9 Hz, 2×CH_2_), 3.03 (3H, s, CH_3_), 1.09 (6H, t, *J* = 6.9 Hz, 2×CH_3_); LCMS (ESI^+^) *m/z* calcd. for C_13_H_18_N_2_O_2_ ([M + H^+^]) 235.0, found 235.3

#### 3.7.1. Alkylation of 2-Bromo-3-Hydroxybenzaldehyde (Bottom Subunit) 

2-Bromo-3-hydroxybenzaldehyde (2.0 g, 8.65 mmol, 1.0 eq.), potassium carbonate (2.4 g, 17.3 mmol) and alkyl halide (1.2 eq.) were dissolved in DMF (5 mL) and stirred overnight at room temperature. The reaction mixture was diluted with ethyl acetate and washed with water three times. The organic layer was concentrated and purified by column chromatography.

*2-Bromo-4-methoxy-3-(4-methoxyphenethoxy)benzaldehyde* (**7a**). Rf 0.23 (hexane/EtOAc = 7/3). Yield = 82.9% (2.6 g). ^1^H-NMR (CDCl_3_, 300 MHz) δ 10.2 (1H, s, CHO), 7.75 (1H, d, *J* = 8.7 Hz, *Ar*), 7.25 (2H, d, *J* = 8.5 Hz, *Ar*), 6.96 (1H, d, *J* = 8.7 Hz, *Ar*), 6.89 (2H, d, *J* = 8.5 Hz, *Ar*), 4.22 (2H, t, *J* = 7.3 Hz, CH_2_), 3.92 (3H, s, CH_3_), 3.81 (3H, s, CH_3_), 3.16 (2H, t, *J* = 7.3 Hz, CH_2_); LCMS (ESI^+^) *m/z* calcd. for C_17_H_17_BrO_4_ ([M + H^+^]) 365.3, found 365.7

*2-Bromo-4-methoxy-3-(3-phenoxypropoxy)benzaldehyde* (**7b**). Rf 0.23 (hexane/EtOAc = 7/3). Yield = 73.4% (2.3 g). ^1^H-NMR (CDCl_3_, 300 MHz) δ 10.2 (1H, s, CHO), 7.76 (1H, d, *J* = 8.6 Hz, *Ar*), 7.34 (2H, m, *Ar*), 6.99 (4H, m, *Ar*), 4.33 (2H, t, *J* = 6.0 Hz, CH_2_), 4.25 (2H, t, *J* = 5.9 Hz, CH_2_), 3.85 (3H, s, CH_3_), 2.34 (2H, m, CH_2_); LCMS (ESI^+^) *m/z* calcd. for C_17_H_17_BrO_4_ ([M + H^+^]) 365.1, found 365.3

#### 3.7.2. Typical Procedure for Direct ‘One-Pot’ Synthesis of Dibenzoxepine Lactam

A nitrogen-flushed microwave vial was equipped with a magnetic stirring bar and charged with 4 Å molecular sieves. The vial was flame dried for 10 min under high vacuum and purged with N_2_. After cooling to room temperature, isoindolinone (0.3 g), 2-bromobenzaldehyde (2.0 eq.), CuCl (0.10 eq.), and Cs_2_CO_3_ (3.0 eq.) were added sequentially. The reaction mixture was suspended in pyridine (10 mL). Then, the reaction vial was sealed and placed into a heating block at 150 °C for 24 h. The mixture was cooled to room temperature, filtered through a Celite pad, and washed with acetone. The resulting solution was concentrated with a rotary evaporator, and the residue was purified by silica gel flash column chromatography to afford dibenzoxepine lactam.

*5,7-Dimethoxy-1-methylbenzo[6,7]oxepino[4,3,2-cd]isoindol-2(1H)-one* (**8aa**). Rf 0.25 (hexane/EtOAc = 6/4). Yield = 31% (150 mg). Yellow solid. mp: 208–210 °C; ^1^H-NMR (CDCl_3_, 300 MHz) δ 7.37 (1H, d, *J* = 8.1 Hz, *Ar*), 6.97–6.92 (2H, m, *Ar*), 6.84 (1H, m, *Ar*), 6.61–6.58 (1H, m, *Ar*), 5.78(1H, s, *Ar),* 3.97 (3H, s, CH_3_), 3.91 (3H, s, CH_3_), 3.25 (3H, s, CH_3_). ^13^C-NMR (125 MHz, CDCl_3_) δ 166.3, 152.1, 151.8, 142.6, 142.0, 137.8, 128.9, 127.8, 125.1, 123.3, 121.9, 118.9, 115.0, 113.3, 107.9, 56.9, 56.6, 25.6. LCMS (ESI^+^) *m/z* calcd. for C_18_H_16_NO_4_ ([M + H^+^]) 309.3, found 309.9

*7-Ethoxy-5,8-dimethoxy-1-methylbenzo[6,7]oxepino[4,3,2-cd]isoindol-2(1H)-one* (**8ab**). Rf 0.25 (hexane/EtOAc = 6/4). Yield = 17% (96 mg). Light yellow solid. mp: 194–196 °C; ^1^H-NMR (CDCl_3_, 300 MHz) δ 7.36 (1H, d, *J* = 8.2 Hz, *Ar*), 6.96 (1H, d, *J* = 8.2 Hz, *Ar*), 6.64 (1H, d, *J* = 8.7 Hz, *Ar*), 6.56 (1H, d, *J* = 8.6 Hz, *Ar*), 5.73 (1H, s, *Ar*), 4.13 (2H, q, *J* = 7.0 Hz, CH_2_), 3.96 (3H, s, CH_3_), 3.85 (3H, s, CH_3_), 3.25 (3H, s, CH_3_), 1.48 (3H, t, *J* = 7.0 Hz, CH_3_). ^13^C-NMR (125 MHz, CDCl_3_) δ 166.1, 155.2, 151.9, 148.1, 141.4, 140.6, 135.3, 127.3, 125.8, 121.9, 121.4, 118.7, 114.6, 108.3, 107.8, 69.4, 56.5, 56.1, 25.6, 15.5. LCMS (ESI^+^) *m/z* calcd. for C_20_H_20_NO_5_ ([M + H^+^]) 353.4, found 353.9

*5,8-Dimethoxy-7-(4-methoxyphenethoxy)-1-methylbenzo[6,7]oxepino[4,3,2-cd]isoindol-2(1H)-one* (**8ac**). Rf 0.20 (hexane/EtOAc = 7/3). Yield = 12% (84 mg). Orange solid. mp: 182–184 °C; ^1^H-NMR (CDCl_3_, 300 MHz) δ 7.36 (1H, d, *J* = 8.2 Hz, *Ar*), 7.28 (2H, m, *Ar*), 6.96 (1H, d, *J* = 8.2 Hz, *Ar*), 6.87–6.83 (2H, m, *Ar*), 6.66 (1H, d, *J* = 8.7 Hz, *Ar*), 6.54 (1H, d, *J* = 8.6 Hz, *Ar*), 5.71 (1H, s, *Ar*), 4.23 (2H, t, *J* = 7.6 Hz, CH_2_), 3.93 (3H, s, CH_3_), 3.82 (3H, s, CH_3_), 3.80 (3H, s, CH_3_), 3.24 (3H, s, CH_3_), 3.18 (2H, t, *J* = 7.7 Hz, CH_2_). ^13^C-NMR (125 MHz, CDCl_3_) δ 165.9, 158.0, 154.9, 151.8, 148.0, 141.3, 140.5, 135.1, 130.4, 129.8, 127.0, 126.0, 121.8, 121.3, 118.5, 114.5, 113.7, 108.3, 107.7, 74.4, 56.4, 56.0, 55.2, 35.5, 25.5. LCMS (ESI^+^) *m/z* calcd. for C_27_H_26_NO_6_ ([M + H^+^]) 459.5, found 459.9.

*5,8-Dimethoxy-1-methyl-7-(3-phenoxypropoxy)benzo[6,7]oxepino[4,3,2-cd]isoindol-2(1H)-one* (**8ad**). Rf 0.26 (hexane/EtOAc = 7/3). Yield = 23% (94 mg). Pale yellow solid. mp: 189–191 °C; ^1^H-NMR (CDCl_3_, 300 MHz) δ 7.35 (1H, d, *J* = 8.1 Hz, *Ar*), 7.30 (1H, m, *Ar*), 6.95–6.90 (5H, m, *Ar*), 6.65 (1H, d, *J* = 8.7 Hz, *Ar*), 6.51 (1H, d, *J* = 8.7 Hz, *Ar*), 5.71 (1H, s, *Ar*), 4.30 (4H, m, 2×CH_3_), 3.89 (6H, m, 2×CH_3_), 3.71 (3H, s, CH_3_), 3.23 (3H, s, CH_3_), 2.31 (2H, m, CH_2_). LCMS (ESI^+^) *m/z* calcd. for C_27_H_26_NO_6_ ([M + H^+^]) 459.5, found 460.0

*5-Ethoxy-7,8-dimethoxy-1-methylbenzo[6,7]oxepino[4,3,2-cd]isoindol-2(1H)-one* (**8ae**). Rf 0.25 (hexane/EtOAc = 6/4). Yield = 18% (90 mg). Light yellow solid. mp: 219–220 °C; ^1^H-NMR (CDCl_3_, 300 MHz) δ 7.34 (1H, d, *J* = 8.1Hz, *Ar*), 6.94 (1H, d, *J* = 8.2 Hz, *Ar*), 6.67 (1H, d, *J* = 8.6 Hz, *Ar*), 6.55 (1H, d, *J* = 8.6 Hz, *Ar*), 5.72 (1H, s, *Ar*), 4.16 (2H, q, *J* = 6.9 Hz, CH_2_), 3.96 (3H, s, CH_3_), 3.86 (3H, s, CH_3_), 3.23 (3H, s, CH_3_), 1.52 (3H, t, *J* = 6.9 Hz, CH_3_). ^13^C-NMR (125 MHz, CDCl_3_) δ 166.1, 154.8, 151.4, 148.1, 141.6, 141.4, 135.4, 127.3, 125.8, 121.8, 121.5, 118.7, 115.8, 108.2, 107.6, 65.1, 61.4, 56.1, 25.6, 14.8. LCMS (ESI^+^) *m/z* calcd. for C_20_H_20_NO_5_ ([M + H^+^]) 353.4, found 353.9

*5-Ethoxy-8-methoxy-7-(4-methoxyphenethoxy)-1-methylbenzo[6,7]oxepino[4,3,2-cd]isoindol-2(1H)-one* (**8af**). Rf 0.24 (hexane/EtOAc = 7/3). Yield = 43% (290 mg). Light yellow solid. mp: 189–191 °C; ^1^H-NMR (CDCl_3_, 300 MHz) δ 7.30 (1H, d, *J* = 8.2 Hz, *Ar*), 7.20–7.17 (2H, m, *Ar*), 6.90 (1H, d, *J* = 8.2 Hz, *Ar*), 6.84–6.81 (2H, m, *Ar*), 6.62 (1H, d, *J* = 8.6 Hz, *Ar*), 6.50 (1H, d, *J* = 8.6 Hz, *Ar*), 5.67 (1H, s, *Ar*), 4.28 (2H, t, *J* = 7.6 Hz, CH_2_), 4.14 (2H, q, *J* = 6.8 Hz, CH_2_), 3.78–3.76 (6H, m, 2×CH_3_), 3.21 (3H, s, CH_3_), 3.11 (2H, t, *J* = 7.5 Hz, CH_2_), 1.47 (3H, t, *J* = 6.9 Hz, CH_3_). ^13^C-NMR (125 MHz, CDCl_3_) δ 166.0, 158.0, 154.9, 151.2, 148.2, 141.5, 140.5, 135.2, 130.4, 129.9, 127.0, 125.9, 121.7, 121.2, 118.4, 115.7, 113.7, 108.4, 107.6, 74.5, 65.1, 56.0, 55.2, 35.6, 25.5, 14.9. LCMS (ESI^+^) *m/z* calcd. for C_28_H_28_NO_6_ ([M + H^+^]) 473.5, found 474.0

*9-Isobutoxy-5-methoxy-1-methylbenzo[6,7]oxepino[4,3,2-cd]isoindol-2(1H)-one* (**8ba**). Rf 0.26 (hexane/EtOAc = 6/4). Yield = 42% (230 mg). Light yellow solid. mp: 187–189 °C; ^1^H-NMR (CDCl_3_, 300 MHz) δ 7.35 (1H, d, *J* = 8.1 Hz, *Ar*), 6.96–6.93 (2H, m, *Ar*), 6.66 (1H, dd, *J* = 8.79 Hz, 2.9 Hz, *Ar*), 6.53 (1H, d, *J* = 2.9 Hz, *Ar*), 5.73 (1H, s, *Ar*), 3.93 (3H, s, CH_3_), 3.68 (2H, d, *J* = 6.5 Hz, CH_2_), 3.24 (3H, s, CH_3_), 2.09–2.04 (1H, m, CH), 1.02 (3H, s, CH_3_), 1.00 (3H, s, CH_3_). ^13^C-NMR (125 MHz, CDCl_3_) δ 166.2, 156.3, 151.8, 147.0, 142.3, 137.9, 128.4, 127.1, 122.9, 122.0, 118.3, 117.0, 114.9, 114.3, 107.5, 74.8, 56.7, 28.2, 25.6, 19.2 LCMS (ESI^+^) *m/z* calcd. for C_21_H_22_NO_4_ ([M + H^+^]) 351.4, found 352.0

*5-Methoxy-1-methyl-9-(trifluoromethyl)benzo[6,7]oxepino[4,3,2-cd]isoindol-2(1H)-one* (**8bb**). Rf 0.27 (hexane/EtOAc = 6/4). Yield = 46% (250 mg). Light orange solid. mp: 190–191 °C; ^1^H-NMR (CDCl_3_, 300 MHz) δ 7.36–7.33 (2H, m, *Ar*), 7.21 (1H, s, *Ar*), 7.08 (1H, d, *J* = 8.4 Hz, *Ar*), 6.97 (1H, d, *J* = 8.2 Hz, *Ar*), 5.71 (1H, s, *Ar*), 3.94 (3H, s, CH_3_), 3.24 (3H, s, CH_3_). ^13^C-NMR (125 MHz, CDCl_3_) δ 166.0, 155.9, 151.8, 141.3, 138.6, 128.3, 128.2, 127.8, 126.6, 126.2, 124.7, 122.7, 122.5, 121.8, 119.0, 115.1, 56.6, 25.6. LCMS (ESI^+^) *m/z* calcd. for C_18_H_13_F_3_NO_3_ ([M + H^+^]) 347.3, found 347.9

*5,9-Dimethoxy-1-methylbenzo[6,7]oxepino[4,3,2-cd]isoindol-2(1H)-one* (**8bc**). Rf 0.20 (DCM/MeOH = 19/1). Yield = 23% (214 mg). Light orange solid. mp: 194–196 °C;^1^H-NMR (CDCl_3_, 300 MHz) δ 7.37 (1H, d, *J* = 8.1 Hz, *Ar*), 6.98–6.95 (2H, m, *Ar*), 6.69 (1H, dd, *J* = 8.7 Hz, 3.06 Hz, *Ar*), 6.54 (1H, d, *J* = 3.0 Hz, *Ar*), 5.76 (1H, s, *Ar*), 3.96 (3H, s, CH_3_), 3.79 (3H, s, CH_3_), 3.27 (3H, s, CH_3_). ^13^C-NMR (125 MHz, CDCl_3_) δ 166.1, 156.5, 151.8, 147.2, 142.1, 138.0, 128.4, 126.9, 122.9, 121.9, 118.3, 116.4, 114.8, 113.6, 107.4, 56.6, 55.5, 25.5. LCMS (ESI^+^) *m/z* calcd. for C_18_H_16_NO_4_ ([M + H^+^]) 309.3, found 309.9

*9-Hydroxy-5-methoxy-1-methylbenzo[6,7]oxepino[4,3,2-cd]isoindol-2(1H)-one* (**8bd**). Rf 0.28 (hexane/EtOAc = 6/4). Yield = 91% (210 mg). Light yellow solid. mp: 177–179 °C; ^1^H-NMR (CDCl_3_, 300 MHz) δ 7.38 (1H, d, *J* = 8.3 Hz, *Ar*), 6.99–6.93 (2H, m, *Ar*), 6.58 (1H, dd, *J* = 8.5 Hz, 2.9 Hz, *Ar*), 6.51 (1H, d, *J* = 3.1 Hz, *Ar*), 5.73 (1H, s, *Ar*), 4.79 (1H, br, OH), 3.96 (3H, s, CH_3_), 3.27 (3H, s, CH_3_). ^13^C-NMR (125 MHz, CDCl_3_+MeOD) δ 166.7, 153.8, 151.9, 146.3, 137.6, 129.4, 128.2, 123.0, 121.7, 118.3, 118.1, 117.5, 115.6, 114.9, 108.3, 59.6, 25.5. LCMS (ESI^+^) *m/z* calcd. for C_17_H_13_NO_4_ ([M + H^+^]) 295.3, found 295.9

*9-(2-(Diethylamino)ethoxy)-5-methoxy-1-methylbenzo[6,7]oxepino[4,3,2-cd]isoindol-2(1H)-one* (**8be**). Rf 0.22 (hexane/EtOAc = 6/4). Yield = 34% (212 mg). Light orange solid. mp: 174–176 °C; ^1^H-NMR (CDCl_3_, 300 MHz) δ 7.37 (1H, d, *J* = 8.1 Hz, *Ar*), 6.96 (2H, t, *J* = 6.5 Hz, *Ar*), 6.65 (1H, dd, *J* = 3.0 Hz, 8.79 Hz, *Ar*), 6.56–6.55 (1H, m, *Ar*), 5.74 (1H, s, *Ar*), 4.15 (2H, t, *J* = 6.1 Hz, CH_2_), 3.95 (3H, s, CH_3_), 3.26 (3H, s, CH_3_), 2.90 (2H, t, *J* = 6.0 Hz, CH_2_), 2.67 (4H, q, *J* = 7.1 Hz, 2×CH_2_), 1.08 (6H, t, *J* = 7.1 Hz, 2×CH_3_). ^13^C-NMR (125 MHz, CDCl_3_) δ 166.1, 155.7, 151.7, 147.1, 142.1, 137.9, 128.4, 126.9, 122.9, 121.8, 118.2, 117.0, 114.8, 114.3, 107.3, 66.5, 56.6, 51.5, 47.6, 25.5, 11.5. LCMS (ESI^+^) *m/z* calcd. for C_23_H_27_N_2_O_4_ ([M + H^+^]) 394.5, found 395.0

*5-Methoxy-1-methyl-9-(pyridine-2-ylmethoxy)benzo[6,7]oxepino[4,3,2-cd]isoindol-2(1H)-one* (**8bf**). Rf 0.24 (DCM/EtOAc = 9/1). Yield = 39% (231 mg). Light yellow solid. mp: 176–178 °C; ^1^H-NMR (CDCl_3_, 300 MHz) δ 8.58 (1H, dd, *J* = 5.8 Hz, 1.5 Hz, *Ar*), 7.72 (1H, td, *J* = 7.7 Hz, 1.7 Hz, *Ar*), 7.48 (1H, d, *J* = 7.8 Hz, *Ar*), 7.24–7.20 (2H, m, *Ar*), 6.92–6.85 (2H, m, *Ar*), 6.70(1H, dd, *J* = 3.0 Hz, 8.7 Hz, *Ar*), 6.56 (1H, d, *J* = 3.0 Hz, *Ar*), 5.63 (1H, s, *Ar*), 5.12 (2H, s, CH_2_), 3.88 (3H, s, CH_3_), 3.18 (3H, s, CH_3_). ^13^C-NMR (125 MHz, CDCl_3_) δ 166.1, 156.9, 155.3, 151.8, 149.1, 147.4, 142.0, 138.0, 136.8, 128.6, 126.9, 123.0, 122.7, 121.8, 121.3, 118.3, 117.2, 114.9, 114.6, 107.2, 70.8, 56.6, 25.6. LCMS (ESI^+^) *m/z* calcd. for C_23_H_19_N_2_O_4_ ([M + H^+^]) 386.4, found 386.9

*9-Benzyloxy-5-methoxy-1-methylbenzo[6,7]oxepino[4,3,2-cd]isoindol-2(1H)-one* (**8bg**). Rf 0.26 (hexane/EtOAc = 6/4). Yield = 44% (265 mg). Yellow solid. mp: 187–189 °C; ^1^H-NMR (CDCl_3_, 300 MHz) δ 7.41–7.33 (6H, m, *Ar*), 6.96–6.93 (2H, m, *Ar*), 6.74 (1H, dd, *J* = 8.7 Hz, 3.00 Hz, *Ar*), 6.61 (1H, d, *J* = 3.0 Hz, *Ar*), 5.72 (1H, s, *Ar*), 5.02 (2H, s, CH_2_), 3.93 (3H, s, CH_3_), 3.25 (3H, s, CH_3_). ^13^C-NMR (125 MHz, CDCl_3_) δ 166.2, 155.8, 151.9, 147.3, 142.2, 138.1, 136.7, 128.6, 128.6, 128.0, 127.4, 127.1, 123.0, 121.9, 118.5, 117.4, 114.9, 114.7, 107.4, 70.3, 56.7, 25.6. LCMS (ESI^+^) *m/z* calcd. for C_24_H_20_NO_4_ ([M + H^+^]) 385.4, found 385.9

*5-Methoxy-1-methyl-[1,3]dioxolo[4”,5”.4′,5′]benzo[1′,2′:6,7]oxepino[4,3,2-cd]isoindol-2(1H)-one* (**8bh**). Rf 0.24 (hexane/EtOAc = 6/4). Yield = 28% (140 mg). Bright orange solid. mp: 237–239 °C; ^1^H-NMR (CDCl_3_, 300 MHz) δ 7.32 (1H, d, *J* = 8.2 Hz, *Ar*), 6.93 (1H, d, *J* = 8.2 Hz, *Ar*), 6.59 (1H, s, *Ar*), 6.42 (1H, s, *Ar*), 5.93 (2H, s, CH_2_), 5.66 (1H, s, *Ar*), 3.90 (3H, s, CH_3_), 3.20 (3H, s, CH_3_). ^13^C-NMR (125 MHz, CDCl_3_) δ 166.1, 151.8, 148.5, 148.1, 144.9, 136.4, 128.0, 121.7, 120.8, 118.9, 115.0, 109.4, 107.7, 104.4, 101.9, 56.6, 25.6. LCMS (ESI^+^) *m/z* calc’d for C_18_H_14_NO_5_ ([M + H^+^]) 323.3, found 323.9

*9-Isobutoxy-5-ethoxy-1-methylbenzo[6,7]oxepino[4,3,2-cd]isoindol-2(1H)-one* (**8bi**). Rf 0.22 (hexane/EtOAc = 6/4). Yield = 61% (323 mg). Light organe solid. mp: 178–180 °C; ^1^H-NMR (CDCl_3_, 300 MHz) δ 7.32 (1H, d, *J* = 8.1 Hz, *Ar*), 6.95–6.92 (2H, m, *Ar*), 6.66 (1H, dd, *J* = 8.7 Hz, *J* = 2.9 Hz, *Ar*), 6.54 (1H, d, *J* = 2.8 Hz, *Ar*), 5.74 (1H, s, *Ar*), 4.16 (2H, q, *J* = 6.9 Hz, CH_2_), 3.68 (2H, d, *J* = 6.5 Hz, CH_2_), 3.24 (3H, s, CH_3_), 2.09–2.01 (1H, m, CH), 1.49 (3H, t, *J* = 6.9 Hz, CH_3_), 1.01 (6H, d, *J* = 6.7 Hz, 2×CH_3_). ^13^C-NMR (126 MHz, CDCl_3_) δ 166.2, 156.3, 151.3, 147.0, 142.5, 138.0, 128.5, 127.2, 122.9, 121.9, 118.3, 116.9, 116.6, 114.4, 107.4, 74.8, 65.4, 28.2, 25.6, 19.2, 14.8. LCMS (ESI^+^) *m/z* calc’d for C_22_H_24_NO_4_ ([M + H^+^]) 365.4, found 366.0

*5-Ethoxy-1-methyl-9-(trifluoromethyl)benzo[6,7]oxepino[4,3,2-cd]isoindol-2(1H)-one* (**8bj**). Rf 0.25 (hexane/EtOAc = 6/4). Yield = 72% (370 mg). Yellow solid. mp: 230–232 °C; ^1^H-NMR (CDCl_3_, 300 MHz) δ 7.34–26 (2H, m, *Ar*), 7.19 (1H, m, *Ar*), 7.05 (1H, d, *J* = 8.4 Hz, *Ar*), 6.94 (1H, d, *J* = 8.2 Hz, *Ar*), 5.69 (1H, s, *Ar*), 4.14 (2H, q, *J* = 6.9 Hz, CH_2_), 3.22 (3H, s, CH_3_), 1.49 (3H, t, *J* = 6.9 Hz, CH_3_). ^13^C-NMR (125 MHz, CDCl_3_) δ 166.1, 155.9, 151.3, 141.5, 138.7, 128.3, 128.2, 127.5, 126.7, 126.1, 124.7, 122.7, 122.5, 121.7, 119.0, 116.5, 65.3, 25.6, 14.8. LCMS (ESI^+^) *m/z* calcd. for C_19_H_15_F_3_NO_3_ ([M + H^+^]) 361.3, found 361.9

*5-Ethoxy-9-methoxy-1-methylbenzo[6,7]oxepino[4,3,2-cd]isoindol-2(1H)-one* (**8bk**). Rf 0.22 (hexane/EtOAc = 6/4). Yield = 70% (325 mg). Light yellow solid. mp: 214–216 °C; ^1^H-NMR (CDCl_3_, 300 MHz) δ 7.35 (1H, d, *J* = 8.1 Hz, *Ar*), 6.99–6.94 (2H, m, *Ar*), 6.70 (1H, dd, *J* = 8.8 Hz, 3.03 Hz, *Ar*), 6.55 (1H, d, *J* = 3.0 Hz, *Ar*), 5.77 (1H, s, *Ar*), 4.15 (2H, q, *J* = 7.0 Hz, CH_2_), 3.80 (3H, s, CH_3_), 3.27 (3H, s, CH_3_), 1.52 (3H, t, *J* = 6.9 Hz, CH_3_). ^13^C-NMR (125 MHz, CDCl_3_) δ 166.2. 156.5. 151.3. 147.1. 142.4. 138.1. 128.5. 127.1. 123.0. 121.8. 118.3. 116.5. 116.2. 113.6. 107.3. 65.4. 55.5. 25.5. 14.8. LCMS (ESI^+^) *m/z* calcd. for C_19_H_18_NO_4_ ([M + H^+^]) 323.3, found 323.9

*5-Ethoxy-9-hydroxy-1-methylbenzo[6,7]oxepino[4,3,2-cd]isoindol-2(1H)-one* (**8bl**). Rf 0.24 (hexane/EtOAc = 6/4). Yield = 31% (72 mg). Yellow solid. mp: 194–196 °C; ^1^H-NMR (CDCl_3_+MeOD, 300 MHz) δ 7.26 (1H, d, *J* = 8.1 Hz, *Ar*), 6.91 (1H, d, *J* = 8.2 Hz, *Ar*), 6.84 (1H, d, *J* = 8.6 Hz, *Ar*), 6.58 (1H, dd, *J* = 8.6 Hz, *J* = 2.8 Hz, *Ar*), 6.43 (1H, d, *J* = 2.7 Hz, *Ar*), 5.71 (1H, s, *Ar*), 4.14 (2H, q, *J* = 6.9 Hz, CH_2_), 3.19 (3H, s, CH_3_), 1.47 (3H, t, *J* = 6.9 Hz, CH_3_). ^13^C-NMR (125 MHz, CDCl_3_+MeOD) δ 166.8, 153.8, 151.3, 146.3, 142.5, 137.5, 128.2, 127.2, 122.9, 121.5, 118.2, 117.4, 116.6, 115.6, 108.4, 65.5, 25.5, 14.6. LCMS (ESI^+^) *m/z* calcd. for C_18_H_16_NO_4_ ([M + H^+^]) 309.3, found 310.0

*9-(2-(Diethylamino)ethoxy)-5-ethoxy-1-methylbenzo[6,7]oxepino[4,3,2-cd]isoindol-2(1H)-one* (**8bm**). Rf 0.18 (DCM/MeOH = 19/1). Yield = 34% (393 mg). Light yellow solid. mp: 169–171 °C; ^1^H-NMR (CDCl_3_, 300 MHz) δ 7.34 (1H, d, *J* = 8.1 Hz, *Ar*), 6.97–6.94 (2H, m, *Ar*), 6.69 (1H, dd, *J* = 3.0 Hz, 8.82 Hz, *Ar*), 6.56 (1H, d, *J* = 3.0 Hz, *Ar*), 5.74 (1H, s, *Ar*), 4.20 (2H, q, *J* = 6.9 Hz, CH_2_), 4.07 (2H, t, *J* = 6.1 Hz, CH_2_), 3.26 (3H, s, CH_3_), 2.93 (2H, t, *J* = 6.0 Hz, CH_2_), 2.73 (4H, q, *J* = 7.1 Hz, 2×CH_2_), 1.53 (3H, t, *J* = 6.9 Hz, CH_3_), 1.14 (6H, t, *J* = 7.1 Hz, 2×CH_3_). ^13^C-NMR (125 MHz, CDCl_3_) δ 166.2, 155.7, 151.3, 147.2, 142.4, 138.0, 128.5, 127.1, 122.9, 121.8, 118.3, 116.9, 116.5, 114.3, 107.3, 66.4, 65.4, 51.5, 47.6, 25.5, 14.8, 11.4. LCMS (ESI^+^) *m/z* calcd. for C_24_H_29_N_2_O_4_ ([M + H^+^]) 408.5, found 409.0

*5-Ethoxy-1-methyl-9-(pyridine-2-ylmethoxy)benzo[6,7]oxepino[4,3,2-cd]isoindol-2(1H)-one* (**8bn**). Rf 0.23 (DCM/EtOAc = 6/1). Yield = 52% (307 mg). Light yellow solid. mp: 188–190 °C; ^1^H-NMR (CDCl_3_, 300 MHz) δ 8.54–8.52 (1H, m, *Ar*), 7.66–7.63 (1H, m, *Ar*), 7.45–7.28 (1H, m, *Ar*), 7.21–7.15 (2H, m, *Ar*), 6.88–6.81 (2H, m, *Ar*), 6.68 (1H, dd, *J* = 8.7 Hz, 3.0 Hz, *Ar*), 6.53 (1H, d, *J* = 3.0 Hz, *Ar*), 5.59 (1H, s, *Ar*), 5.08 (2H, s, CH_2_), 4.08 (2H, q, *J* = 6.9 Hz, CH_2_), 3.14 (3H, s, CH_3_), 1.45 (3H, t, *J* = 6.9 Hz, CH_3_). ^13^C-NMR (125 MHz, CDCl_3_) δ 166.1, 156.8, 155.2, 151.2, 149.0, 147.4, 142.2, 138.0, 136.8, 129.4, 128.6, 123.0, 122.6, 121.2, 119.4, 118.3, 117.2, 116.4, 114.6, 107.2, 70.7, 65.3, 25.5, 14.8. LCMS (ESI^+^) *m/z* calcd. for C_24_H_21_N_2_O_4_ ([M + H^+^]) 400.4, found 400.9

*9-Benzyloxy-5-methoxy-1-methylbenzo[6,7]oxepino[4,3,2-cd]isoindol-2(1H)-one* (**8bo**). Rf 0.27 (hexane/EtOAc = 6/4). Yield = 44% (391 mg). Orange solid. mp: 198–200 °C; ^1^H-NMR (CDCl_3_ 300 MHz) δ 7.41–7.30 (6H, m, *Ar*), 6.96–6.92 (2H, m, *Ar*), 6.74 (1H, dd, *J* = 8.8 Hz, 3.00 Hz, *Ar*), 6.62 (1H, d, *J* = 3.0 Hz, *Ar*), 5.73 (1H, s, *Ar*), 5.02 (2H, s, CH_2_), 4.13 (2H, q, *J* = 6.9 Hz, CH_2_), 3.24 (3H, s, CH_3_), 1.49 (3H, t, *J* = 6.9 Hz, CH_3_). ^13^C-NMR (125 MHz, CDCl_3_) δ 166.3, 155.8, 151.3, 147.4, 142.5, 138.2, 136.7, 128.7, 128.6, 128.0, 127.4, 127.3, 123.1, 121.9, 118.4, 117.3, 116.6, 114.7, 107.2, 70.3, 65.5, 25.6, 14.9. LCMS (ESI^+^) *m/z* calcd. for C_25_H_22_NO_4_ ([M + H^+^]) 399.4, found 399.9

*5-Ethoxy-1-methyl-[1,3]dioxolo[4”,5”.4′,5′]benzo[1′,2′:6,7]oxepino[4,3,2-cd]isoindol-2(1H)-one* (**8bp**). Rf 0.23 (hexane/EtOAc = 6/4). Yield = 47% (238 mg). Bright orange solid. mp: 207–209 °C; ^1^H-NMR (CDCl_3_, 300 MHz) δ 7.32 (1H, d, *J* = 8.1 Hz, *Ar*), 6.93 (1H, d, *J* = 8.2 Hz, *Ar*), 6.59 (1H, s, *Ar*), 6.43 (1H, s, *Ar*), 5.95 (2H, s, CH_2_), 5.65 (1H, s, *Ar*), 4.11 (2H, q, *J* = 6.9 Hz, CH_2_), 3.90 (3H, s, CH_3_), 1.48 (3H, t, *J* = 6.9 Hz, CH_3_). ^13^C-NMR (125 MHz, CDCl_3_) δ 166.0, 151.2, 148.3, 148.0, 144.8, 141.5, 136.4, 128.0, 121.6, 120.8, 118.7, 116.4, 109.4, 107.5, 104.3, 101.9, 65.3, 25.5, 14.8. LCMS (ESI^+^) *m/z* calcd. for C_19_H_16_NO_5_ ([M + H^+^]) 337.3, found 337.9

*5-(Diethylamino)-9-isobutoxy-1-methyl-5-propylbenzo[6,7]oxepino[4,3,2-cd]isoindol-2(1H)-one* (**8ca**). Rf 0.27 (DCM/EtOAc = 6/1). Yield = 32% (164 mg). Light yellow solid. mp: 183–185 °C; ^1^H-NMR (CDCl_3_, 300 MHz) δ 6.81 (1H, d, *J* = 8.7 Hz, *Ar*), 6.58–6.54 (2H, m, *Ar*), 6.47 (1H, d, *J* = 2.8 Hz, *Ar*), 6.17 (1H, d, *J* = 1.8 Hz, *Ar*), 5.48 (1H, s, *Ar*), 3.66 (2H, d, *J* = 6.5 Hz, CH_2_), 3.37 (4H, q, *J* = 7.0 Hz, 2×CH_2_), 3.21 (3H, s, CH_3_), 2.04 (1H, m, CH), 1.20 (6H, t, *J* = 7.0 Hz, 2×CH_3_), 1.02 (6H, d, *J* = 6.7 Hz, 2×CH_3_). ^13^C-NMR (125 MHz, CDCl_3_) δ 167.5, 155.9, 154.8, 151.8, 147.1, 138.4, 131.5, 128.4, 122.5, 117.0, 113.6, 103.8, 102.9, 99.6, 44.8, 28.2, 25.5, 19.2, 12.6. LCMS (ESI^+^) *m/z* calcd. for C_24_H_29_N_2_O_3_ ([M + H^+^]) 392.5, found 393.0

*5-(Diethylamino)-9-hydroxy-1-methylbenzo[6,7]oxepino[4,3,2-cd]isoindol-2(1H)-one* (**8cb**). Rf 0.22 (DCM/EtOAc = 6/1). Yield = 91% (78 mg). Yellow solid. mp: 181–183 °C; ^1^H-NMR (CDCl_3_ 300 MHz) δ 7.38 (1H, d, *J* = 8.3 Hz, *Ar*), 6.95–6.89 (2H, m, *Ar*), 6.58 (1H, dd, *J* = 8.5 Hz, 2.94 Hz, *Ar*), 5.73 (1H, s, *Ar*), 4.79 (1H, br, OH), 3.96 (3H, s, CH_3_), 3.27 (3H, s, CH_3_). ^13^C-NMR (125 MHz, CDCl_3_+MeOD) δ 167.7, 156.7, 154.8, 153.1, 146.7, 142.4, 138.0, 131.4, 129.6, 122.7, 118.4, 117.5, 115.0, 114.6, 113.7, 64.8, 25.6, 12.3. LCMS (ESI^+^) *m/z* calcd. for C_20_H_21_N_2_O_3_ ([M + H^+^]) 336.4, found 337.0

*5-(Diethylamino)-9-hydroxy-1-methyl-11,11a-dihydrobenzo[6,7]oxepino[4,3,2-cd]isoindol-2(1H)-one* (**8cc**). Rf 0.19 (DCM/EtOAc = 6/1). Yield = 38% (90 mg). Light yellow solid. mp: 165–167 °C; ^1^H-NMR (CDCl_3_, 300 MHz) δ 7.12 (1H, d, *J* = 8.7 Hz, *Ar*), 6.86–6.81 (2H, m, *Ar*), 6.78 (1H, d, *J* = 2.7 Hz, *Ar*), 6.64 (1H, br, OH), 6.52 (1H, d, *J* = 1.8 Hz, *Ar*), 4.44 (1H, dd, *J* = 11.2 Hz, 2.1 Hz, CH), 3.43 (4H, q, *J* = 7.0 Hz, 2×CH_2_), 3.30 (1H, dd, *J* = 2.3 Hz, 13.1 Hz, CH), 3.18 (3H, s, CH_3_), 2.89 (1H, t, *J* = 11.9 Hz, CH), 1.19 (6H, t, *J* = 6.9 Hz, 2×CH_3_). ^13^C-NMR (125 MHz, CDCl_3_) δ 169.4, 152.0, 151.4, 149.8, 148.0, 133.7, 124.9, 122.4, 118.2, 118.1, 115.5, 103.3, 100.5, 60.1, 44.7, 38.2, 27.4, 12.4. LCMS (ESI^+^) *m/z* calcd. for C_20_H_23_N_2_O_3_ ([M + H^+^]) 338.4, found 339.0

*9-(Benzyloxy)-5-(diethylamino)-1-methylbenzo[6,7]oxepino[4,3,2-cd]isoindol-2(1H)-one* (**8cd**). Rf 0.29 (DCM/EtOAc = 6/1). Yield = 42% (233 mg). Yellow solid. mp: 185–188 °C; ^1^H-NMR (CDCl_3_, 300 MHz) δ 7.45–7.37 (5H, m, *Ar*), 6.87 (1H, d, *J* = 8.7 Hz, *Ar*), 6.69 (1H, dd, *J* = 8.8 Hz, 3.00 Hz, *Ar*), 6.61–6.59 (2H, m, *Ar*), 6.21 (1H, d, *J* = 2.0 Hz, *Ar*), 5.52 (1H, s, *Ar*), 5.04 (2H, s, CH_2_), 3.39 (4H, q, *J* = 7.0 Hz, 2×CH_2_), 3.25 (3H, s, CH_3_), 1.21 (6H, t, *J* = 7.0 Hz, 2×CH_3_). ^13^C-NMR (125 MHz, CDCl_3_) δ 167.5, 155.4, 154.8, 151.9, 147.5, 138.6, 136.8, 131.6, 128.7, 128.6, 128.0, 127.4, 122.6, 117.3, 113.9, 112.4, 103.5, 102.9, 99.7, 70.3, 44.8, 25.5, 12.6. LCMS (ESI^+^) *m/z* calcd. for C_27_H_27_N_2_O_3_ ([M + H^+^]) 426.5, found 427.9

*5-(Diethylamino)-1-methyl-[1,3]dioxolo[4”,5”.4′,5′]benzo[1′,2′:6,7]oxepino[4,3,2-cd]isoindol-2(1H)-one* (**8ce**). Rf 0.24 (DCM/EtOAc = 6/1). Yield = 21% (96 mg). Bright yellow solid. mp: 199–201 °C; ^1^H-NMR (CDCl_3_, 300 MHz) δ 6.58 (1H, s, *Ar*), 6.48 (1H, s, *Ar*), 6.40 (1H, s, *Ar*), 6.16 (1H, s, *Ar*), 5.96–5.93 (2H, m, CH_2_), 5.42 (1H, s, Ar), 3.39 (4H, q, *J* = 6.8 Hz, 2×CH_2_), 3.21 (3H, s, CH_3_), 1.19 (6H, t, *J* = 6.9 Hz, 2×CH_3_). ^13^C-NMR (125 MHz, CDCl_3_) δ 167.3, 154.3, 151.8, 148.1, 147.6, 144.3, 136.8, 131.3, 120.6, 113.0, 109.6, 104.0, 103.8, 102.9, 101.7, 99.7, 44.8, 25.5, 12.5. LCMS (ESI^+^) *m/z* calcd. for C_21_H_21_N_2_O_4_ ([M + H^+^]) 364.4, found 365.9

*9-Isobutoxy-1-methyl-5-propylbenzo[6,7]oxepino[4,3,2-cd]isoindol-2(1H)-one* (**8da**). Rf 0.27 (hexane/EtOAc = 6/4). Yield = 21% (63 mg). Yellow solid. mp: 196–199 °C; ^1^H-NMR (CDCl_3_, 300 MHz) δ 7.30 (1H, d, *J* = 7.5 Hz, *Ar*), 7.21 (1H, d, *J* = 7.5 Hz, *Ar*), 6.89 (1H, d, *J* = 8.7 Hz, *Ar*), 6.69 (1H, dd, *J* = 8.7 Hz, 3.00 Hz, *Ar*), 6.56 (1H, d, *J* = 2.9 Hz, *Ar*). 5.75 (1H, s, *Ar*), 3.70 (2H, d, *J* = 6.5 Hz, CH_2_), 3.27 (3H, s, CH_3_), 2.71 (2H, t, *J* = 7.2 Hz, CH_2_), 2.10–2.05 (1H, m, CH), 1.72–1.65 (2H, m, CH_2_), 1.05 (3H, s, CH_3_), 1.03 (3H, s, CH_3_), 1.02–0.99 (3H, m, CH_3_). ^13^C-NMR (125 MHz, CDCl_3_) δ 166.5, 156.2, 151.3, 147.1, 138.2, 136.0, 133.2, 128.2, 126.1, 122.6, 117.4, 117.0, 114.4, 107.4, 74.8, 32.3, 28.2, 25.6, 23.6, 19.2, 14.0. LCMS (ESI^+^) *m/z* calcd. for C_23_H_26_NO_3_ ([M + H^+^]) 363.4, found 365.0

*1-Methyl-5-propyl-9-(trifluoromethyl)benzo[6,7]oxepino[4,3,2-cd]isoindol-2(1H)-one* (**8db**). Rf 0.25 (hexane/EtOAc = 6/4). Yield = 19% (100 mg). Light yellow solid. mp: 239–241 °C; ^1^H-NMR (CDCl_3_, 300 MHz) δ 7.36–7.29 (2H, m, *Ar*), 7.24–7.20 (2H, m, *Ar*), 6.99 (1H, d, *J* = 8.3 Hz, *Ar*), 5.74 (1H, s, *Ar*), 3.26 (3H, s, CH_3_), 2.69 (2H, t, *J* = 7.3 Hz, CH_2_), 1.71–1.63 (2H, m, CH_2_), 1.01 (3H, t, *J* = 7.3 Hz, CH_3_). ^13^C-NMR (125 MHz, CDCl_3_) δ 166.3, 156.0, 150.3, 138.8, 136.1, 133.8, 128.4, 128.3, 128.2, 127.7, 126.1, 125.7, 124.7, 122.3, 118.0, 106.1, 32.3, 25.6, 23.5, 13.9. LCMS (ESI^+^) *m/z* calcd. for C_20_H_17_F_3_NO_2_ ([M + H^+^]) 359.3, found 359.9

*1-Methyl-5-propyl-9-(pyridine-2-ylmethoxy)benzo[6,7]oxepino[4,3,2-cd]isoindol-2(1H)-one* (**8dc**). Rf 0.26 (hexane/EtOAc = 6/4). Yield = 9.3% (54 mg). Light yellow solid. mp: 189–191 °C; ^1^H-NMR (CDCl_3_, 300 MHz) δ 8.61 (1H, d, *J* = 4.5 Hz, *Ar*), 7.72 (1H, td, *J* = 7.7 Hz, 1.62 Hz, *Ar*), 7.47 (1H, d, *J* = 7.7 Hz, *Ar*), 7.28–7.16 (3H, m, *Ar*), 6.88 (1H, d, *J* = 8.7 Hz, *Ar*), 6.74 (1H, dd, *J* = 8.7 Hz, 3.0 Hz, *Ar*), 6.64 (1H, d, *J* = 2.9 Hz, *Ar*), 5.71 (1H, s, *Ar*), 5.16 (2H, s, CH_2_), 3.25 (3H, s, CH_3_), 2.68 (2H, t, *J* = 7.3 Hz, CH_2_), 1.69–1.62 (2H, m, CH_2_), 0.99 (3H, t, *J* = 7.3 Hz, CH_3_). ^13^C-NMR (125 MHz, CDCl_3_) δ 166.5, 156.9, 155.3, 151.1, 149.2, 147.6, 138.4, 136.8, 136.0, 133.3, 128.7, 128.2, 126.1, 122.8, 122.7, 121.2, 117.4, 117.3, 114.7, 107.1, 70.9, 32.3, 25.6, 23.6, 14.0. LCMS (ESI^+^) *m/z* calcd. for C_25_H_23_N_2_O_3_ ([M + H^+^]) 398.5, found 398.9

*9-(Benzyloxy)-1-methyl-5-propylbenzo[6,7]oxepino[4,3,2-cd]isoindol-2(1H)-one* (**8dd**). Rf 0.21 (hexane/EtOAc = 6/4). Yield = 21% (38 mg). Yellow solid. mp: 203–205 °C; ^1^H-NMR (CDCl_3_, 300 MHz) δ 7.44–7.36 (5H, m, *Ar*), 7.31 (1H, d, *J* = 7.6 Hz, *Ar*), 7.22 (1H, d, *J* = 7.6 Hz, *Ar*), 6.91 (1H, d, *J* = 8.7 Hz, *Ar*), 6.76 (1H, dd, *J* = 8.7 Hz, 2.97 Hz, *Ar*), 6.65 (1H, d, *J* = 2.9 Hz, *Ar*), 5.74 (1H, s, *Ar*), 5.05 (2H, s, CH_2_), 3.27 (3H, s, CH_3_), 2.71 (2H, t, *J* = 7.4 Hz, CH_2_), 1.72–1.65 (2H, m, CH_2_), 1.02 (3H, t, *J* = 7.3 Hz, CH_3_). ^13^C-NMR (125 MHz, CDCl_3_) δ 166.5, 155.7, 151.2, 147.5, 138.3, 136.7, 136.0, 133.3, 128.7, 128.6, 128.2, 128.0, 127.4, 126.1, 122.7, 117.4, 114.8, 107.2, 70.4, 32.3, 25.6, 23.7, 14.0. LCMS (ESI^+^) *m/z* calcd. for C_26_H_24_NO_3_ ([M + H^+^]) 397.4, found 397.9

*1-methyl-5-propyl-[1,3]dioxolo[4”,5”.4′,5′]benzo[1′,2′:6,7]oxepino[4,3,2-cd]isoindol-2(1H)-one* (**8de**). Rf 0.23 (hexane/EtOAc = 6/4). Yield = 19% (93 mg). Light yellow solid. mp: 257–259 °C; ^1^H-NMR (CDCl_3_, 300 MHz) δ 7.31 (1H, d, *J* = 7.6 Hz, *Ar*), 7.20 (1H, d, *J* = 7.6 Hz, *Ar*), 6.53 (1H, s, *Ar*), 6.47 (1H, s, *Ar*), 5.96 (2H, s, CH_2_), 5.68 (2H, s, CH_2_), 3.25 (3H, s, CH_3_), 2.68 (2H, t, *J* = 7.4 Hz, CH_2_), 1.67–1.65 (2H, m, CH_2_), 1.00 (3H, t, *J* = 7.3 Hz, CH_3_). ^13^C-NMR (125 MHz, CDCl_3_) δ 166.3, 150.6, 148.4, 144.7, 136.6, 135.8, 133.2, 128.0, 126.8, 120.8, 120.4, 117.7, 109.5, 107.5, 103.9, 101.9, 32.2, 25.5, 23.7, 14.0. LCMS (ESI^+^) *m/z* calcd. for C_20_H_18_NO_4_ ([M + H^+^]) 335.3, found 335.9

*1,5-Dimethyl-9-(trifluoromethyl)benzo[6,7]oxepino[4,3,2-cd]isoindol-2(1H)-one* (**8df**). Rf 0.27 (hexane/EtOAc = 6/4). Yield = 36% (200 mg). Light yellow solid. mp: 227–229 °C; ^1^H-NMR (CDCl_3_, 300 MHz) δ 7.35 (1H, d, *J* = 8.4 Hz, *Ar*), 7.28–7.19 (3H, m, *Ar*), 7.01 (1H, d, *J* = 8.3 Hz, *Ar*), 5.69 (1H, s, *Ar*), 3.25 (3H, s, CH_3_), 2.34 (3H, s, CH_3_). ^13^C-NMR (125 MHz, CDCl_3_) δ 166.2, 155.9, 150.5, 138.6, 134.3, 131.3, 128.9, 128.5, 127.1, 126.1, 125.3, 124.7, 122.5, 122.4, 117.8, 106.1, 25.5, 16.2. LCMS (ESI^+^) *m/z* calcd. for C_18_H_13_F_3_NO_2_ ([M + H^+^]) 331.3, found 331.9

## 4. Conclusions

In this study, we found that the benzo[6,7]oxepino[4,3,2-*cd*]isoindol-2(1*H*)-one scaffold of aristoyagonine derived from natural products has inhibitory activity against Brd4 bromodomain. Based on the evaluation of inhibitory activities presented in Table 1, Table 2, Table 3 and Table 4, the substituents methoxy, ethoxy, n-propyl, or *N,N*-diethylaminoethox group at R^1^ position showed similar inhibitory activities. These results suggest that R^1^ position is considered to have a tolerance on binding pocket toward Brd4 bromodomain.

Among R^2^ and R^3^ substituents showing inhibitory activity against Brd4 bromodomain, the most potent activity was shown by the smallest atom, hydrogen. The bulky group at R^2^ position perturbed the inhibitory effect. The alkoxy substituents with methoxy, pyridinemethoxy, and *N,N*-diethylaminoethoxy groups at R^4^ position displayed potent activities. On the other hand, introduction of –CF_3_, benzyloxy, or 3,4-dioxymethylene group at R^4^ position showed no activity against Brd4 bromodomain. The hydrogen bonds between C=O of benzo[6,7]oxepino[4,3,2-cd]isoindol-2(1*H*)-one and Asn140 predicted in molecular modeling were confirmed through the results of X-ray co-crystal structure of Brd4 bromodomain and inhibitors (**8ab**, **8bc**, **8bf**, **8be**, and **8bd**). Collectively, our experiment results support these Brd4 bromodomain inhibitors with impressive mode of action profiles. In addition, these novel small molecules are promising advanced lead compounds for further optimization and candidates for the development of epigenetic therapeutics that are used in the treatment of cancer diseases.

## Figures and Tables

**Table 1:** Inhibition effects of compounds fixed with hydrogen at R^4^ position.

Cpds	Structure	Brd4 ^a^ IC_50_(μM)	Cell ^b^ CC_50_(μM)	Cpds	Structure	Brd4 ^a^ IC_50_(μM)	Cell ^b^ CC_50_(μM)
**8aa**	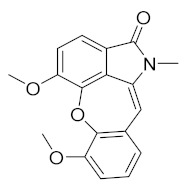	0.84	NT	**8ad**	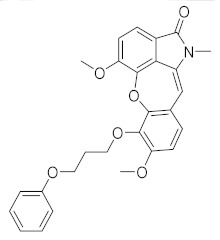	7.07	>4
**8ab**	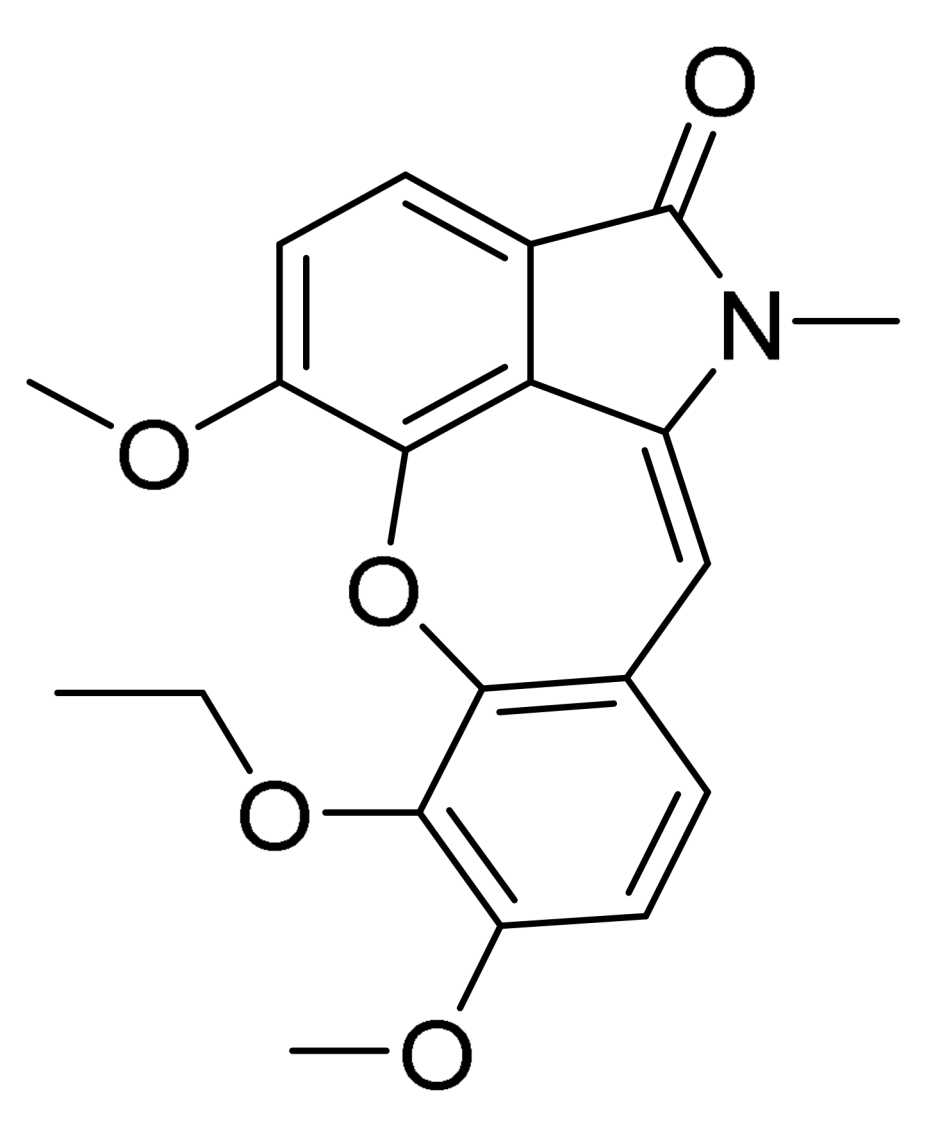	0.86	NT	**8ae**	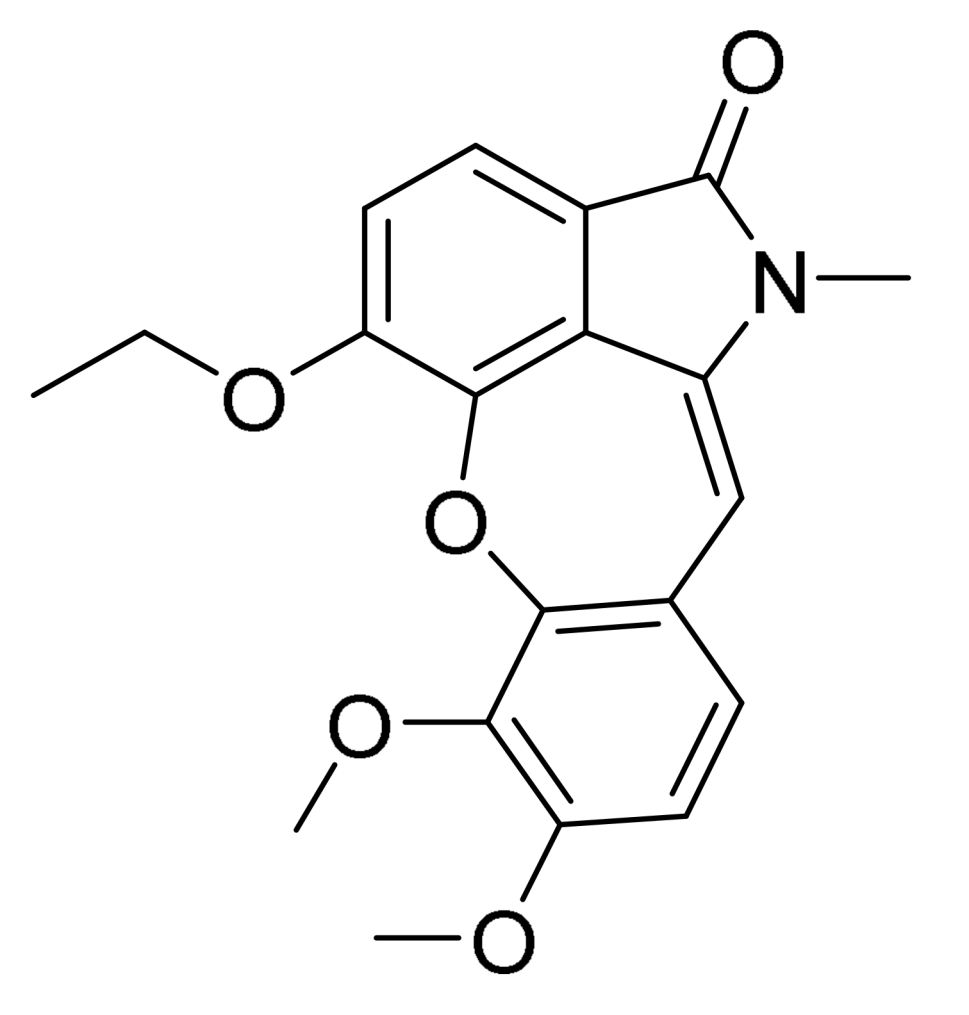	4.16	NT
**8ac**	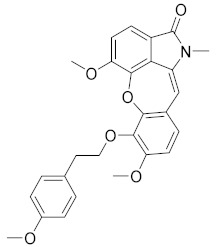	3.74	>20	**8af**	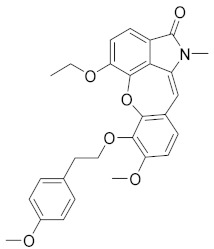	5.33	>4

^a^ To measure the inhibitory effect of compounds against bromodomain binding activity, alpha-screen assay was performed. The IC_50_ in the table was calculated with Prism6 program. ^b^ To measure the Ty82 cell cytotoxic activity of compounds, MTS assay was performed. The CC_50_ in the table was calculated with Prism6 program. NT = not tested.

**Table 2:** Inhibition effects of compounds fixed with hydrogen at R^2^ and R^3^ position.

Cpds	Structure	Brd4 ^a^ IC_50_(μM)	Cell ^b^ CC_50_(μM)	Cpds	Structure	Brd4 ^a^ IC_50_(μM)	Cell ^b^ CC_50_(μM)
**8ba**	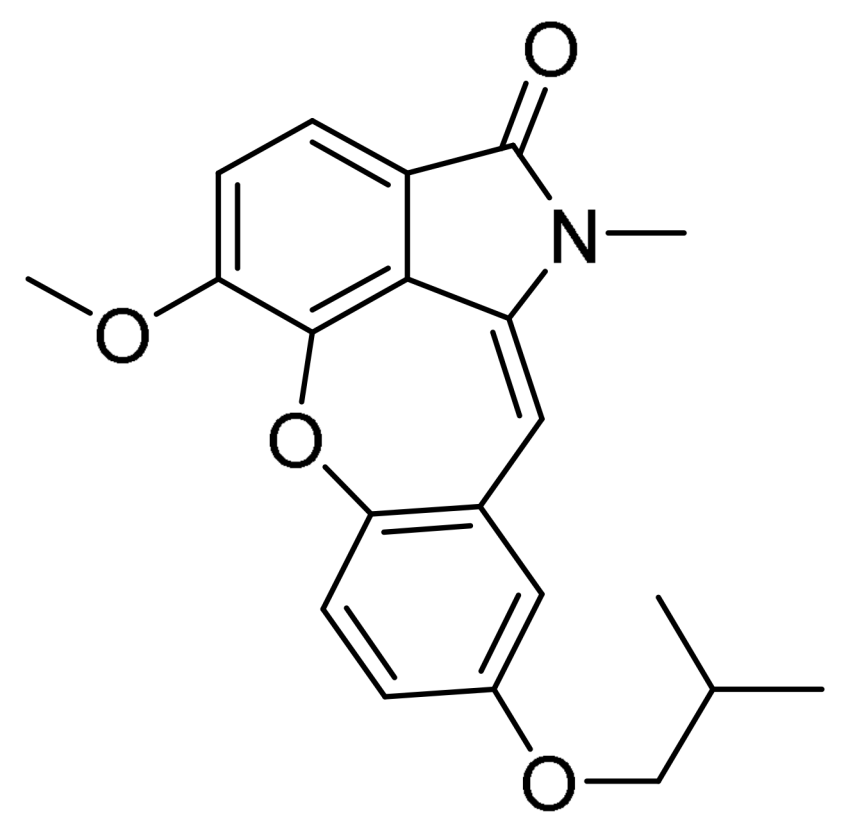	16.7	NT	**8bi**	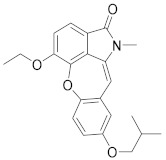	>10	NT
**8bb**	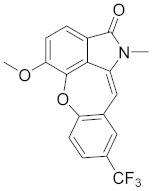	>32	>20	**8bj**	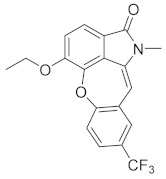	>9	>20
**8bc**	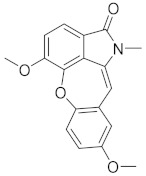	0.41	0.66	**8bk**	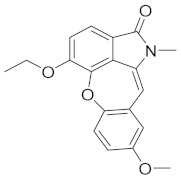	0.86	0.49
**8bd**	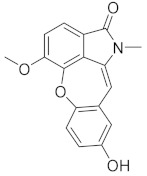	0.41	0.54	**8bl**	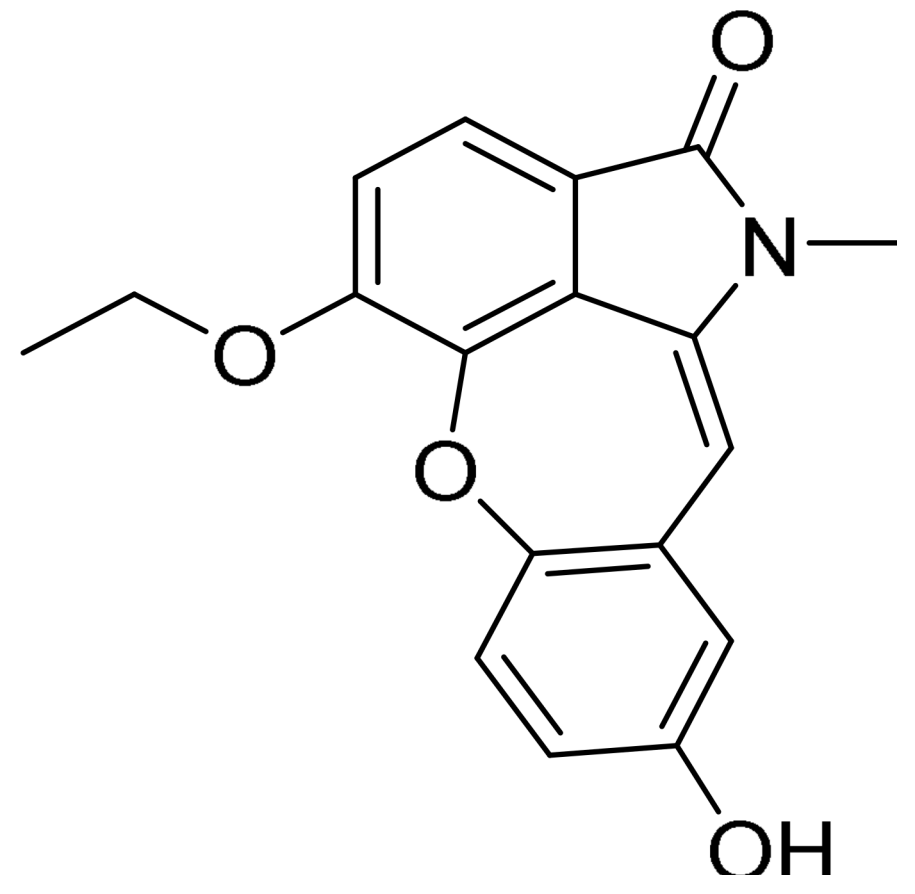	1.12	0.31
**8be**	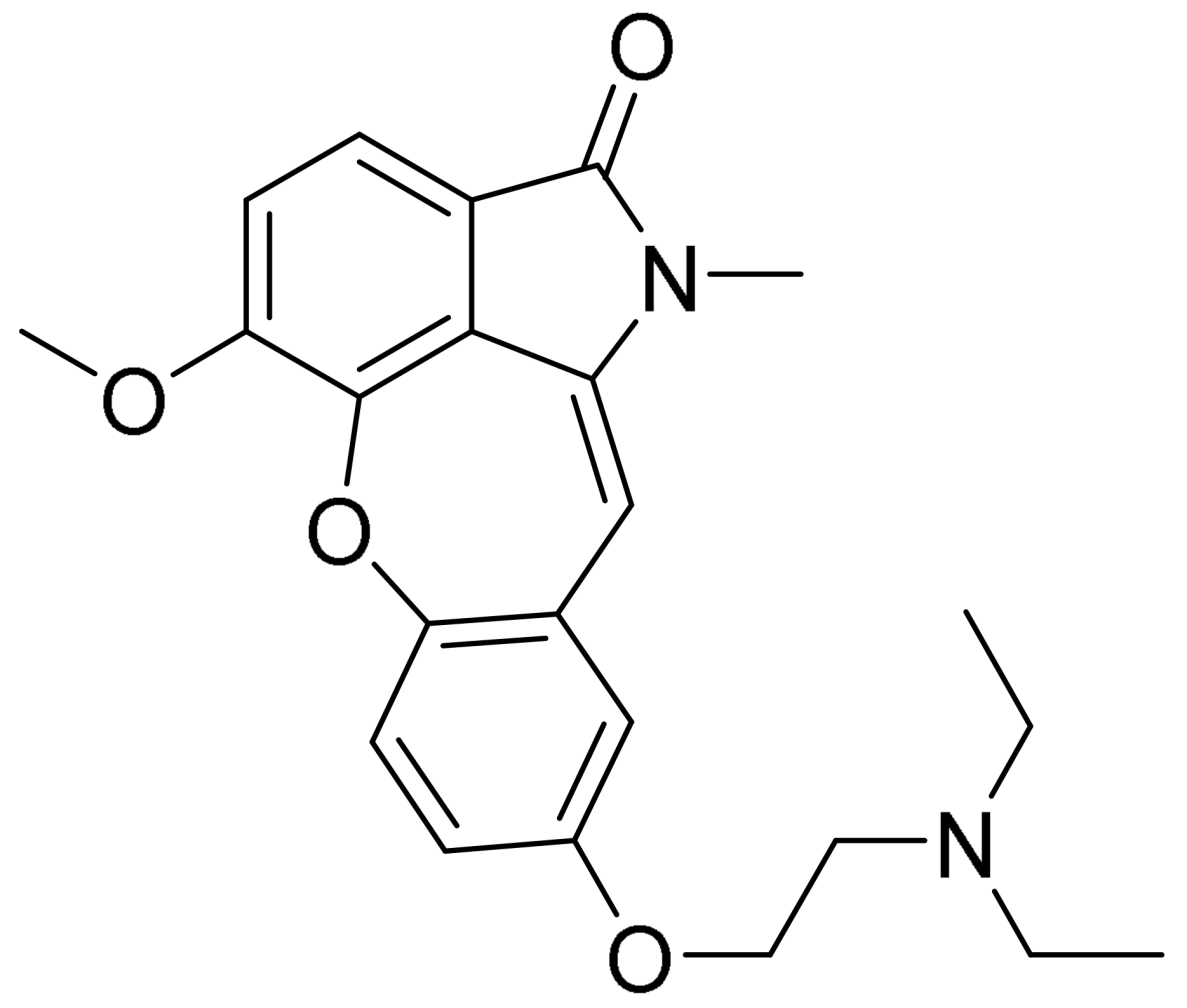	0.86	0.56	**8bm**	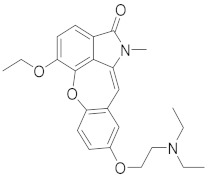	0.91	0.66
**8bf**	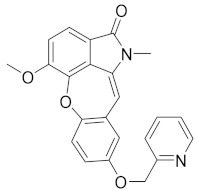	0.91	0.23	**8bn**	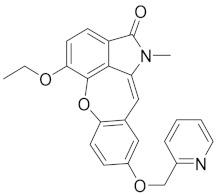	0.68	0.36
**8bg**	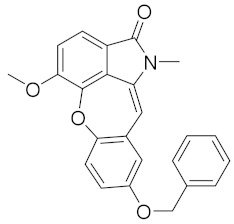	>32	NT	**8bo**	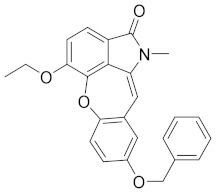	>32	NT
**8bh**	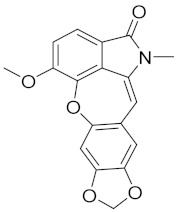	11.1	2.16	**8bp**	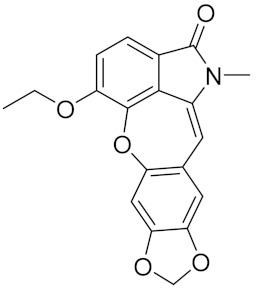	2.89	3.81

^a^ To measure the inhibitory effect of compounds against bromodomain binding activity, alpha-screen assay was performed. The IC_50_ in the table was calculated with Prism6 program. ^b^ To measure the Ty82 cell cytotoxic activity of compounds, MTS assay was performed. The CC_50_ in the table was calculated with Prism6 program. NT = not tested.

**Table 3:** Inhibition effects of compounds fixed with diethylamine at R^1^ position.

Cpds	Structure	Brd4 ^a^ IC_50_(μM)	Cell ^b^ CC_50_(μM)	Cpds	Structure	Brd4 ^a^ IC_50_(μM)	Cell ^b^ CC_50_(μM)
**8ca**	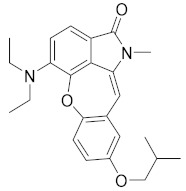	>32	>20	**8cd**	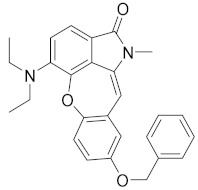	>32	>20
**8cb**	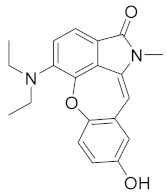	4.89	8	**8ce**	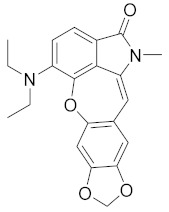	7.29	10
**8cc**	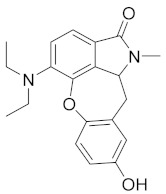	5.86	10				

^a^ To measure the inhibitory effect of compounds against bromodomain binding activity, alpha-screen assay was performed. The IC_50_ in the table was calculated with Prism6 program. ^b^ To measure the Ty82 cell cytotoxic activity of compounds, MTS assay was performed. The CC_50_ in the table was calculated with Prism6 program.

**Table 4:** Inhibition effects of compounds fixed with alkyl chain at R^1^ position.

Cpds	Structure	Brd4 ^a^ IC_50_(μM)	Cell ^b^ IC_50_(μM)	Cpds	Structure	Brd4 ^a^ IC_50_(μM)	Cell ^b^ IC_50_(μM)
**8da**	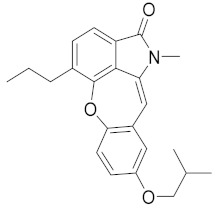	6.64	10	**8dd**	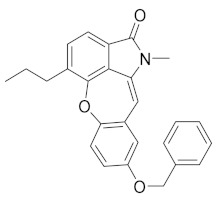	9	10
**8db**	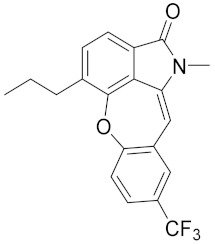	>32	>20	**8de**	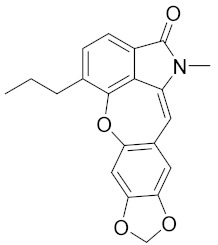	2.69	4.5
**8dc**	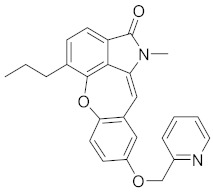	1.37	1.77	**8df**	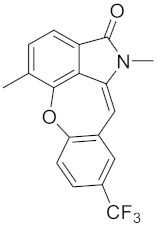	>32	NT

^a^ To measure the inhibitory effect of compounds against bromodomain binding activity, alpha-screen assay was performed. The IC_50_ in the table was calculated with Prism6 program. ^b^ To measure the cell cytotoxic activity of compounds, MTS assay was performed The IC_50_ in the table was calculated with Prism6 program. NT = not tested.

## Data Availability

The structural coordinates and X-ray diffraction data sets were deposited to Protein Data Bank (PDB) under accession code of 6KEC, 6KEH, 6KEI, 6KEJ, and 6KEK, respectively.

## Supplementary Materials

The following are available online, Figure S1: Structure of BRD4 BD1 and 5 compounds analyzed in this study, Table S1: Data collection and refinement statistics, NMR spectra.

## Sample Availability

Samples of the compounds used in this study are available from the authors.

## Abbreviations

Brd4	bromodomain-containing protein 4
BET	bromodomain and extraterminal domain
HAT	Histone acetyltransferase
P-TEFB	Positive transcription elongation factor complex
HMT	Histone methyltransferases
NMC	NUT midline carcinoma
AML	Acute myeloid leukemia
SBDD	structure-based drug design
