# Supplementary material for: Synthesis and Structure–Activity Relationships of Aristoyagonine Derivatives as Brd4 Bromodomain Inhibitors with X-ray Co-Crystal Research
Source: Molecules. 2021 Mar 17;26(6):1686. doi: 10.3390/molecules26061686 (PMC8002823; doi:10.3390/molecules26061686)

Supporting Information

Synthesis and Structure-Activity Relationships of Aristoyagonine Derivatives as Brd4 Bromodomain Inhibitors with X-ray crystal structure

Table of Contents

Table S1: Data collection and refinement statistics -- 2

Figure S1: Structure of BRD4 BD1 and 5 compounds analyzed in this study --------------------------- 3

NMR spectra -- 4

Table S1. Data collection and refinement statistics

Data set	**8ab**	**8bc**	**8bd**	**8be**	**8bf**
**Diffraction data statistics**					
X-ray source	PLS-11C	PLS-11C	PLS-11C	PLS-11C	PLS-11C
Wavelength (Å)	0.979	0.979	0.979	0.979	0.979
Space group	$P2_12_12_1$	$P2_12_12_1$	$P2_12_12_1$	$P2_12_12_1$	$P2_12_12_1$
Cell parameters					
a, b, c (Å)	32.30, 47.53, 79.09	32.40, 47.34, 79.31	32.34, 47.39, 79.42	31.90, 47.21, 79.26	32.08, 47.24, 79.18
α, β, γ (°)	90, 90, 90	90, 90, 90	90, 90, 90	90, 90, 90	90, 90, 90
Resolution (Å)	50.0–1.35 (1.37–1.35)	50.0–1.55 (1.58–1.55)	50.0–1.55 (1.58–1.55)	50.0–1.85 (1.88–1.85)	50.0–1.45 (1.48–1.45)
R_{merge} (%)	5.0 (66.4)	7.2 (64.0)	10.9 (55.7)	8.5 (60.3)	5.0 (64.3)
Mean I/σI	23.6 (2.43)	18.9 (1.75)	13.12 (2.33)	15.25 (3.5)	20.8 (1.67)
Redundancy	6.0 (5)	6.1 (5.1)	5.8 (4.7)	6.0 (5.1)	5.7 (4.2)
Completeness (%)	98.2 (85.2)	99.6 (99.0)	98.4 (94.2)	97.7 (81.6)	95.3 (72.3)
No. of unique reflections	24,812 (1,148)	18,180 (884)	18,038 (835)	10,530 (434)	21,004 (777)
**Refinement statistics**					
Resolution (Å)	40.47–1.35	39.66–1.55	39.71–1.55	39.63–1.85	40.57–1.45
R_{work} /R_{free} (%)	18.1/20.1	19.3/23.0	19.6/23.2	18.9/24.2	18.3/23.1
Protein residues	124	123	123	124	124
No. of nonhydrogen atoms/average B-factor					
Protein	1047/16.38	1031/14.94	1039/15.41	1033/18.87	1033/15.29
Solvent	111/24.60	91/22.40	64/22.79	67/21.31	100/21.92
Ligand	38/21.91	35/19.88	43/21.95	35/23.49	35/22.84
R.m.s. deviation					
Bond lengths (Å)	0.006	0.006	0.007	0.008	0.006
Bond angles (°)	0.83	0.79	0.84	0.91	0.83
Ramachandran plot (%)					
Favored	98.35	98.35	98.35	98.35	98.35
Outliers	0	0	0	0	0
PDB entry	6KEC	6KEH	6KEK	6KEJ	6KEI

[a]Value in parentheses are for the highest resolution shell.

[b]$R_{merge} = \Sigma_h \Sigma_i | I(h)_i - < I(h) > | / \Sigma_h \Sigma_i I(h)_i$, where I(h) is the intensity for reflection h, Σ_h is the sum for all reflections, and Σ_i is the sum for i measurements of reflection h.

[c]$R = \Sigma | |F_{obs}| - |F_{calc}| | / \Sigma |F_{obs}|$, where R_{free} is calculated for a randomly chosen 5% of reflections, which were not used for structure refinement, and R_{work} is calculated for the remaining reflections.

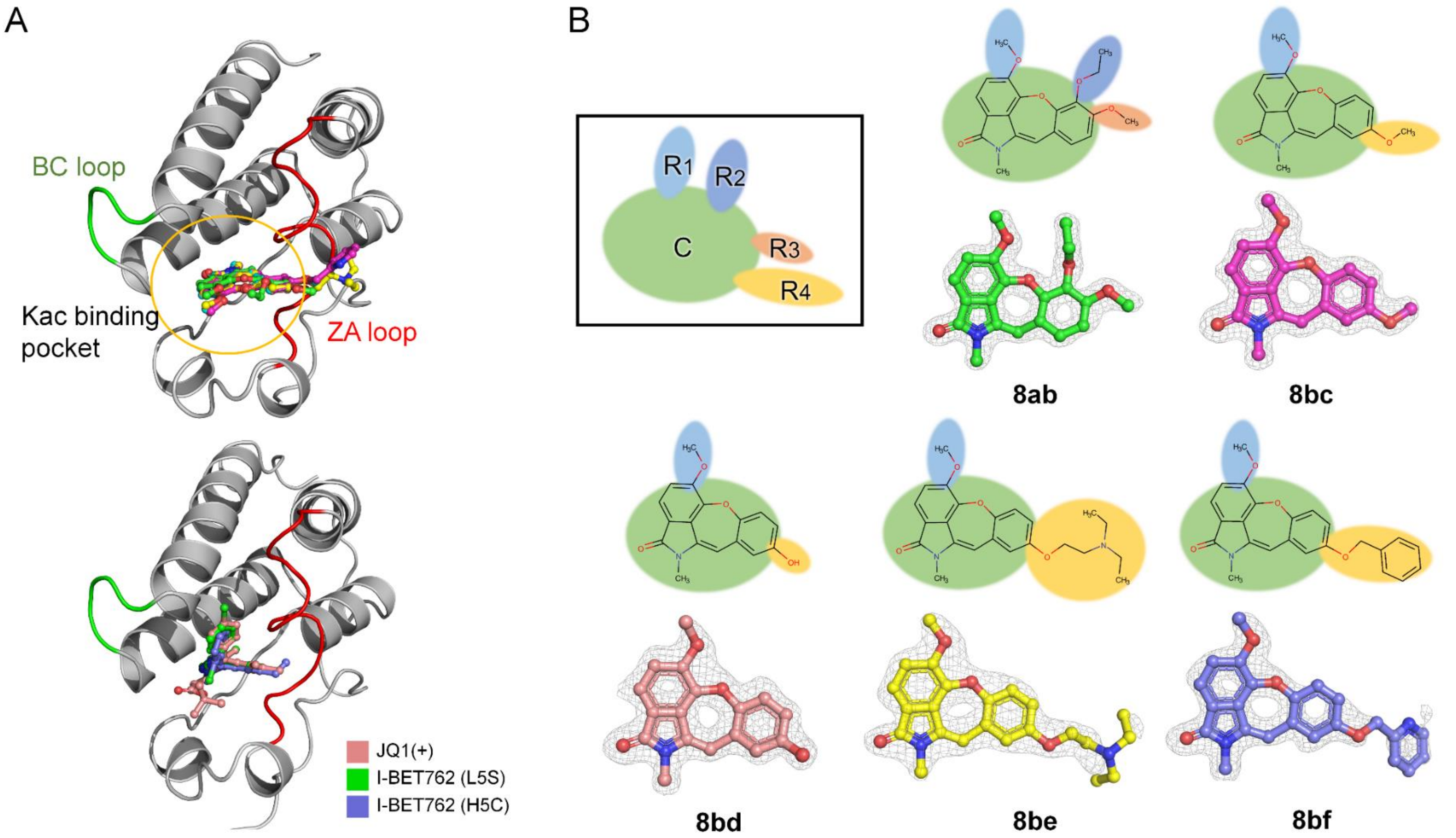

Fig. S1. Structure of BRD4 BD1 and 5 compounds analyzed in this study.

(A) Overall structure of BRD4 BD1 inhibitors, BD1-JQ1(+) (PDB entry:3MXF), and BD1-IBET762 derivatives, L5S and H5C (PDB entry:4C66, 4C67). Kac; acetylated lysine. (B) Chemical structure and omit maps (in gray and contoured at 1.0 σ) of the five inhibitors (**8ab**, **8bc**, **8bd**, **8be**, and **8bf**). The inhibitors are composed of five moieties: four ring structured core scaffold C (benzo[6,7]oxepino[4,3,2-cd]isoindol-2(1H)-one), R^2 with 4-ethyl ether (**8ab** only), and ether or hydroxyl groups at R^3- or R^4-position (5-methyl ether, 6-methyl ether, 6-benzylmethyl ether, 6-[2-(diethylamino)ethyl] ether, 6-hydroxide)

Compound 2a

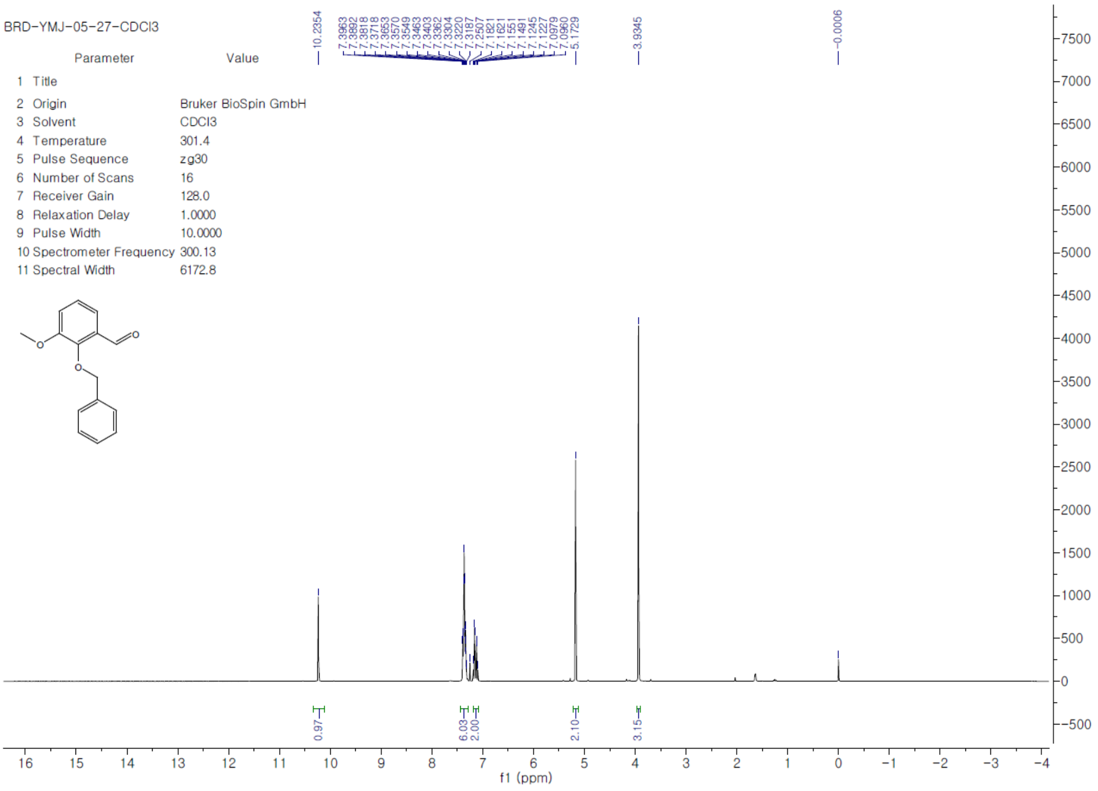

Compound 2b

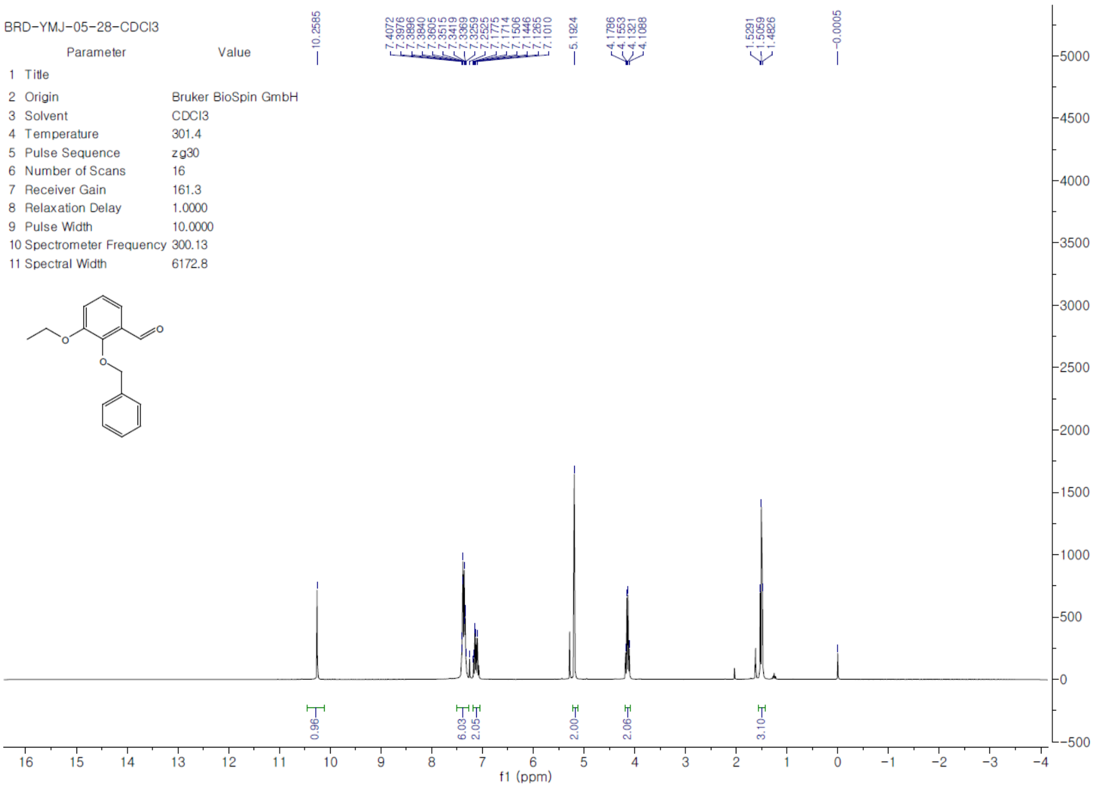

Compound 2c

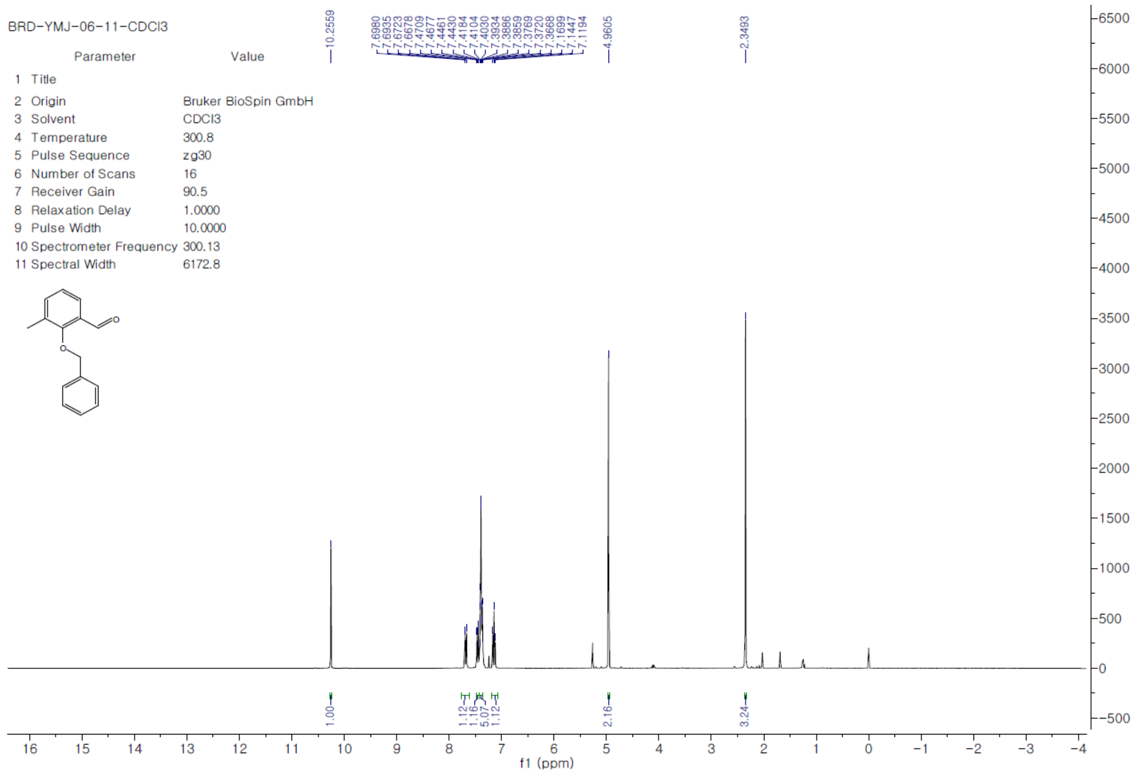

Compound 2d

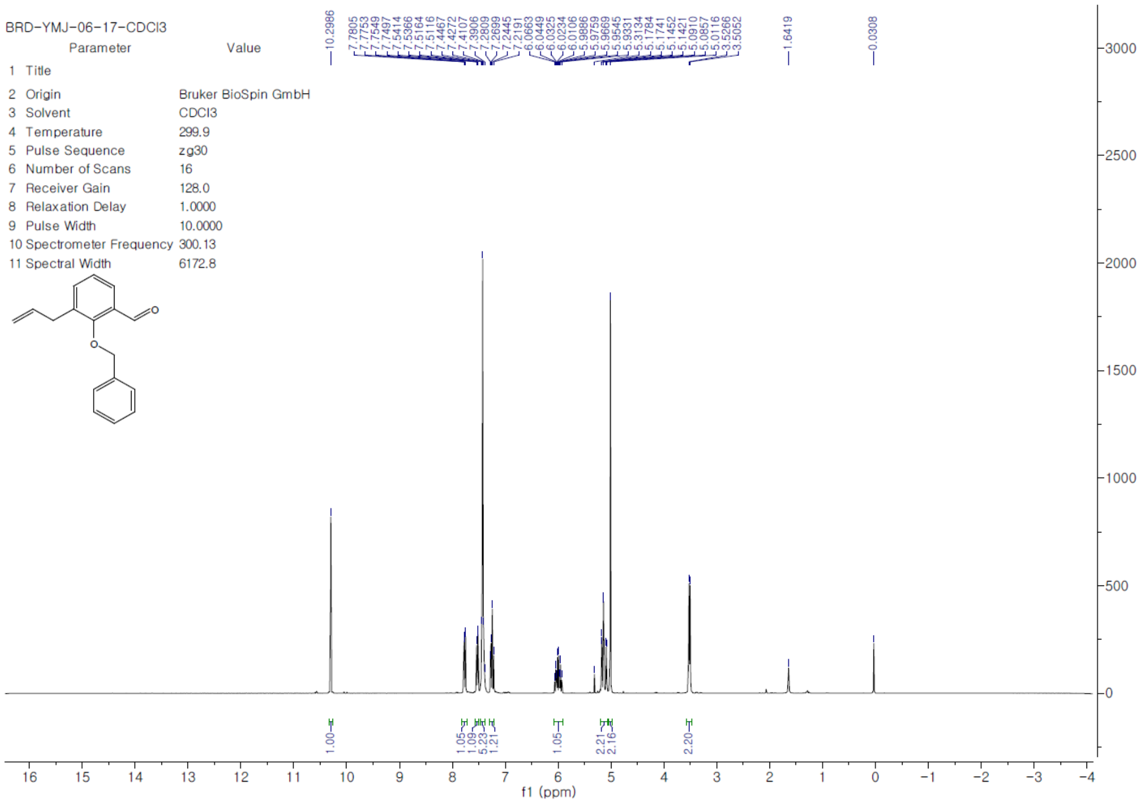

Compound 2e

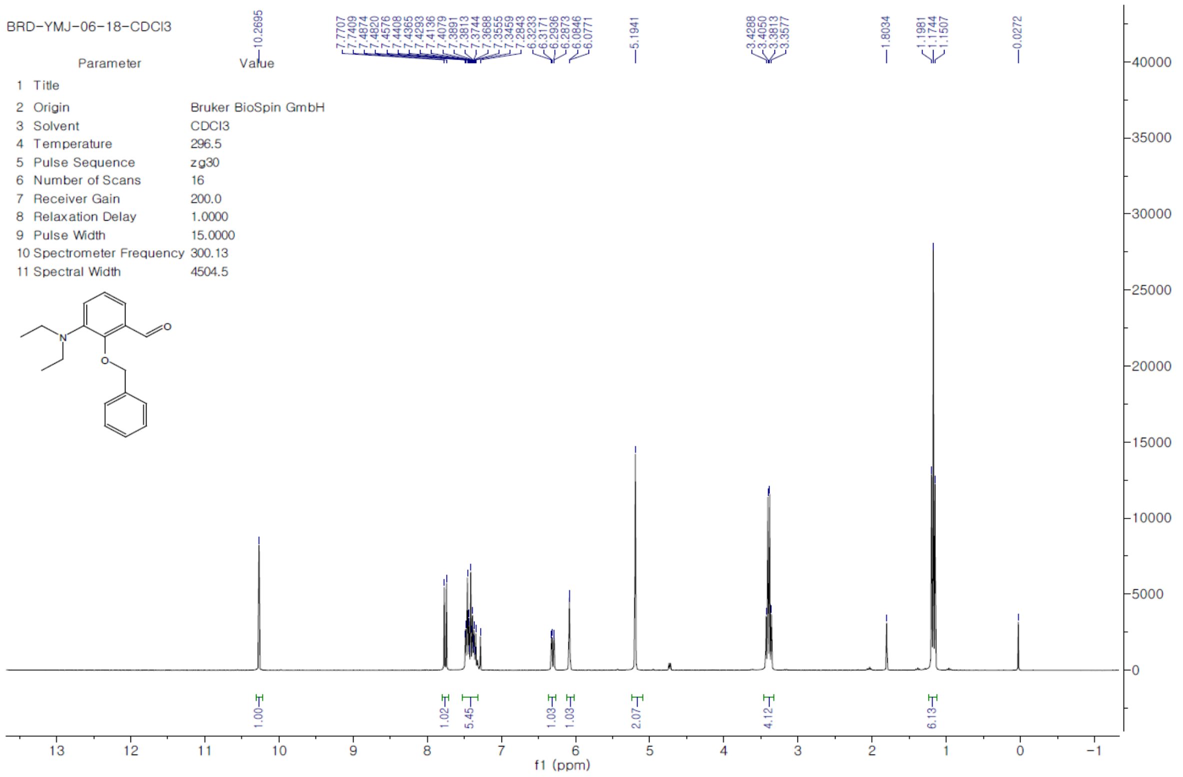

Compound 3a

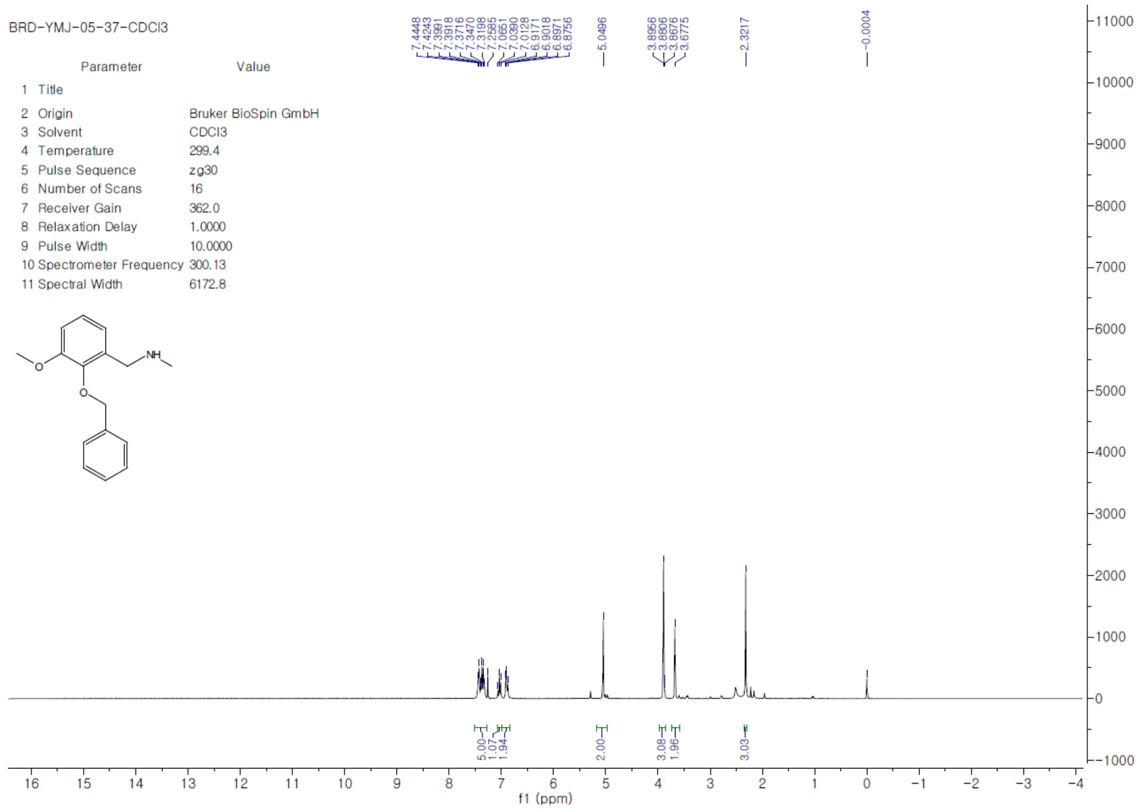

Compound 3b

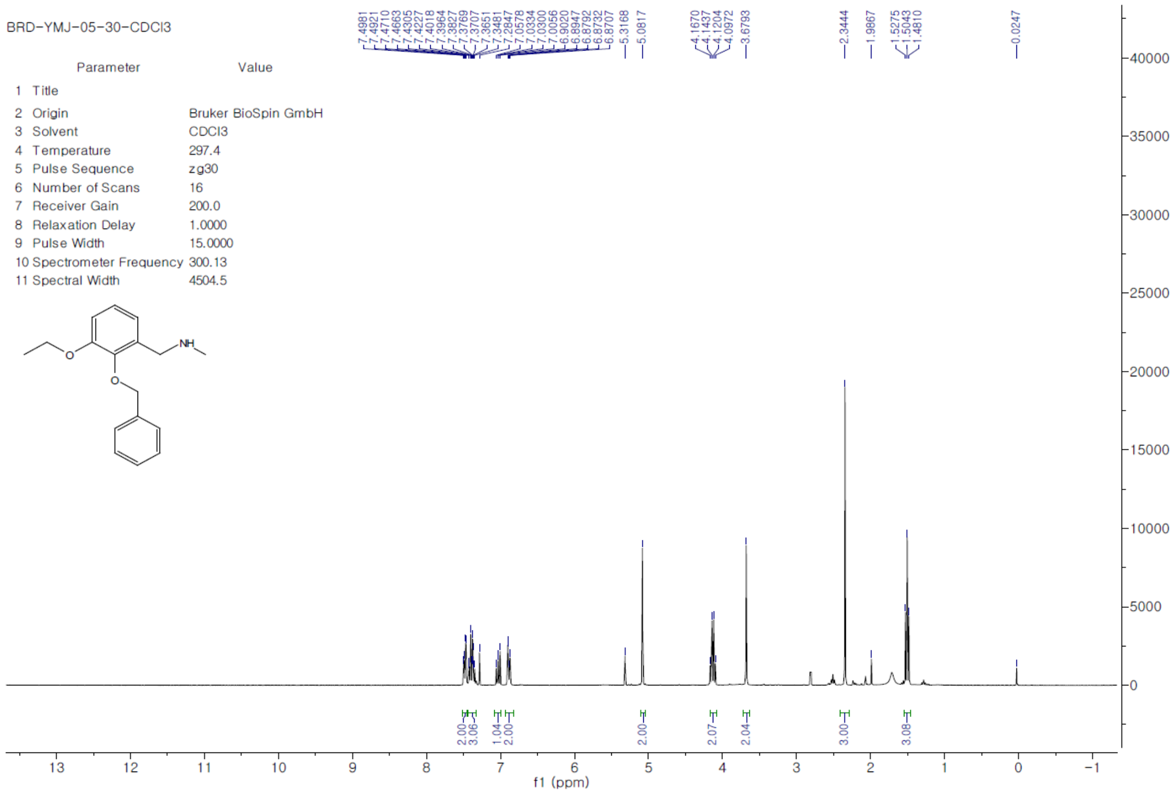

Compound 3c

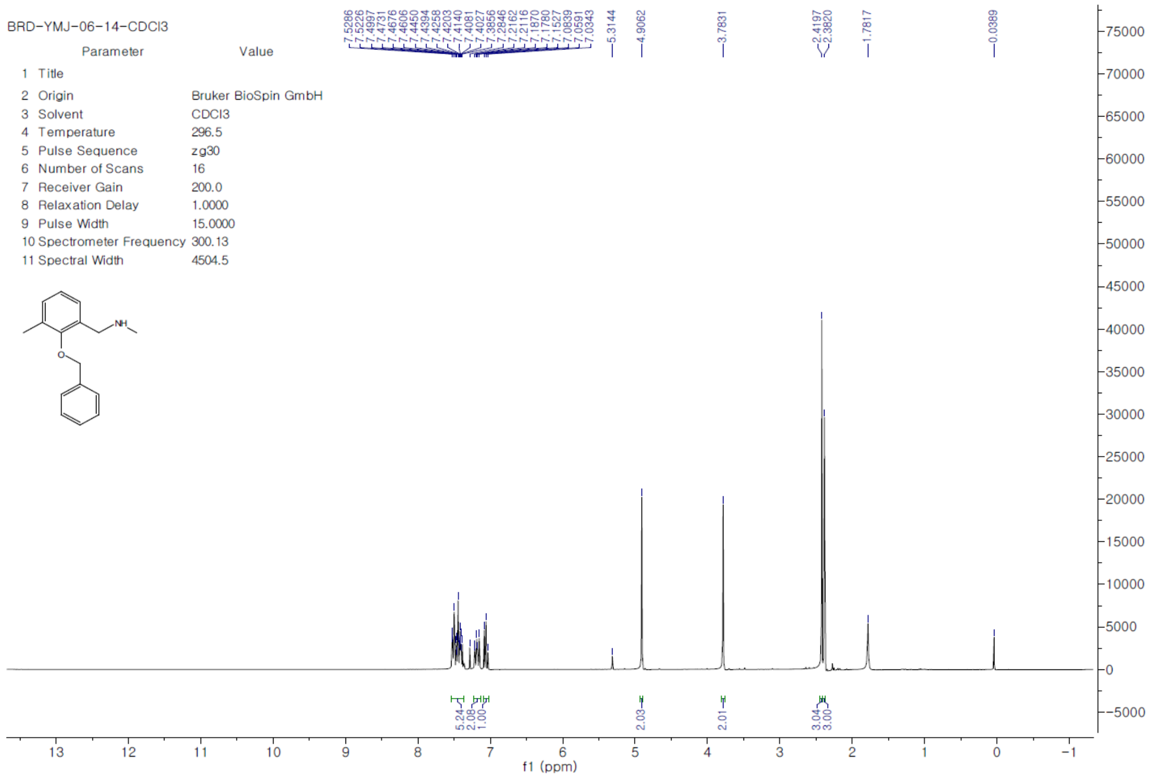

Compound 3d

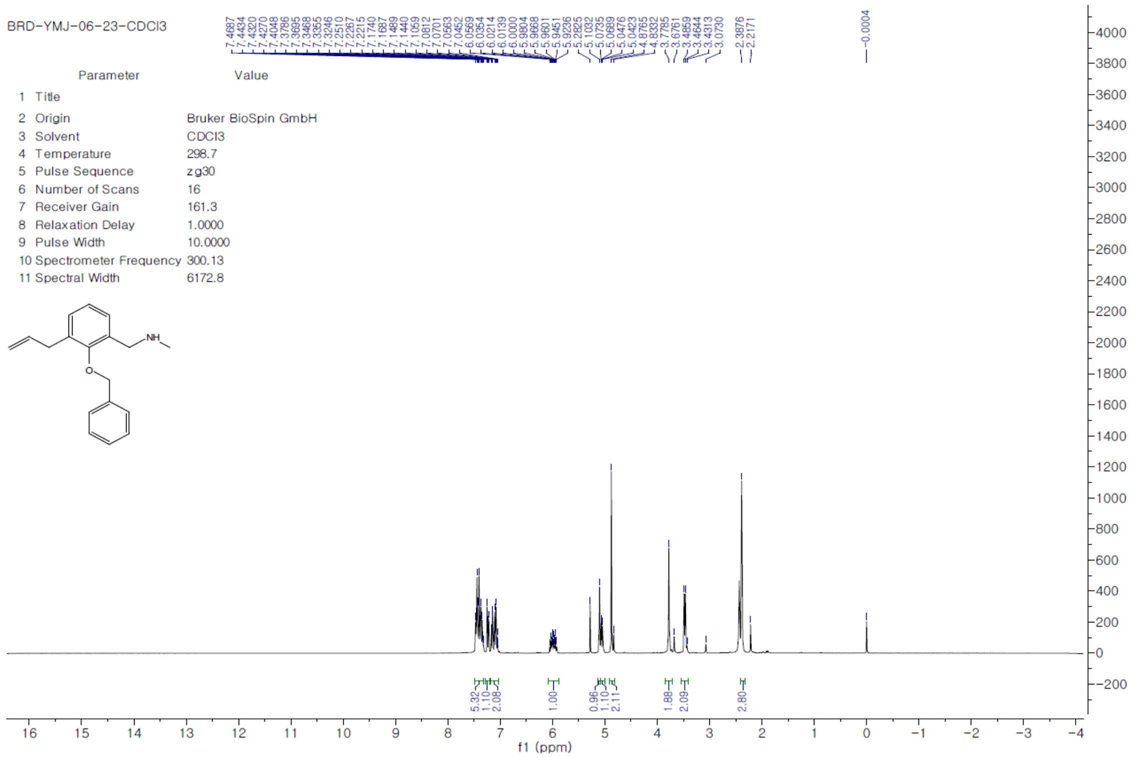

Compound 3e

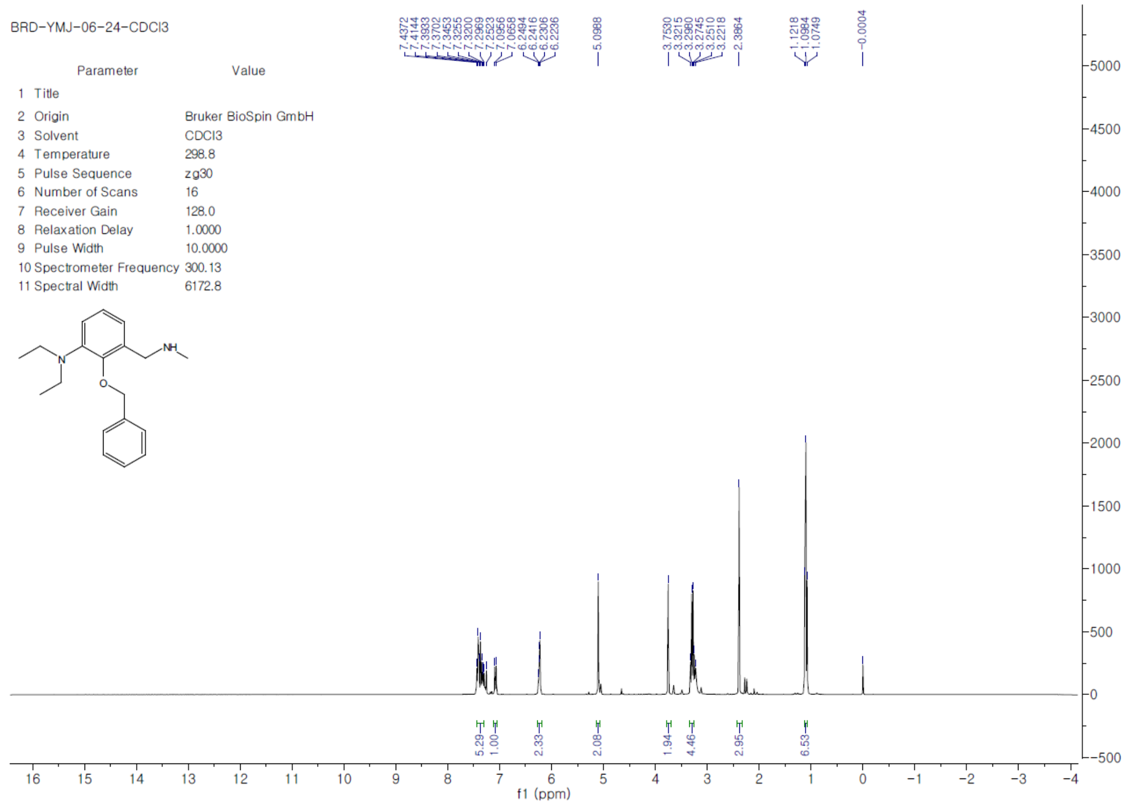

Compound 4a

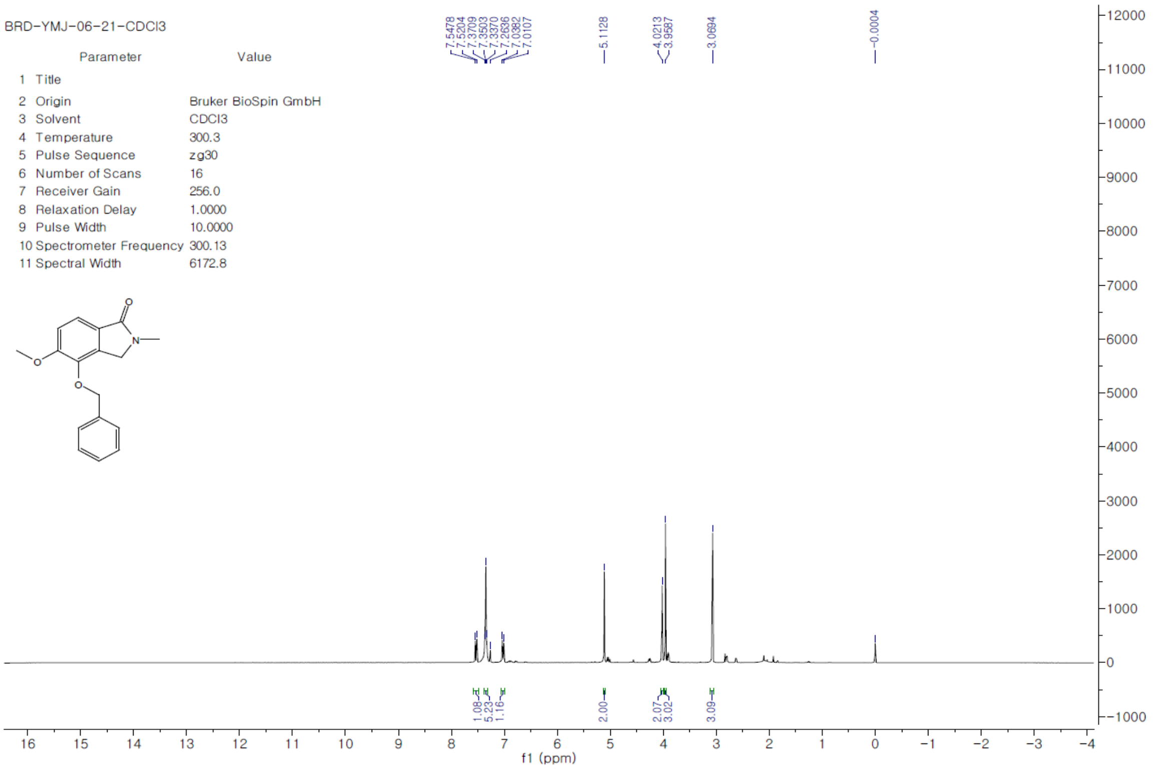

Compound 4b

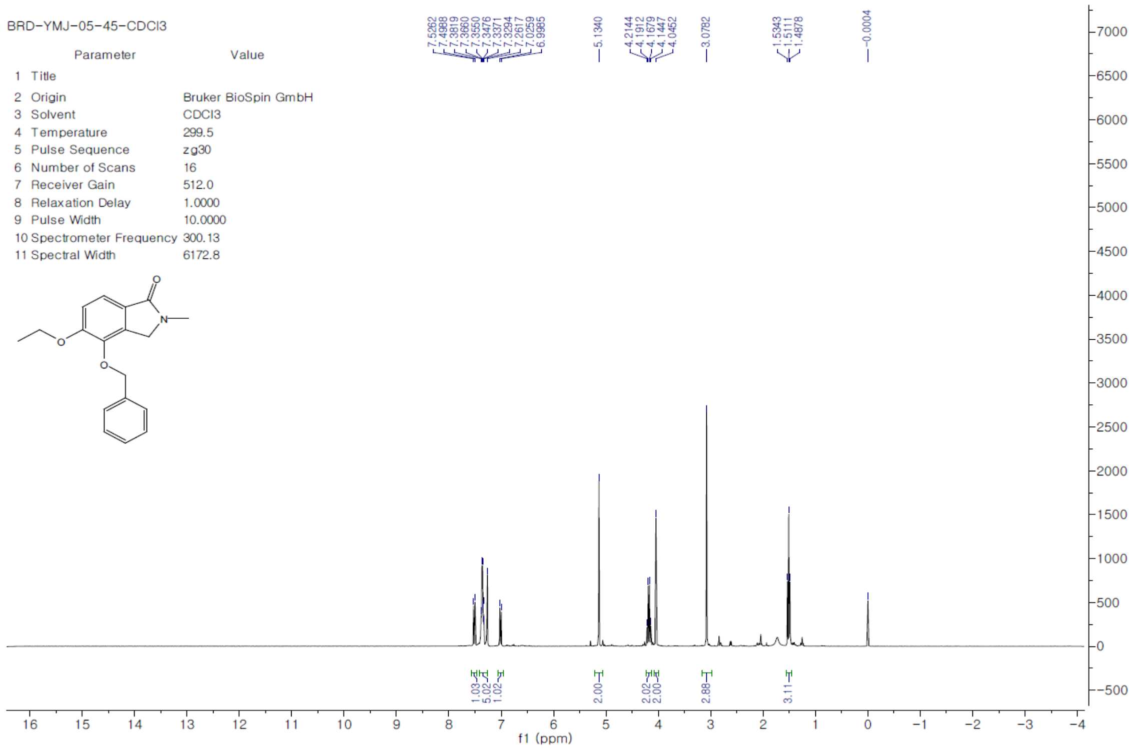

Compound 4c

BRD−YMJ−06−20−CDCl3

	Parameter	Value
1	Title	
2	Origin	Bruker BioSpin GmbH
3	Solvent	CDCl3
4	Temperature	300.0
5	Pulse Sequence	zg30
6	Number of Scans	16
7	Receiver Gain	161.3
8	Relaxation Delay	1.0000
9	Pulse Width	10.0000
10	Spectrometer Frequency	300.13
11	Spectral Width	6172.8

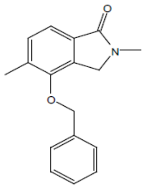

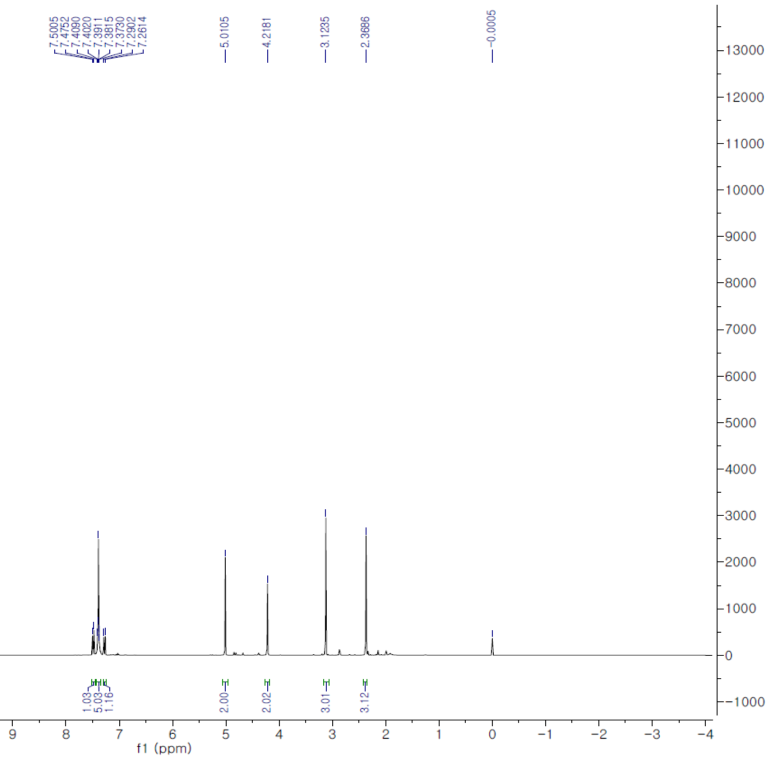

Compound 4d

BRD−YMJ−06−40−CDCl3

	Parameter	Value
1	Title	
2	Origin	Bruker BioSpin GmbH
3	Solvent	CDCl3
4	Temperature	300.1
5	Pulse Sequence	zg30
6	Number of Scans	16
7	Receiver Gain	200.0
8	Relaxation Delay	1.0000
9	Pulse Width	15.0000
10	Spectrometer Frequency	300.13
11	Spectral Width	4504.5

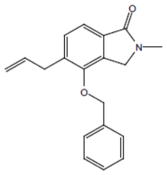

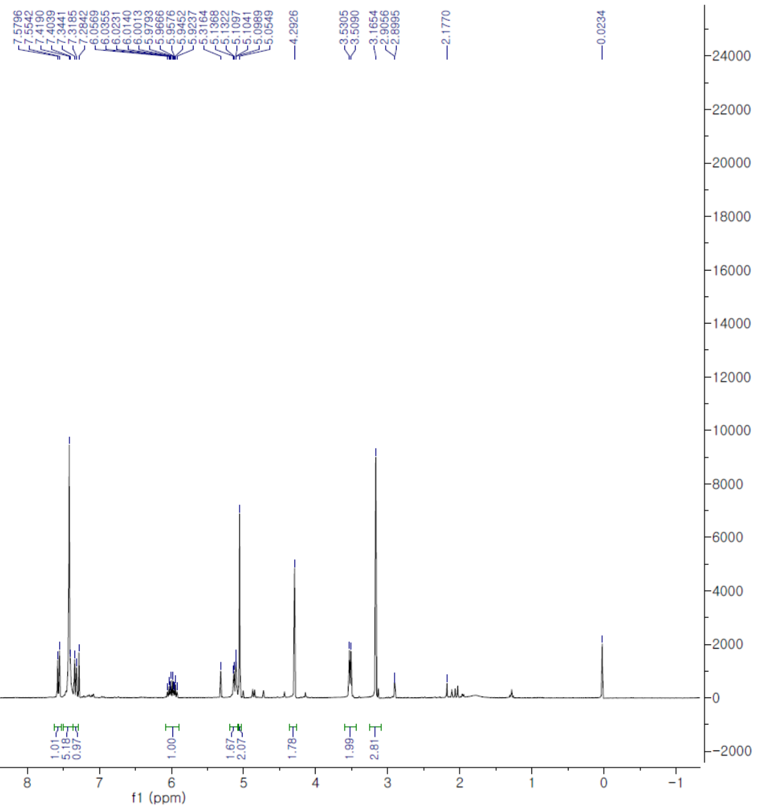

Compound 4e

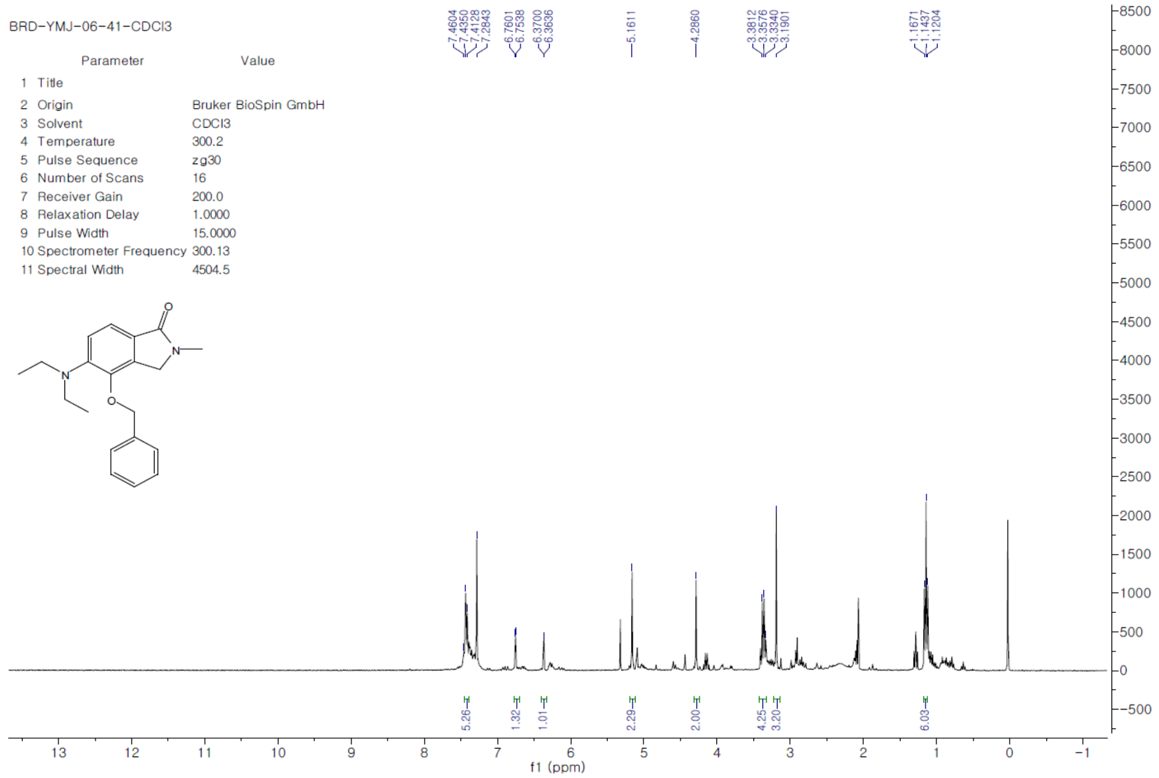

Compound 5a

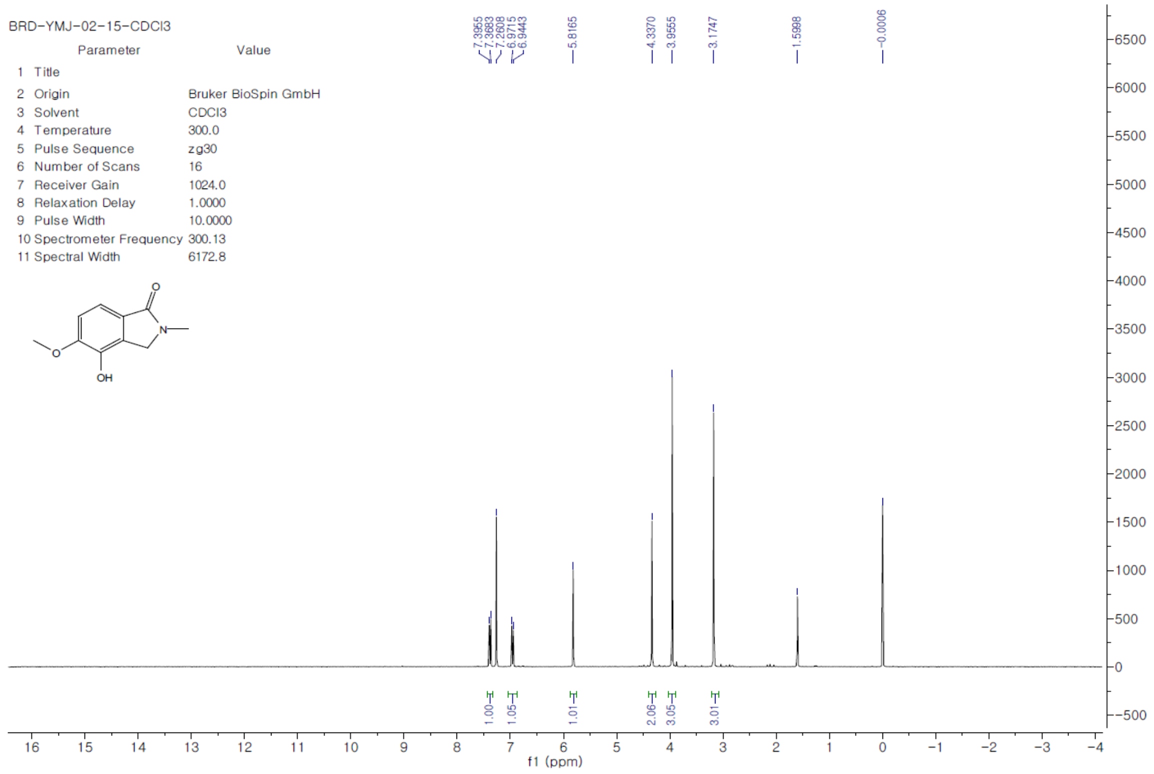

Compound 5b

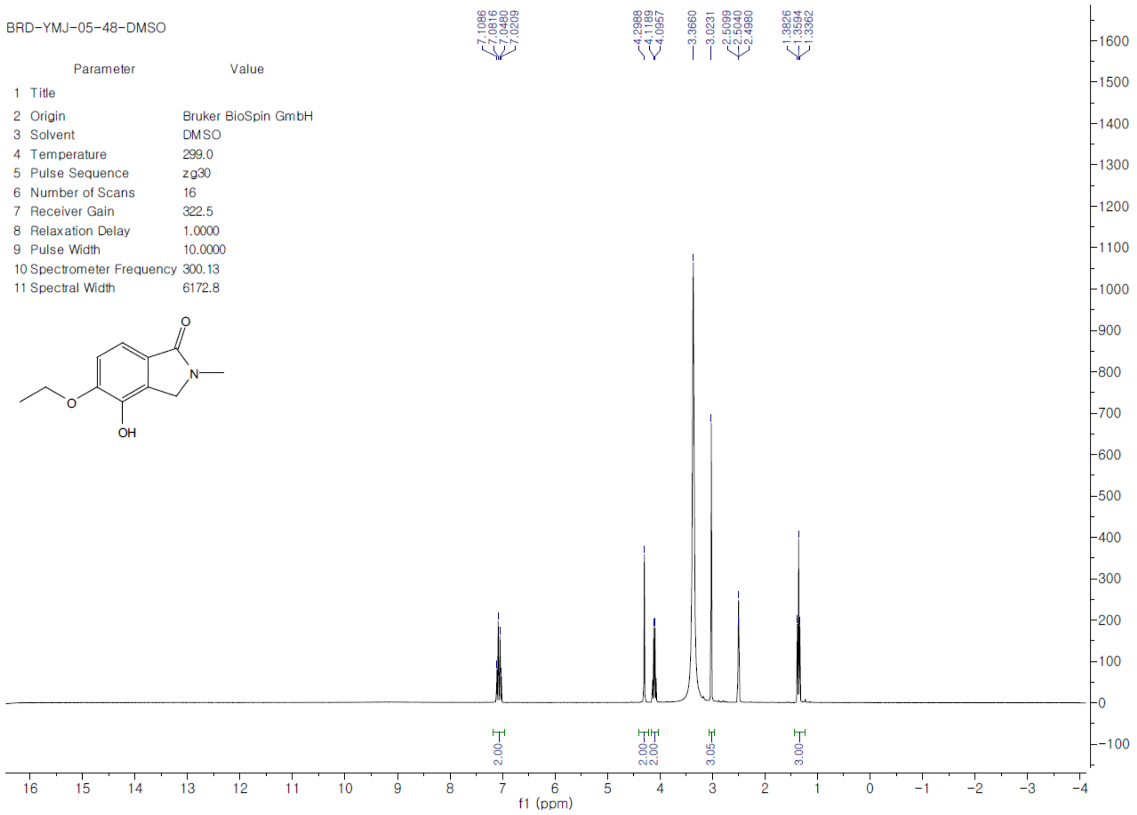
BRD-YMJ-05-48-DMSO
Parameter Value
1 Title
2 Origin Bruker BioSpin GmbH
3 Solvent DMSO
4 Temperature 299.0
5 Pulse Sequence zg30
6 Number of Scans 16
7 Receiver Gain 322.5
8 Relaxation Delay 1.0000
9 Pulse Width 10.0000
10 Spectrometer Frequency 300.13
11 Spectral Width 6172.8
7.1086
7.0816
7.0480
7.0209
4.2988
4.1189
4.0957
3.3660
3.0231
2.5099
2.5040
2.4980
1.3826
1.3594
1.3362
2.00
2.00
2.00
3.05
3.00
O
N
O
OH
f1 (ppm)

Compound 5c

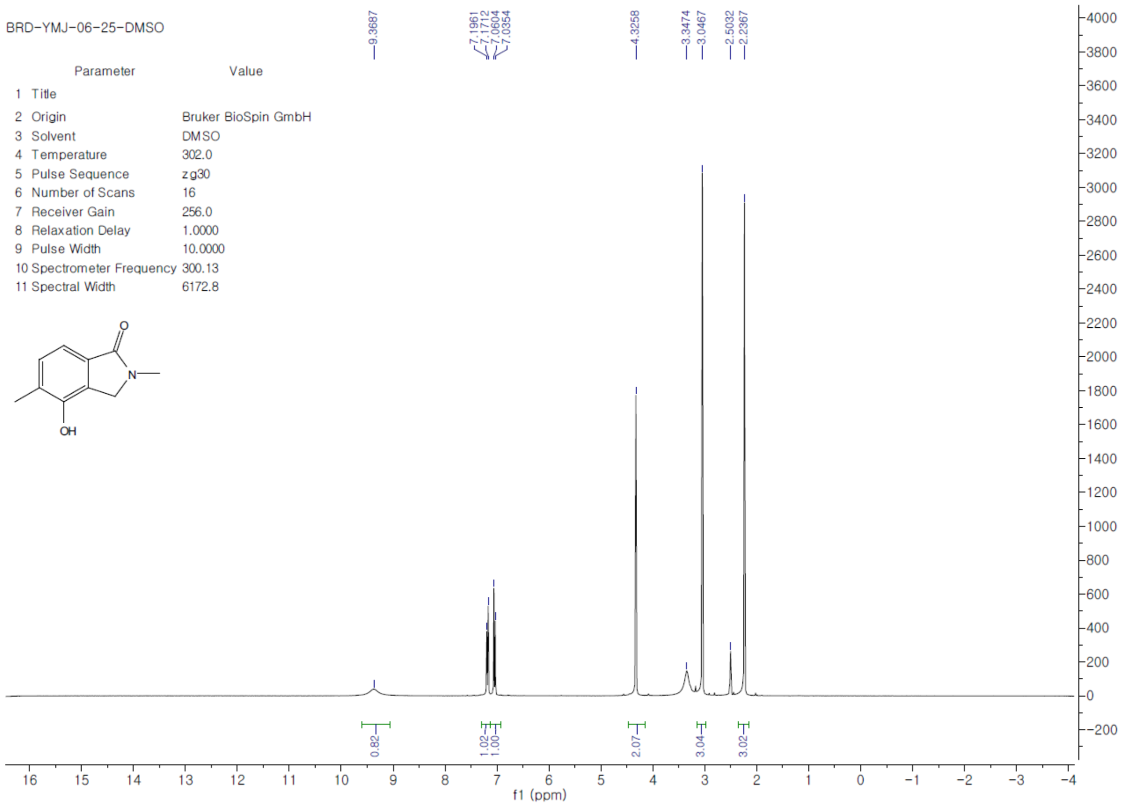
BRD-YMJ-06-25-DMSO
Parameter Value
1 Title
2 Origin Bruker BioSpin GmbH
3 Solvent DMSO
4 Temperature 302.0
5 Pulse Sequence zg30
6 Number of Scans 16
7 Receiver Gain 256.0
8 Relaxation Delay 1.0000
9 Pulse Width 10.0000
10 Spectrometer Frequency 300.13
11 Spectral Width 6172.8
9.3687
7.1961
7.1712
7.0604
7.0354
4.3258
3.3474
3.0467
2.5032
2.2367
0.82
1.02
1.00
2.07
3.04
3.02
O
N
OH
f1 (ppm)

Compound 5d

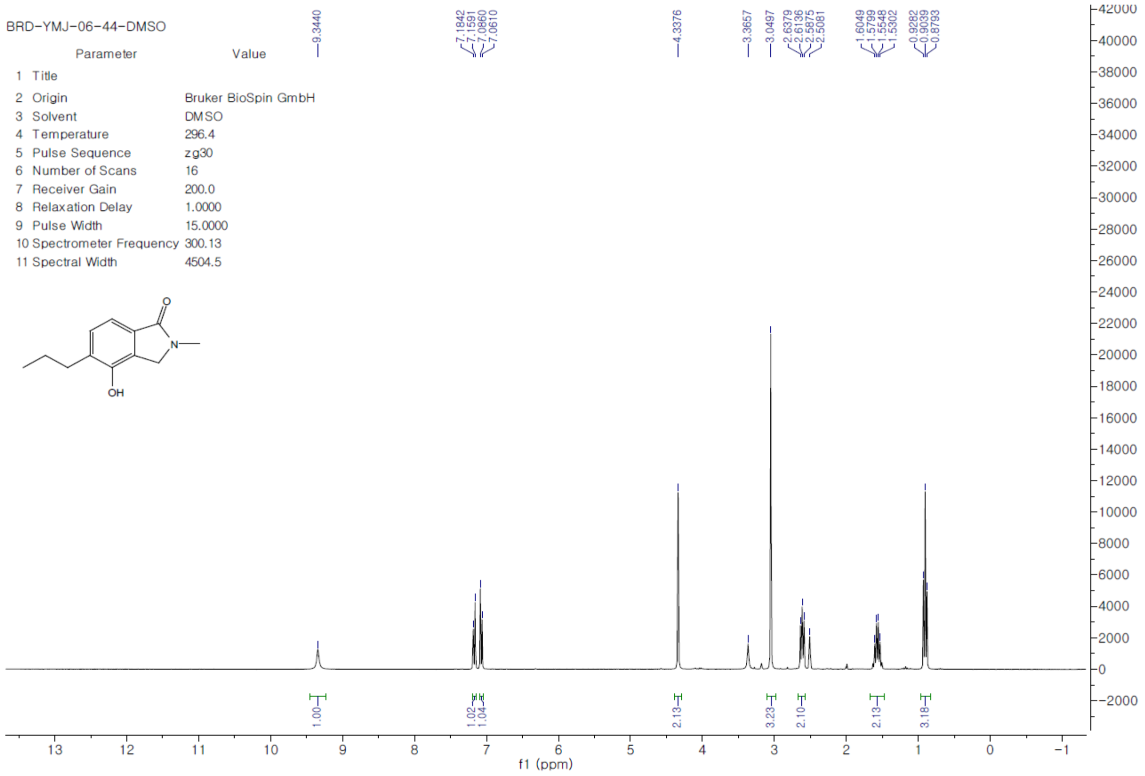

Compound 5e

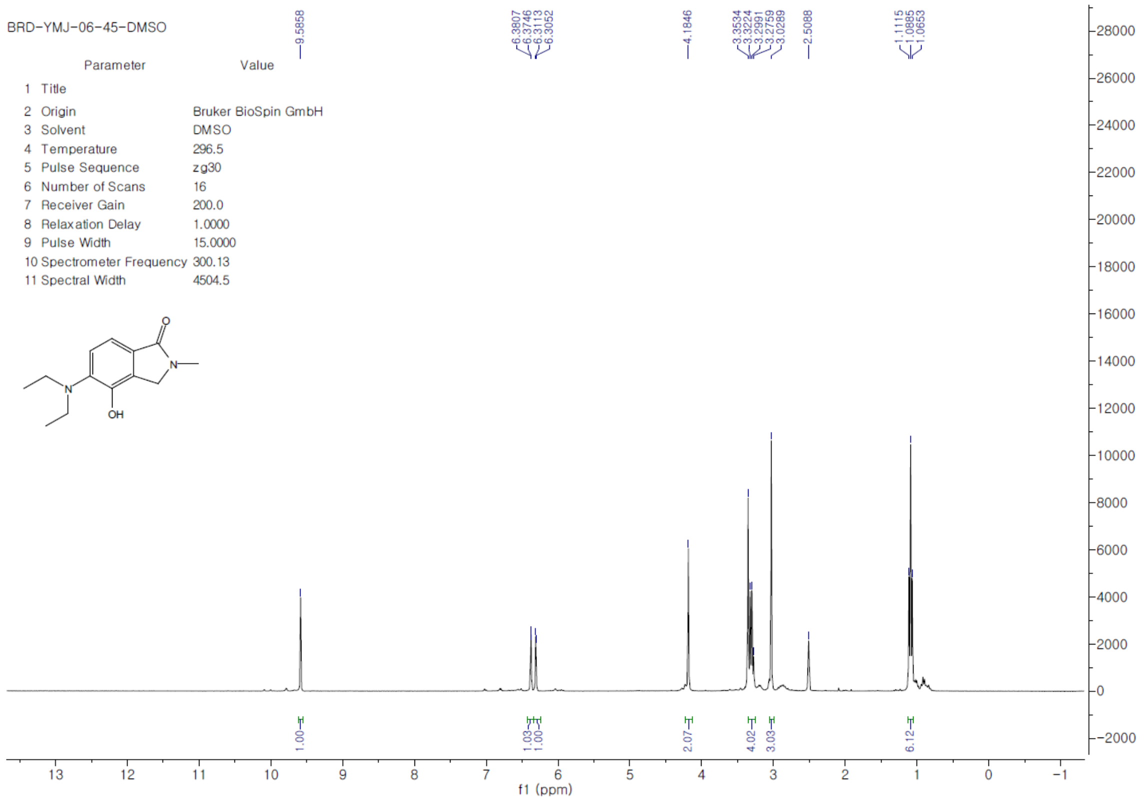

Compound 6a

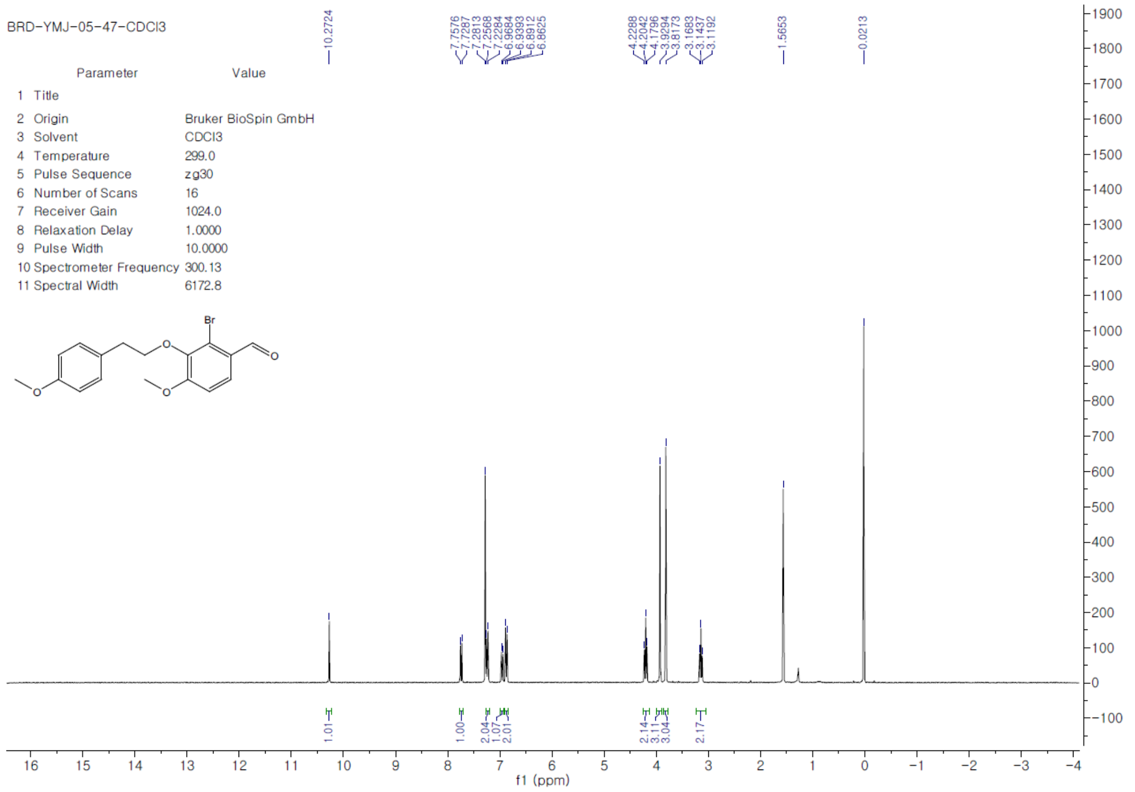

Compound 6b

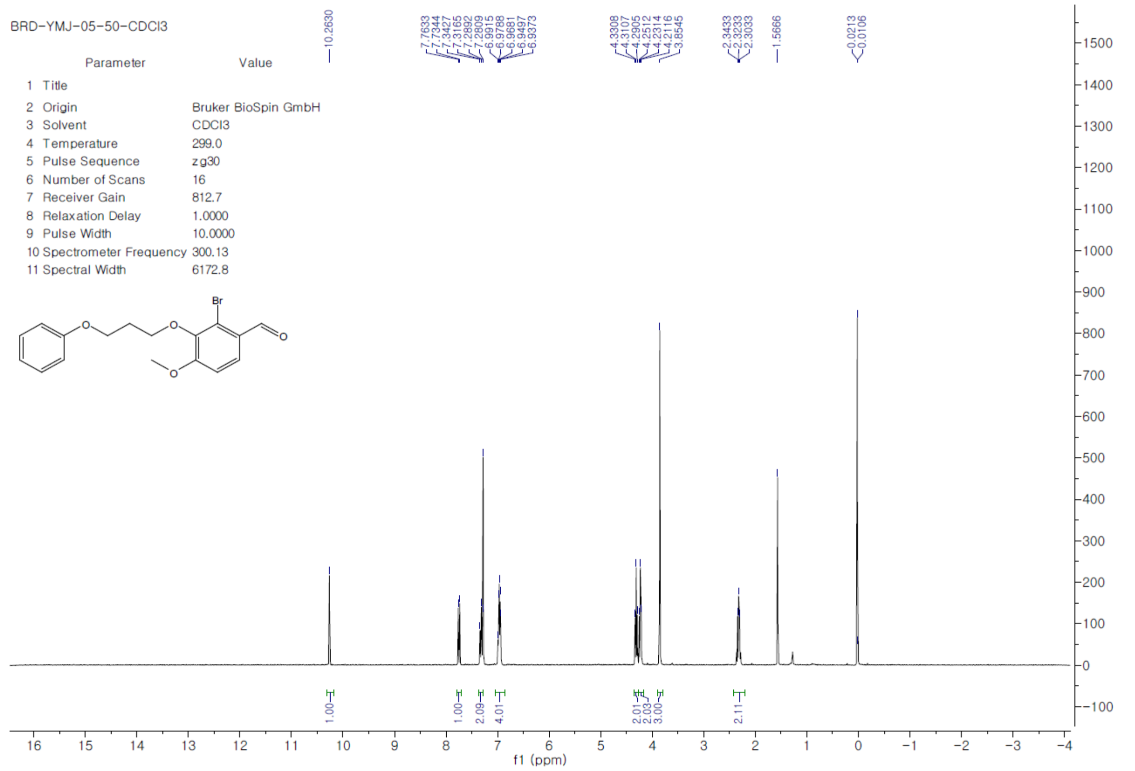

Compound 8aa

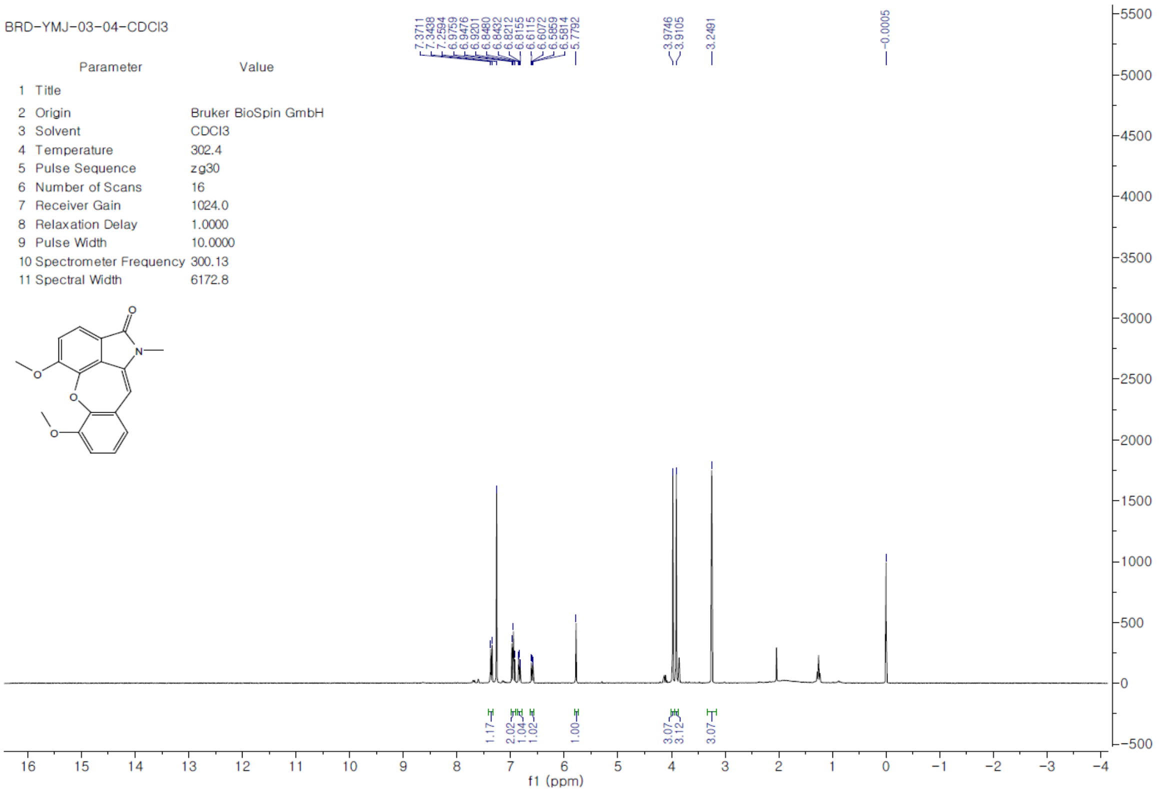

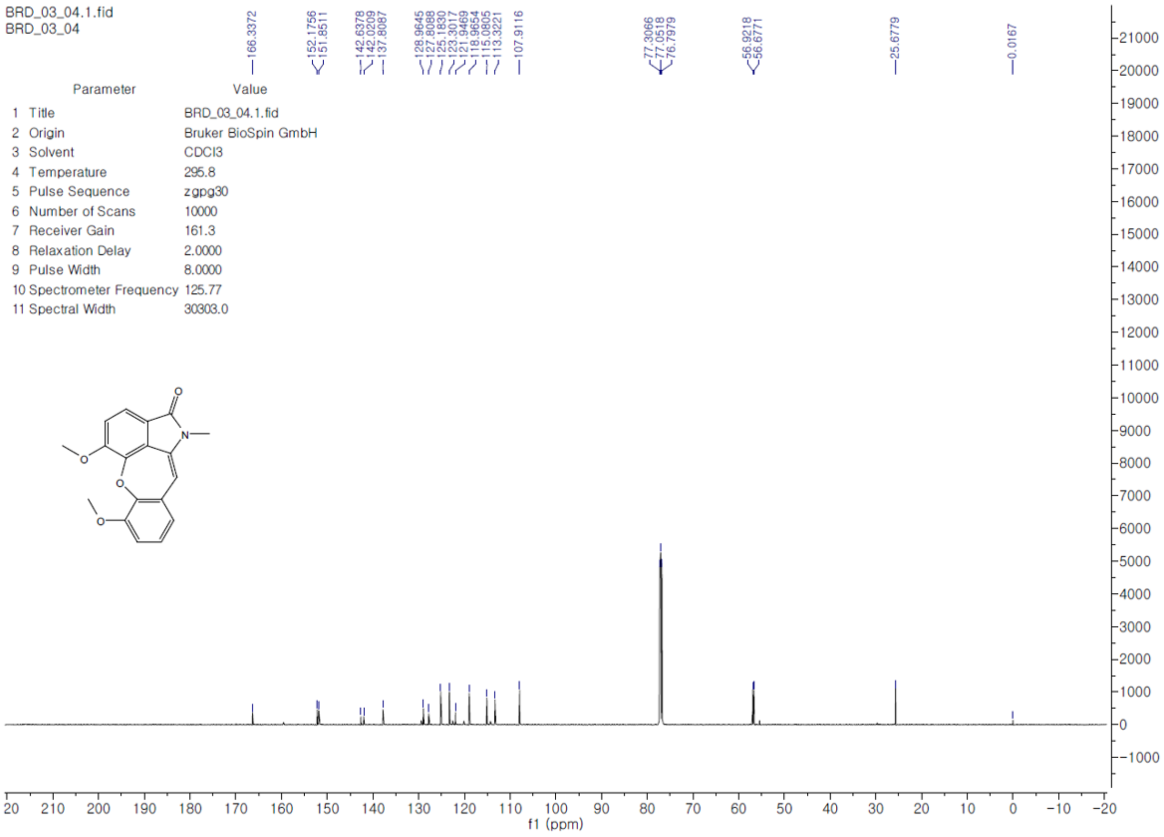

Compound 8ab

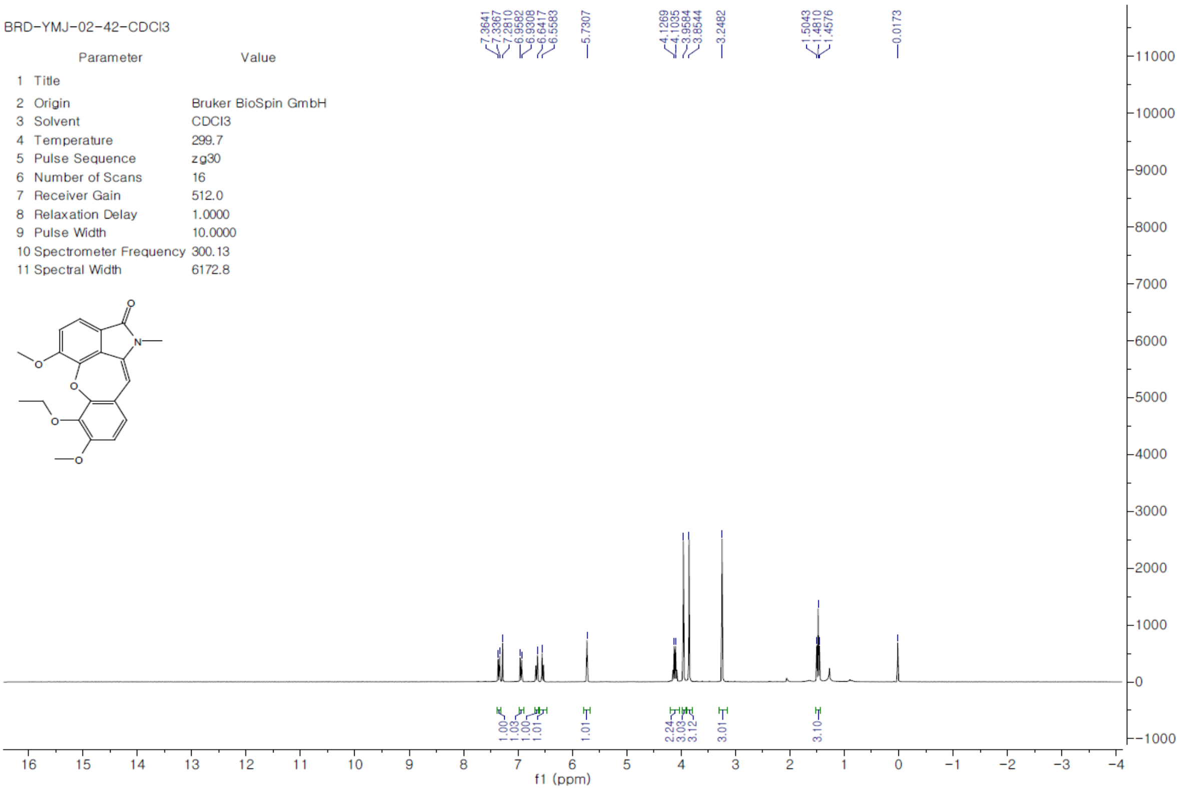

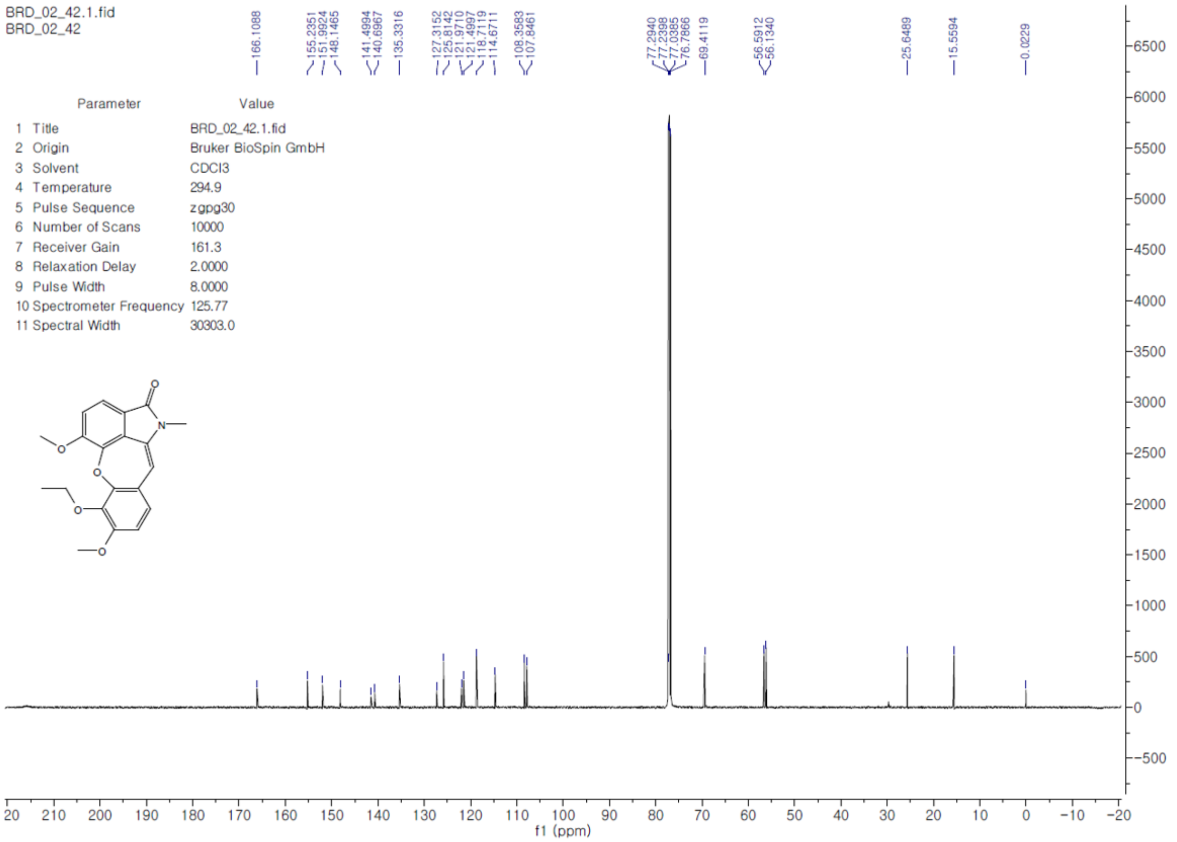

Compound 8ac

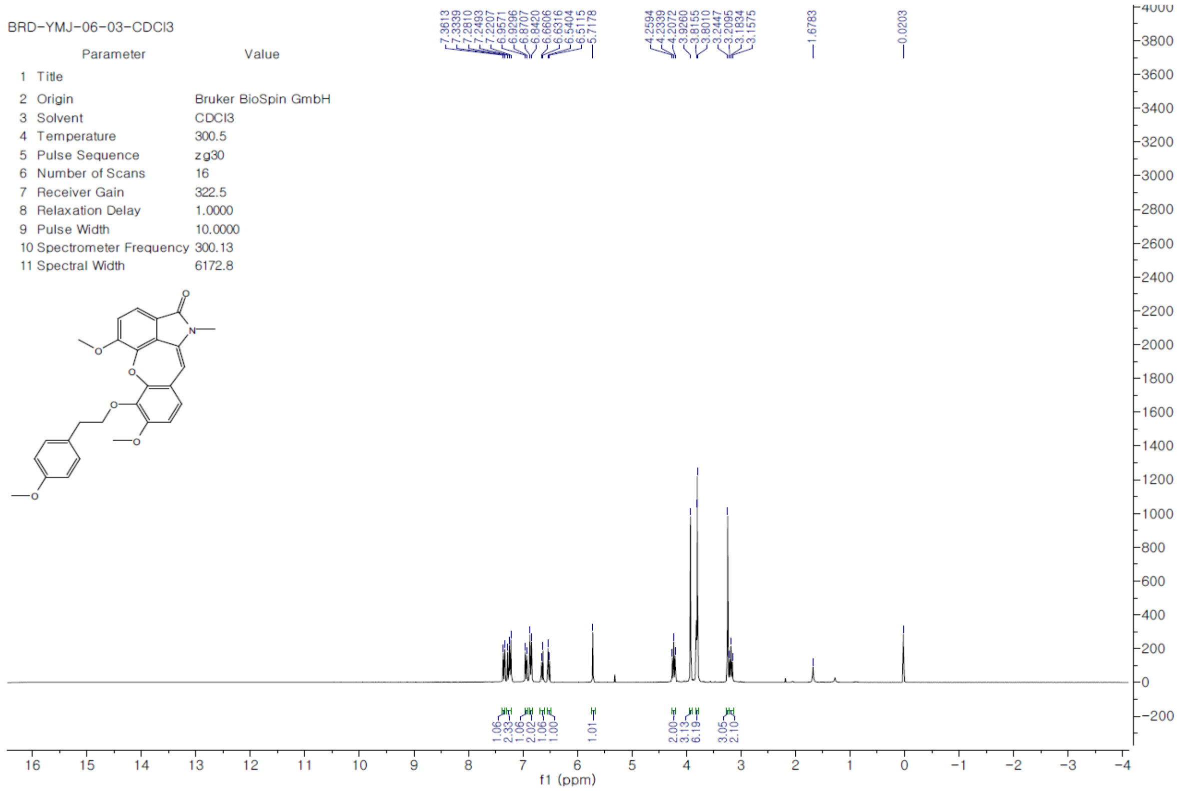

	Parameter	Value
1	Title	
2	Origin	Bruker BioSpin GmbH
3	Solvent	CDCl3
4	Temperature	300.5
5	Pulse Sequence	zg30
6	Number of Scans	16
7	Receiver Gain	322.5
8	Relaxation Delay	1.0000
9	Pulse Width	10.0000
10	Spectrometer Frequency	300.13
11	Spectral Width	6172.8

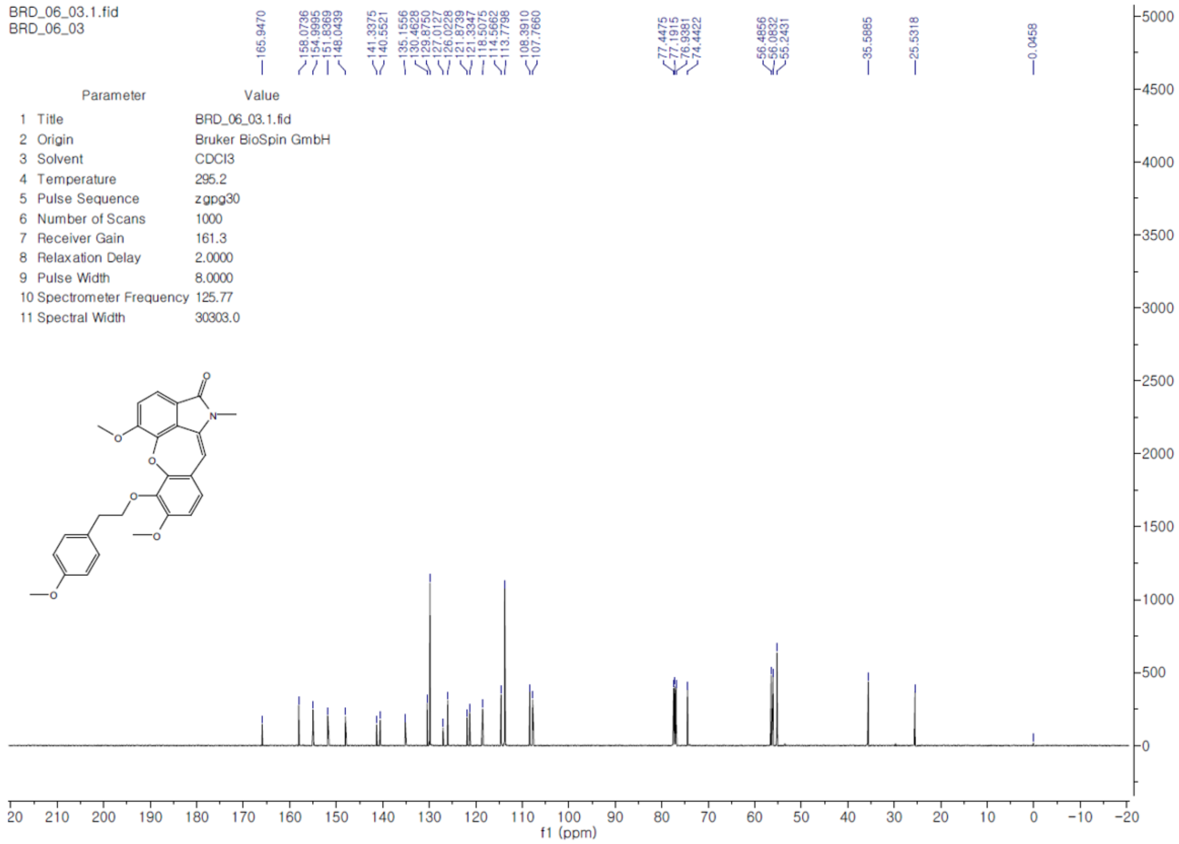

	Parameter	Value
1	Title	BRD_06_03.1.fid
2	Origin	Bruker BioSpin GmbH
3	Solvent	CDCl3
4	Temperature	295.2
5	Pulse Sequence	zgpg30
6	Number of Scans	1000
7	Receiver Gain	161.3
8	Relaxation Delay	2.0000
9	Pulse Width	8.0000
10	Spectrometer Frequency	125.77
11	Spectral Width	30303.0

Compound 8ad

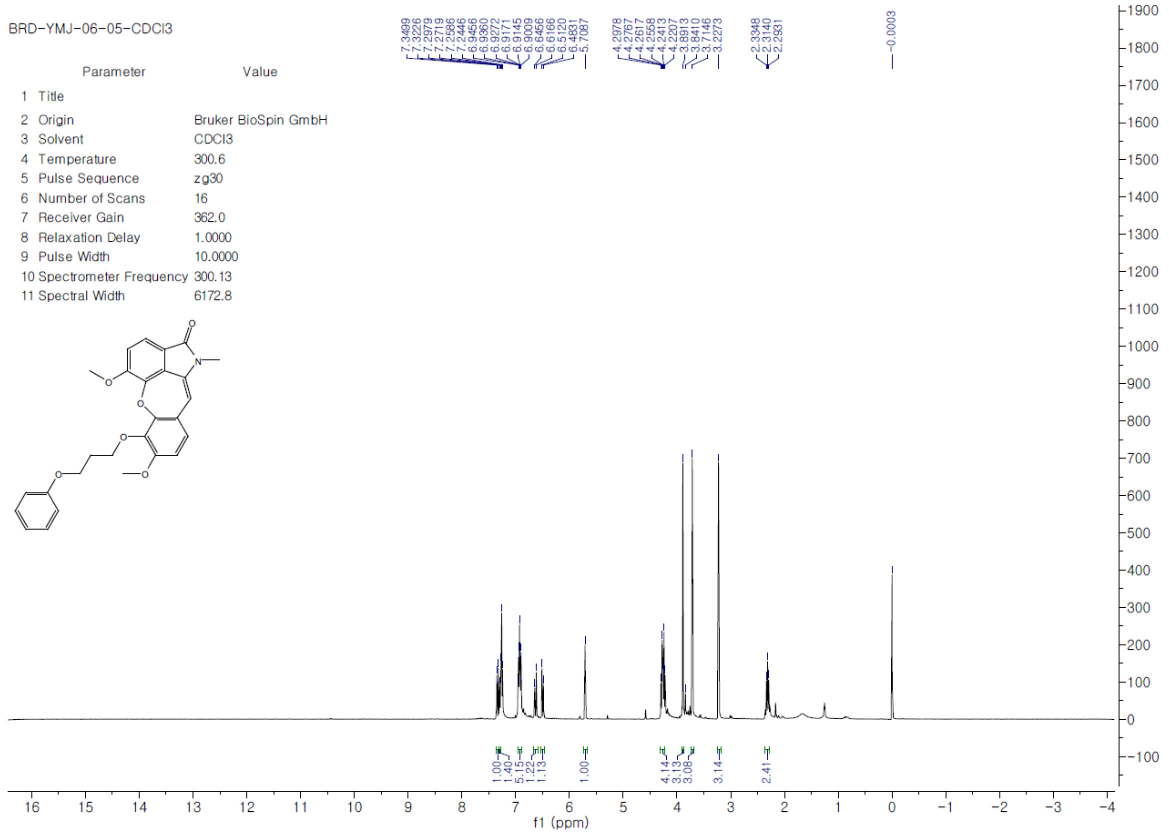

Compound 8ae

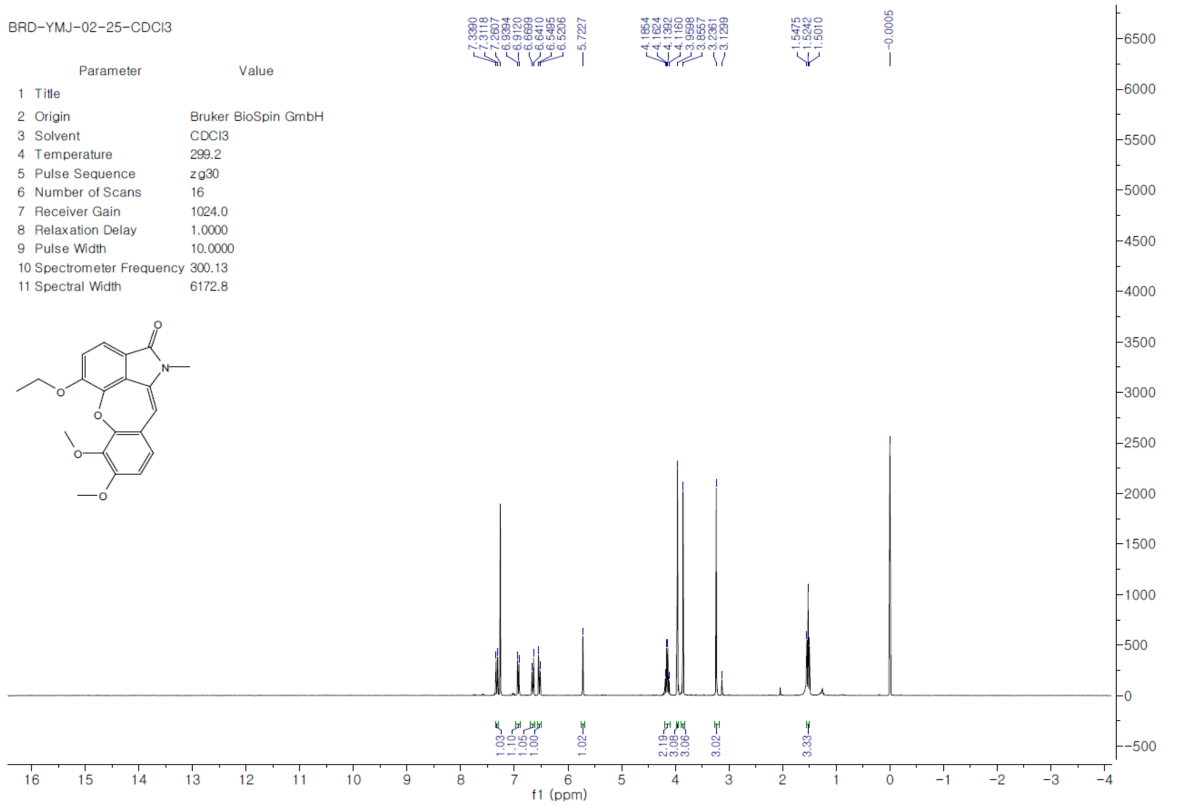

	Parameter	Value
1	Title	
2	Origin	Bruker BioSpin GmbH
3	Solvent	CDCl3
4	Temperature	299.2
5	Pulse Sequence	zg30
6	Number of Scans	16
7	Receiver Gain	1024.0
8	Relaxation Delay	1.0000
9	Pulse Width	10.0000
10	Spectrometer Frequency	300.13
11	Spectral Width	6172.8

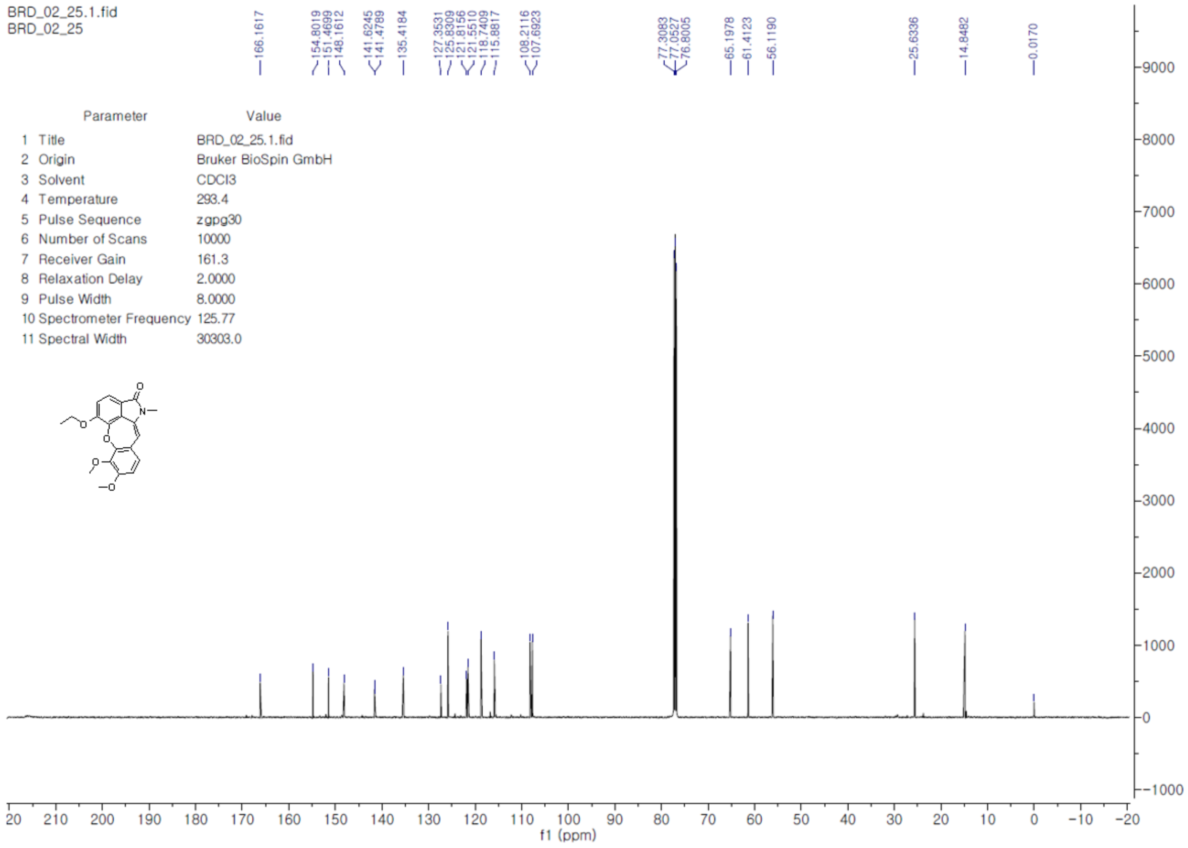

	Parameter	Value
1	Title	BRD_02_25.1.fid
2	Origin	Bruker BioSpin GmbH
3	Solvent	CDCl3
4	Temperature	293.4
5	Pulse Sequence	zgpg30
6	Number of Scans	10000
7	Receiver Gain	161.3
8	Relaxation Delay	2.0000
9	Pulse Width	8.0000
10	Spectrometer Frequency	125.77
11	Spectral Width	30303.0

Compound 8af

BRD-YMJ-06-04-CDCl3

	Parameter	Value
1	Title	
2	Origin	Bruker BioSpin GmbH
3	Solvent	CDCl3
4	Temperature	300.6
5	Pulse Sequence	zg30
6	Number of Scans	16
7	Receiver Gain	128.0
8	Relaxation Delay	1.0000
9	Pulse Width	10.0000
10	Spectrometer Frequency	300.13
11	Spectral Width	6172.8

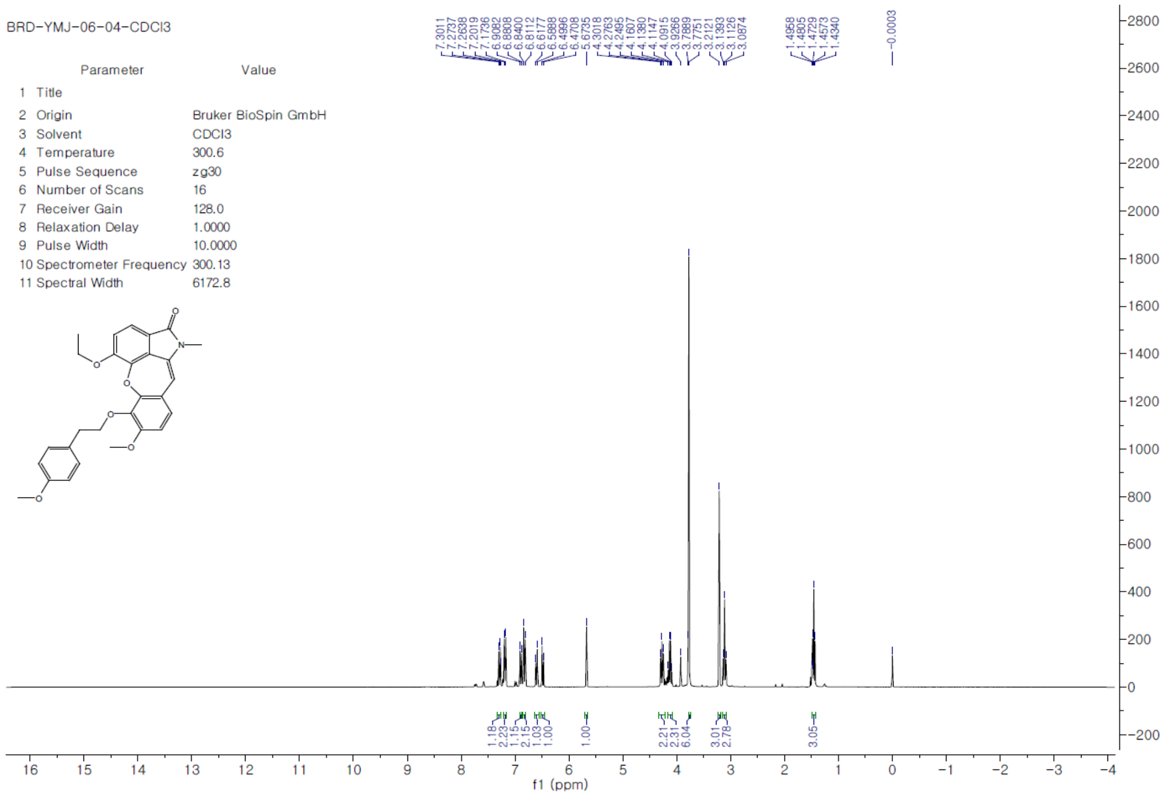

BRD_06_04.1.fid
BRD_06_04

	Parameter	Value
1	Title	BRD_06_04.1.fid
2	Origin	Bruker BioSpin GmbH
3	Solvent	CDCl3
4	Temperature	294.9
5	Pulse Sequence	zgpg30
6	Number of Scans	1000
7	Receiver Gain	161.3
8	Relaxation Delay	2.0000
9	Pulse Width	8.0000
10	Spectrometer Frequency	125.77
11	Spectral Width	30303.0

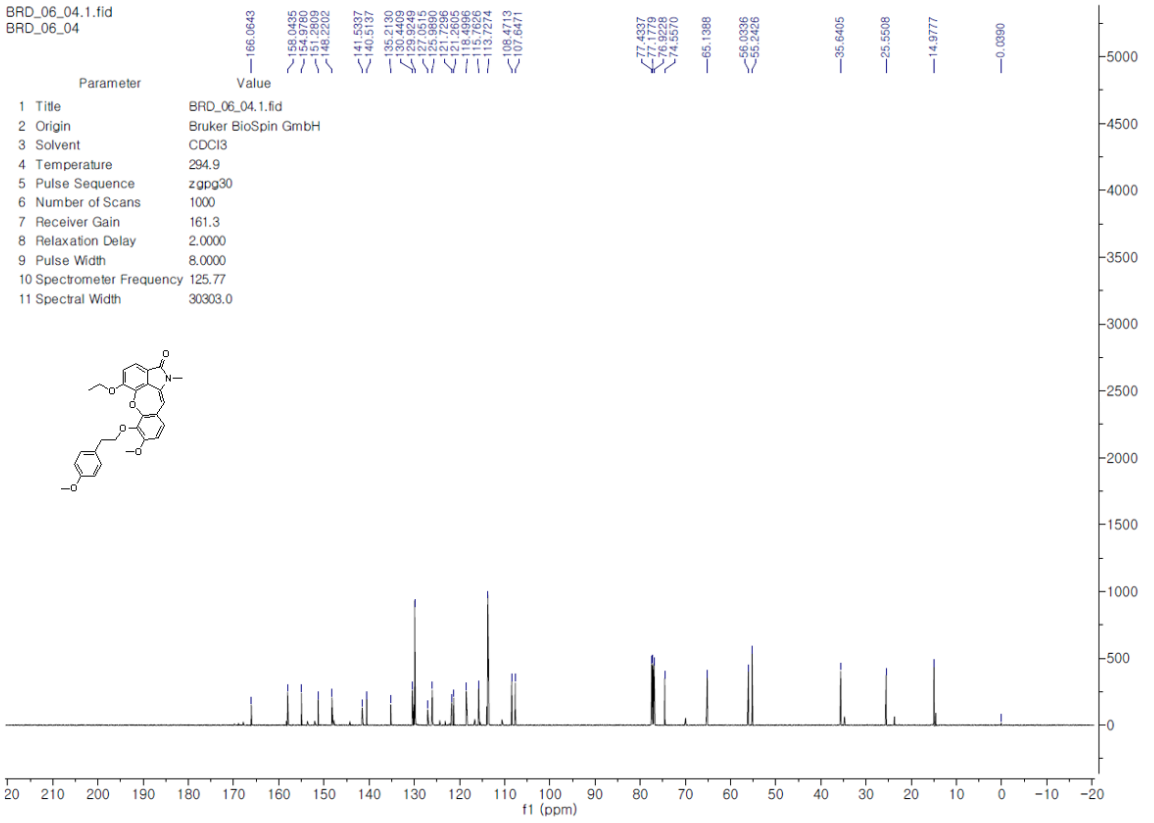

Compound 8ba

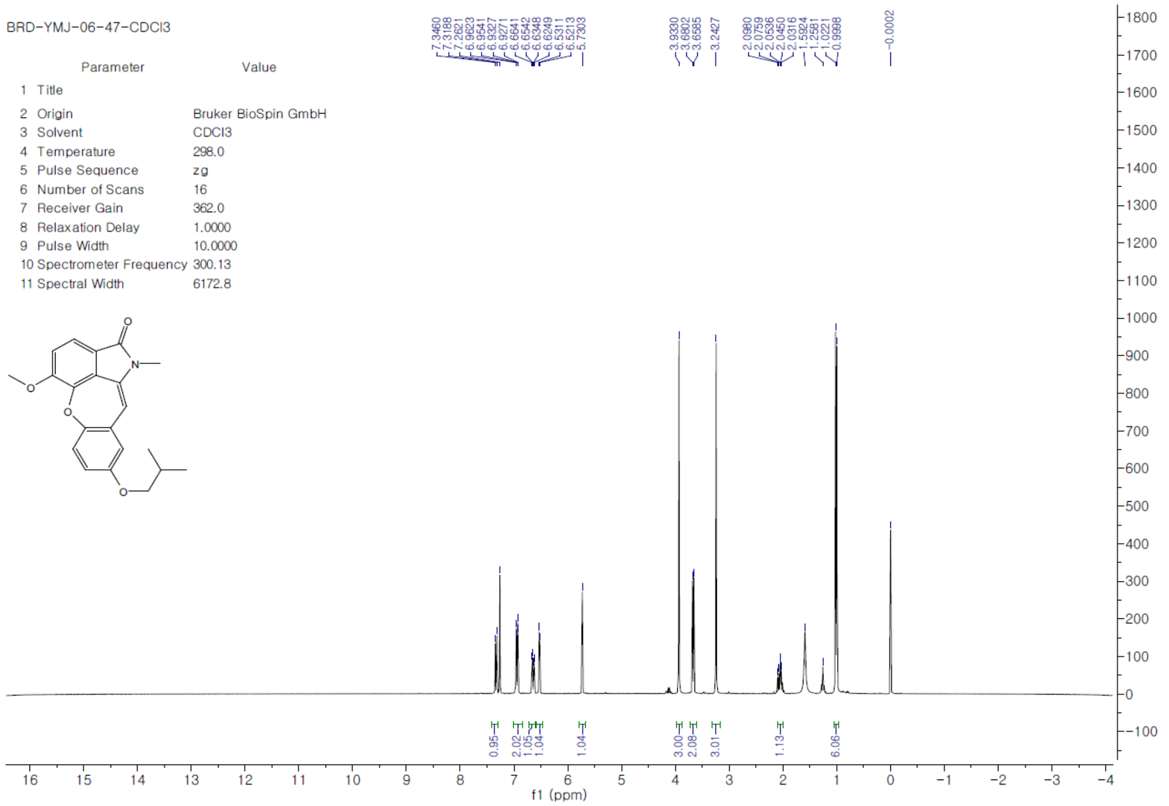

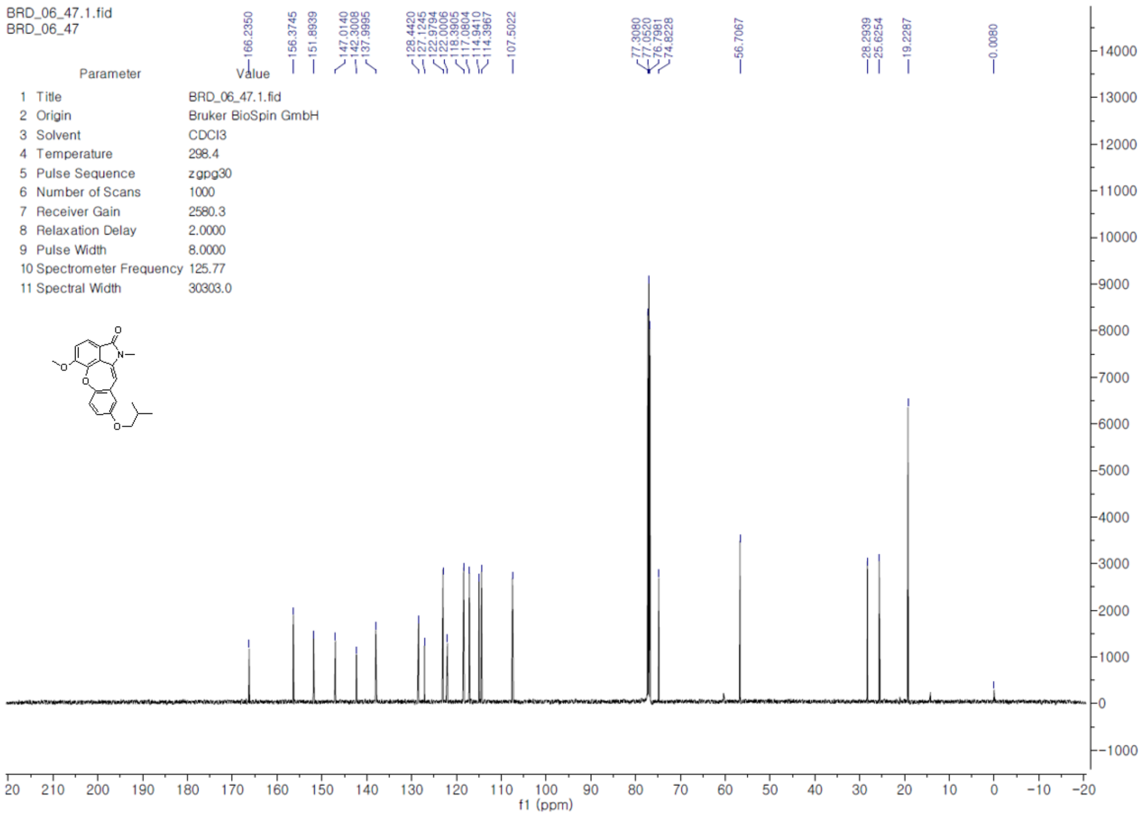

Compound 8bb

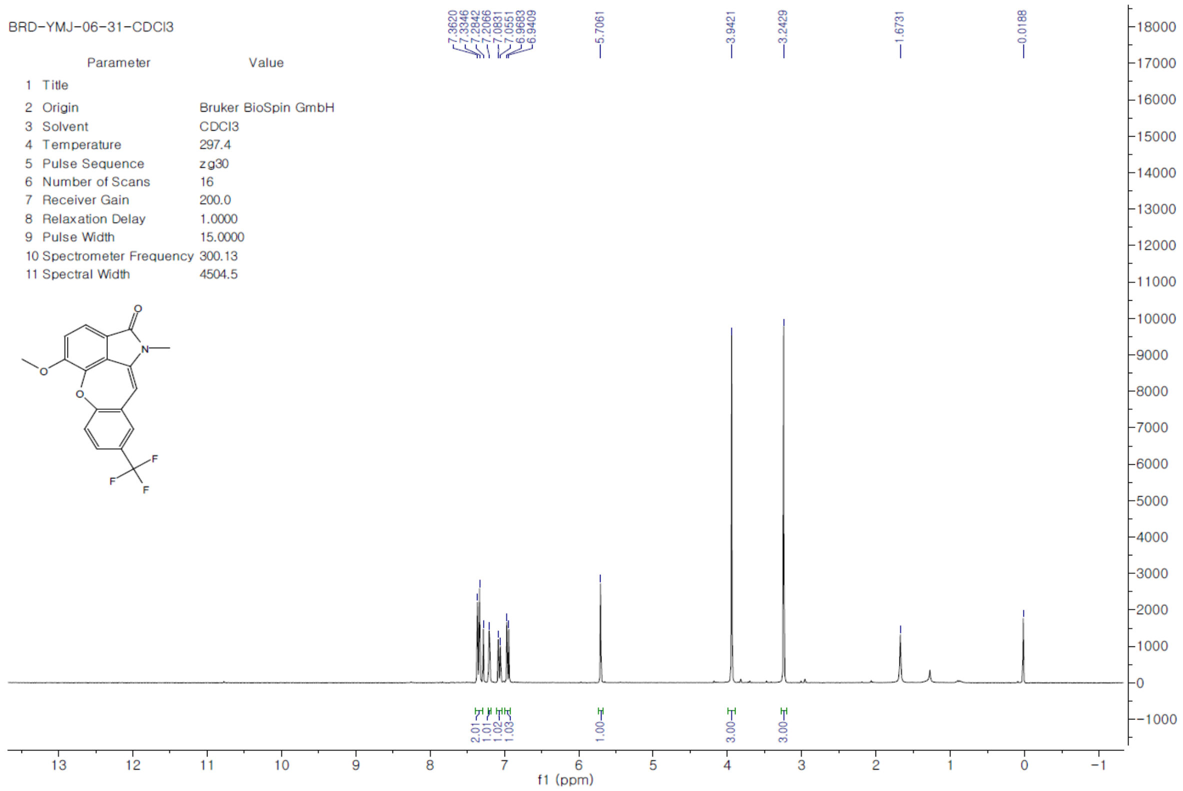

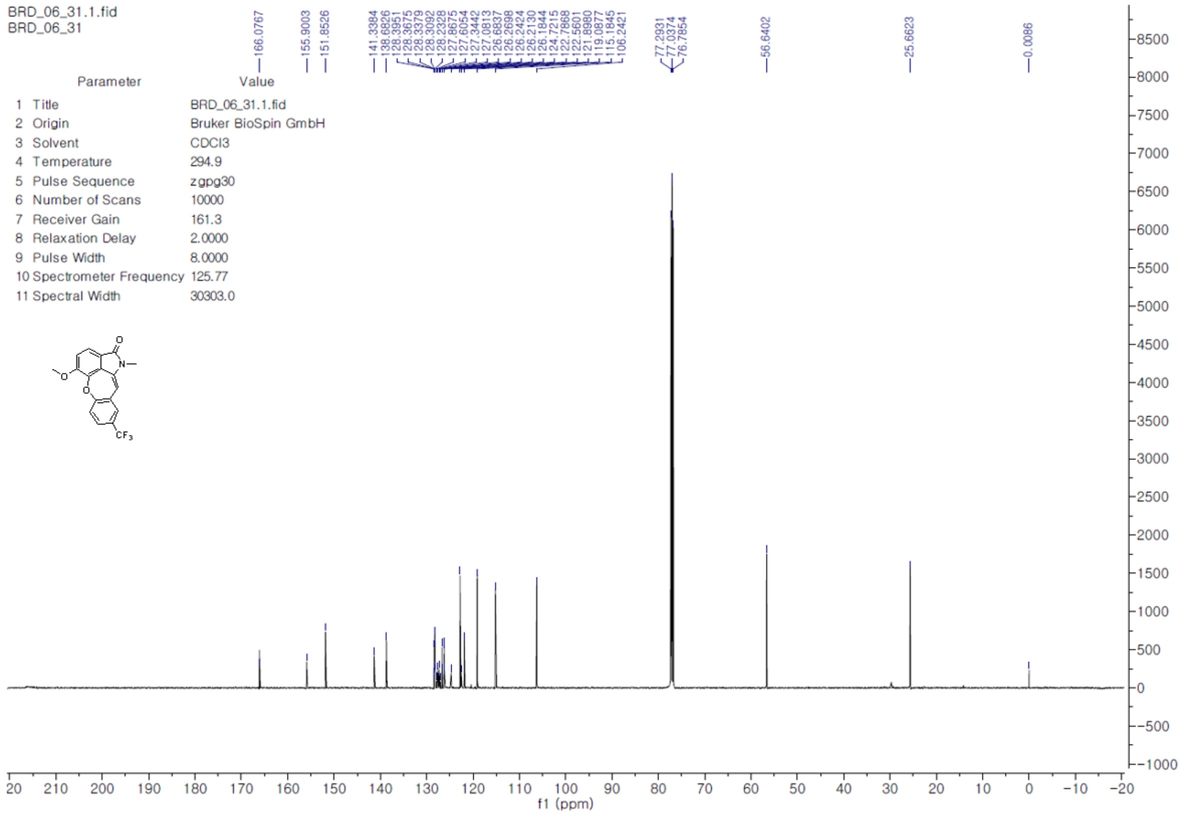

Compound 8bc

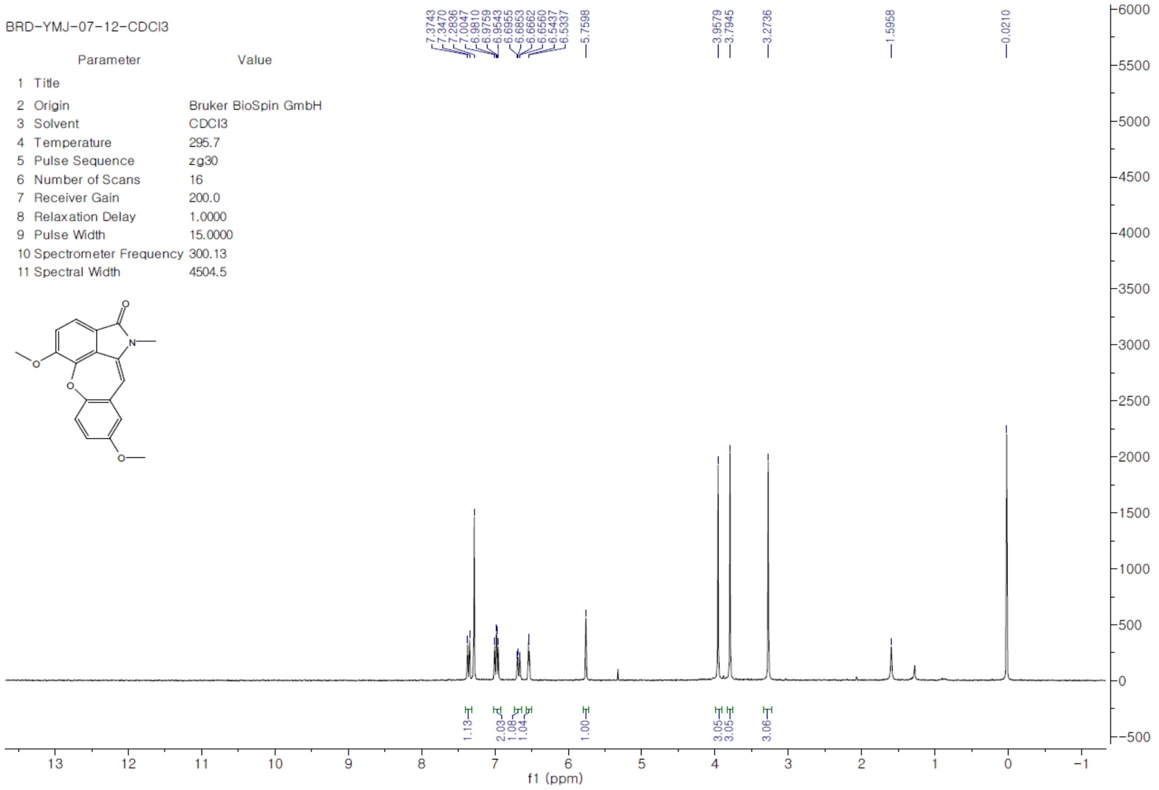

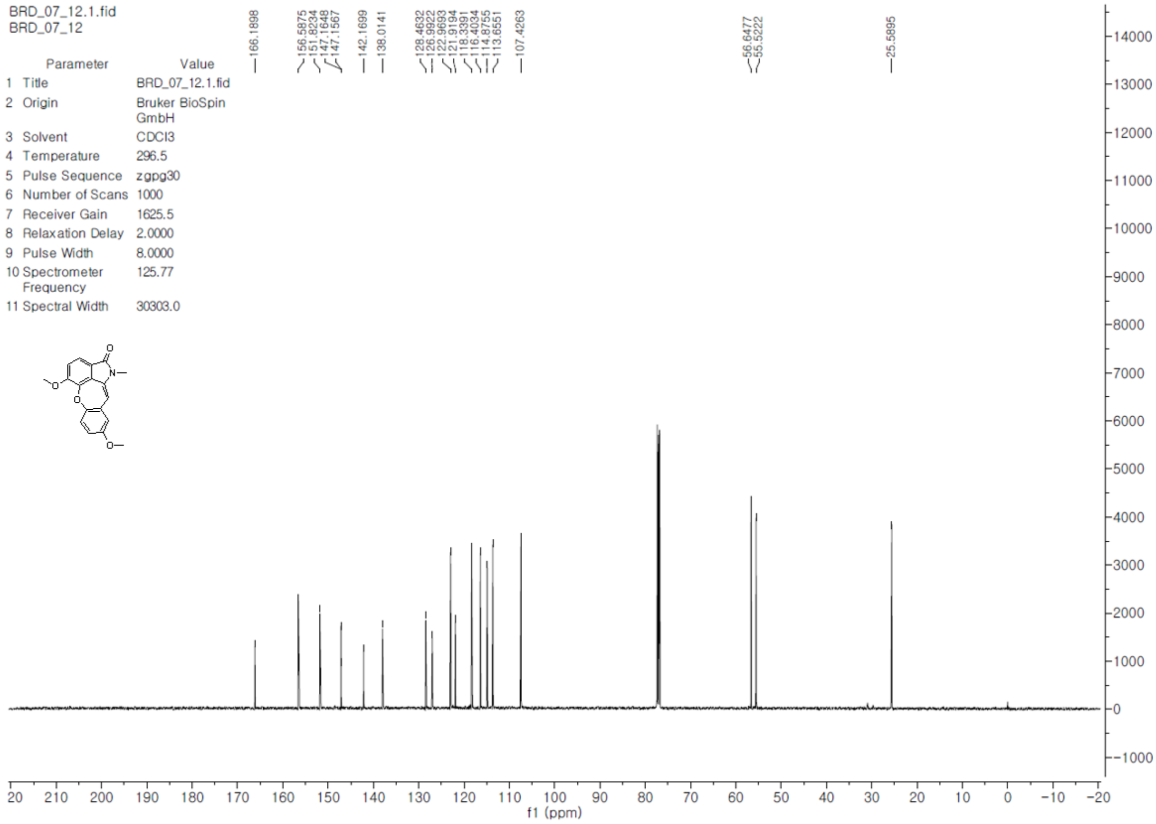

Compound 8bd

BRD-YMJ-07-24-1-CDCl3

	Parameter	Value
1	Title	
2	Origin	Bruker BioSpin GmbH
3	Solvent	CDCl3
4	Temperature	295.8
5	Pulse Sequence	zg30
6	Number of Scans	16
7	Receiver Gain	200.0
8	Relaxation Delay	1.0000
9	Pulse Width	15.0000
10	Spectrometer Frequency	300.13
11	Spectral Width	4504.5

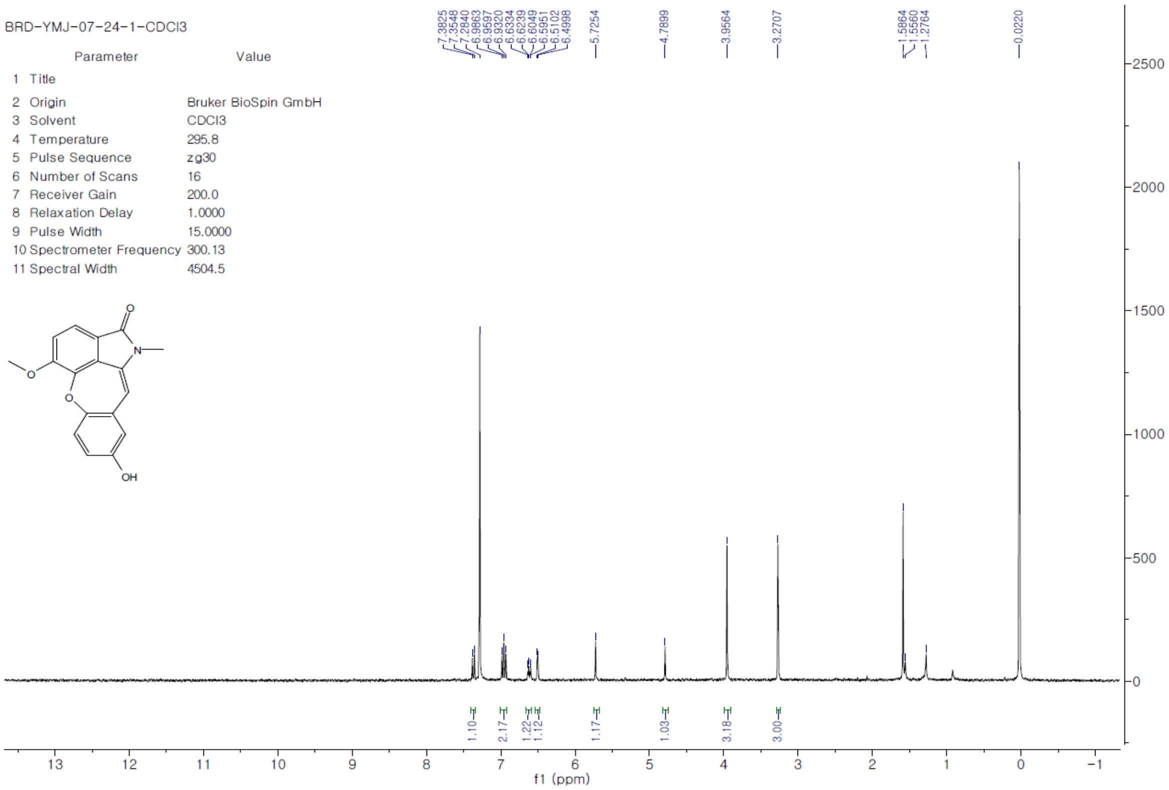

BRD_07_24.3.fid
BRD_07_24

	Parameter	Value
1	Title	BRD_07_24.3.fid
2	Origin	Bruker BioSpin GmbH
3	Solvent	MeOD
4	Temperature	297.4
5	Pulse Sequence	zgpg30
6	Number of Scans	10000
7	Receiver Gain	2580.3
8	Relaxation Delay	2.0000
9	Pulse Width	8.0000
10	Spectrometer Frequency	125.77
11	Spectral Width	30303.0

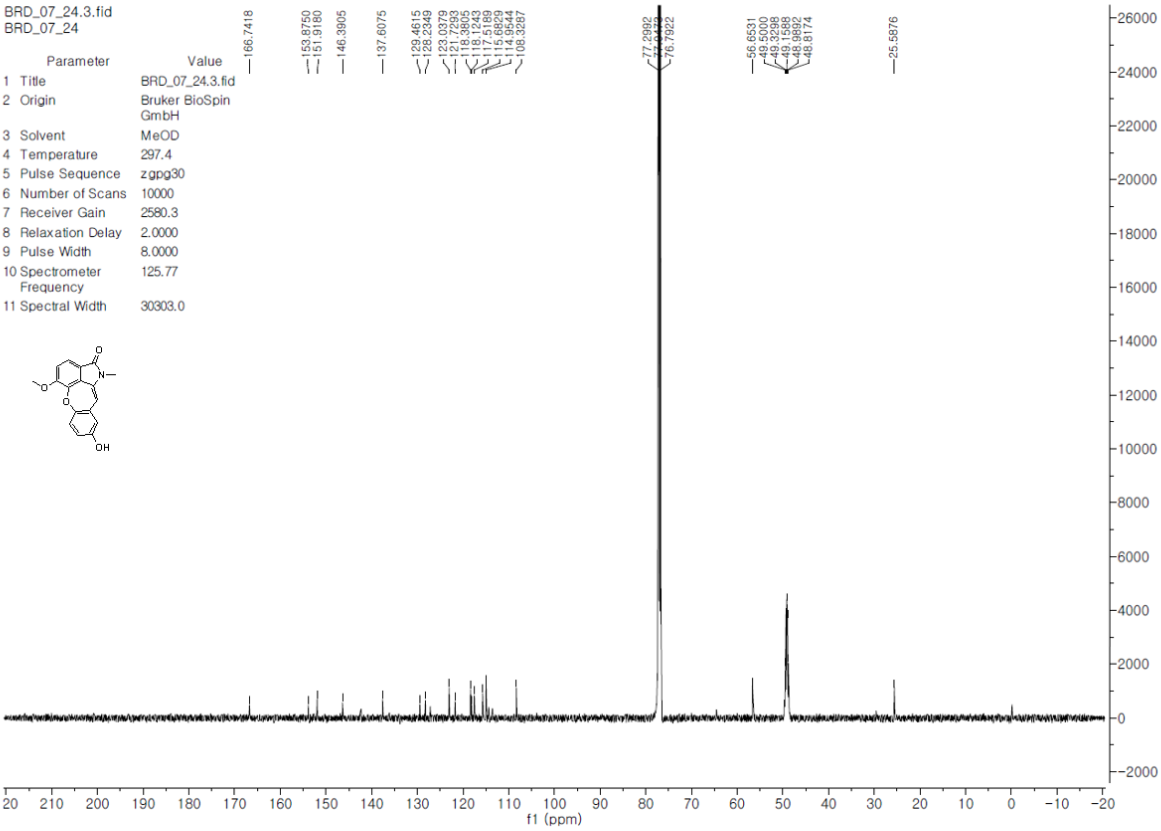

Compound 8be

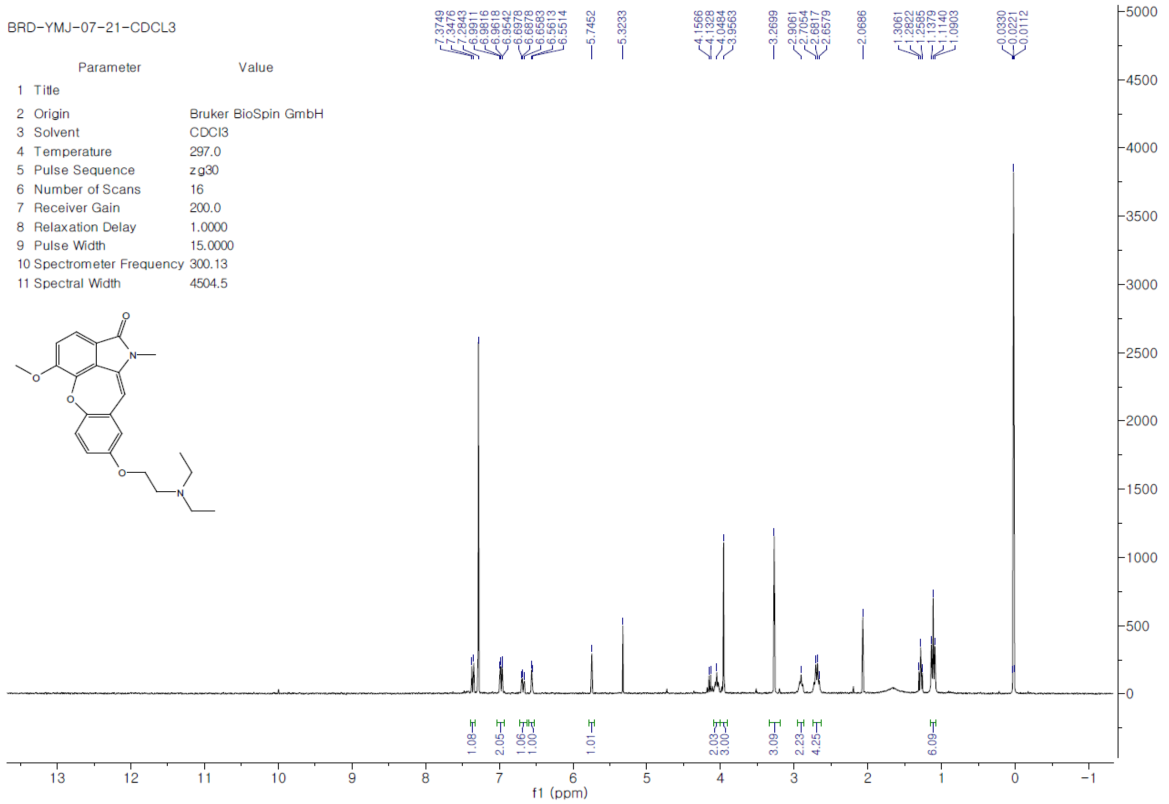

	Parameter	Value
1	Title	
2	Origin	Bruker BioSpin GmbH
3	Solvent	CDCl3
4	Temperature	297.0
5	Pulse Sequence	zg30
6	Number of Scans	16
7	Receiver Gain	200.0
8	Relaxation Delay	1.0000
9	Pulse Width	15.0000
10	Spectrometer Frequency	300.13
11	Spectral Width	4504.5

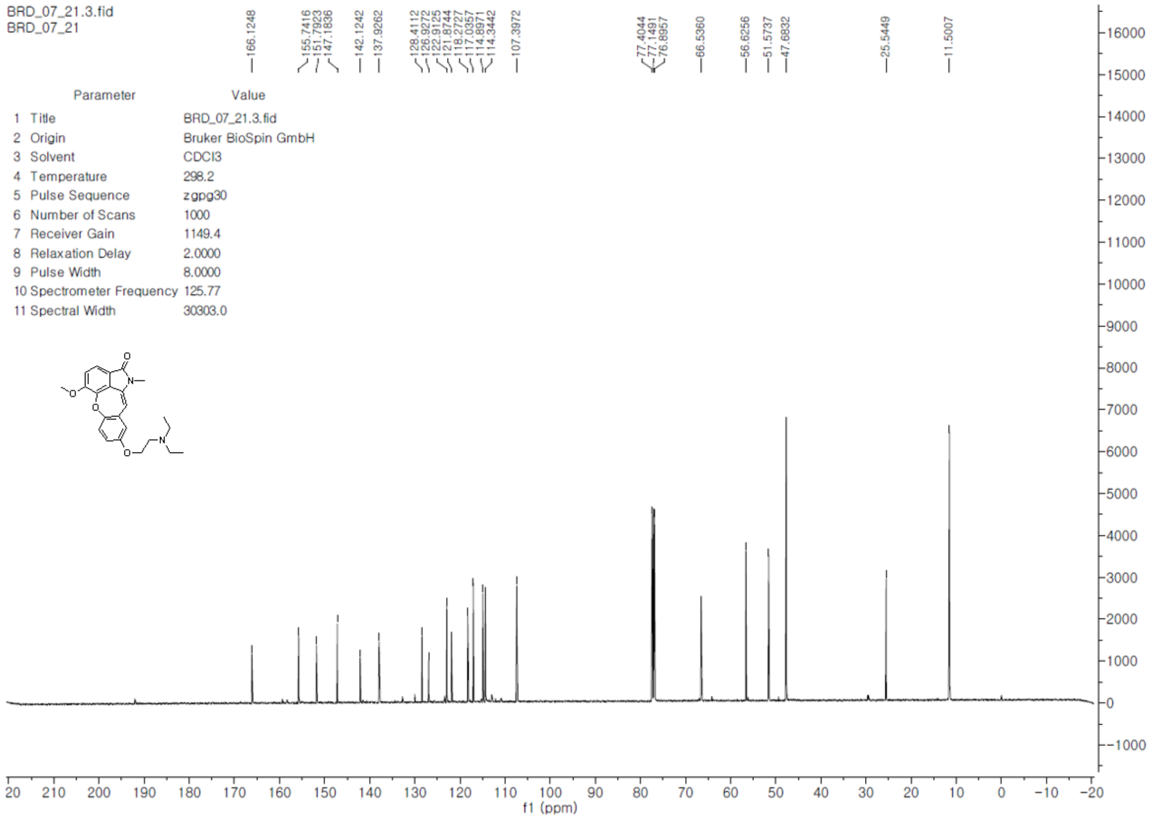

	Parameter	Value
1	Title	BRD_07_21.3.fid
2	Origin	Bruker BioSpin GmbH
3	Solvent	CDCl3
4	Temperature	298.2
5	Pulse Sequence	zgpg30
6	Number of Scans	1000
7	Receiver Gain	1149.4
8	Relaxation Delay	2.0000
9	Pulse Width	8.0000
10	Spectrometer Frequency	125.77
11	Spectral Width	30303.0

Compound 8bf

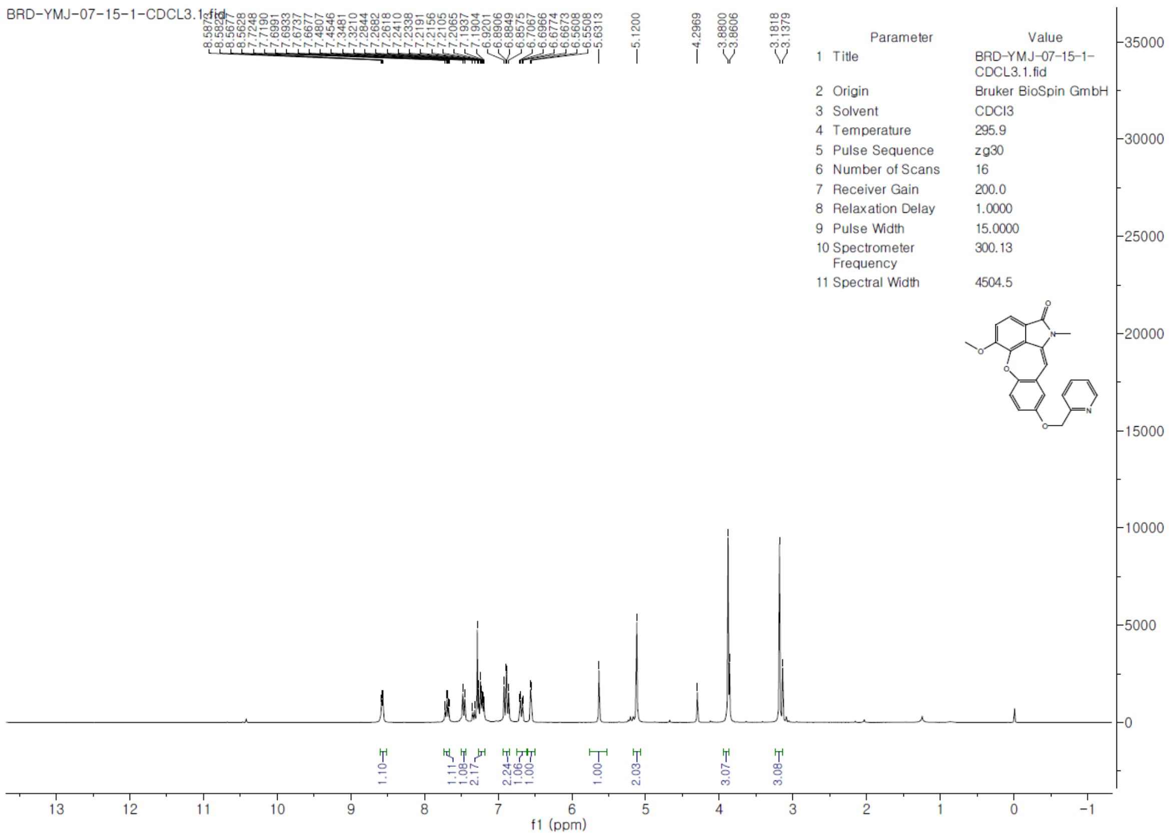

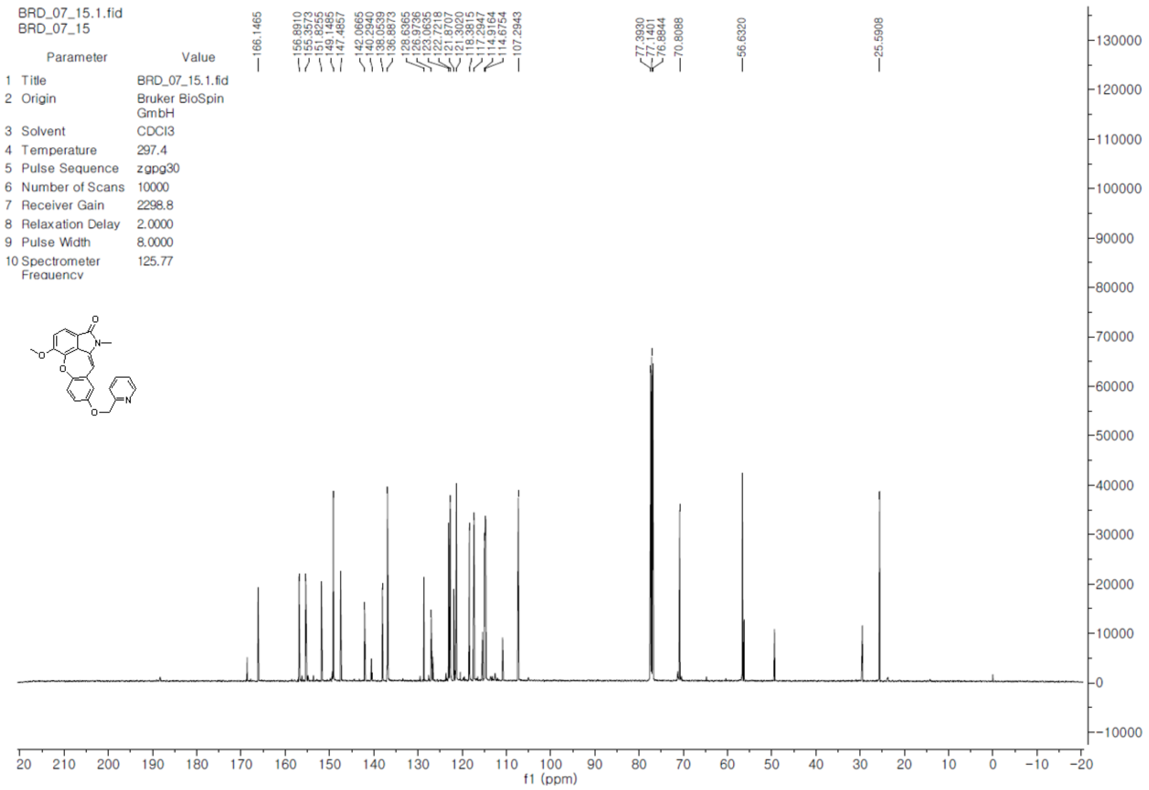

Compound 8bg

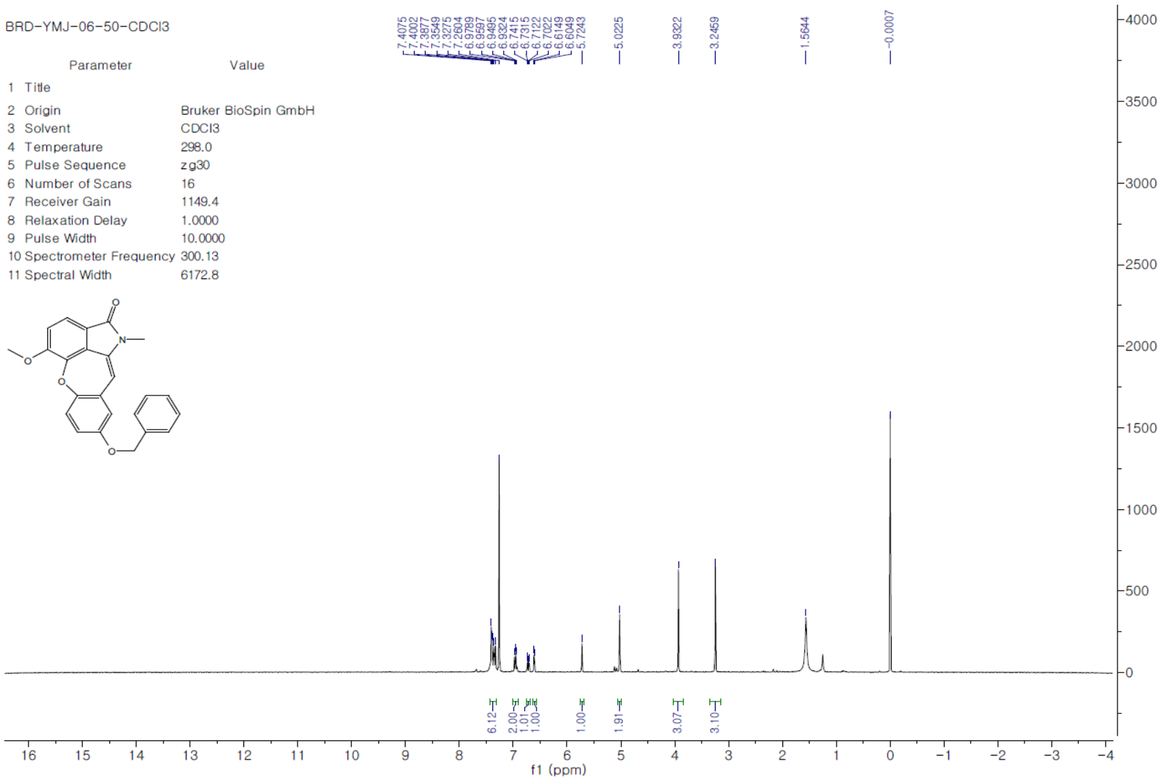

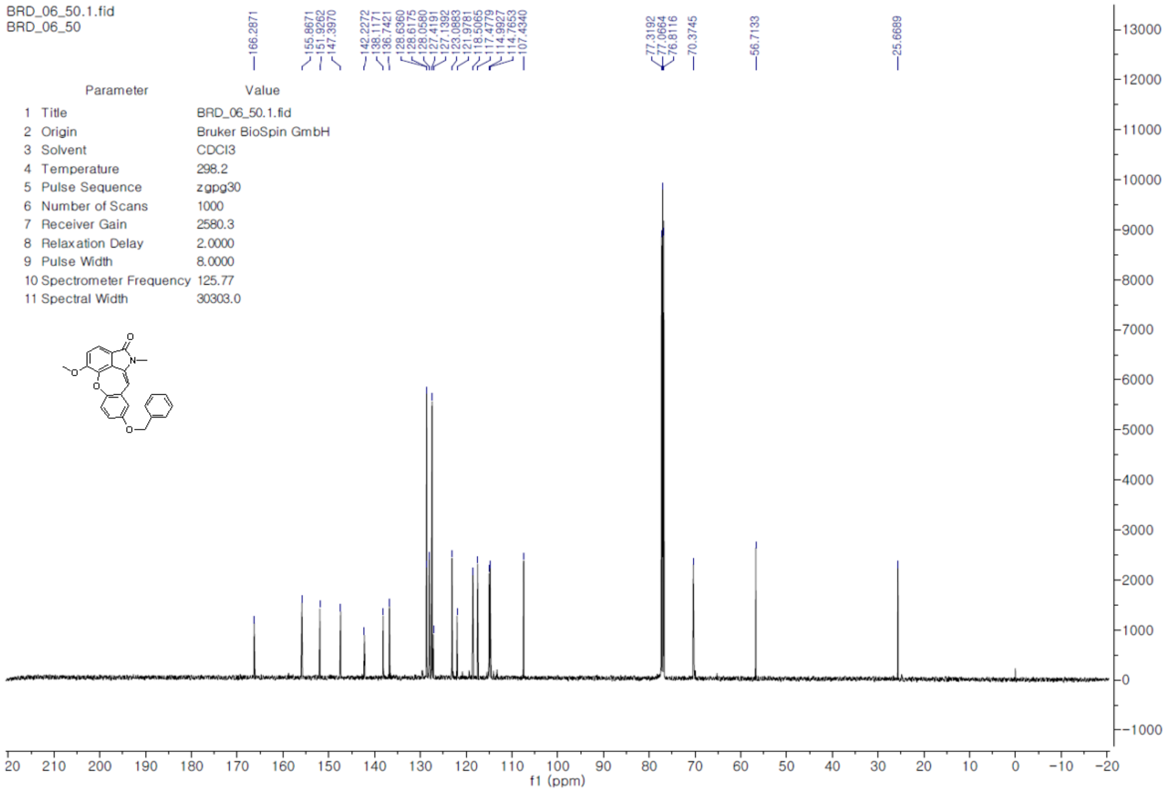

Compound 8bh

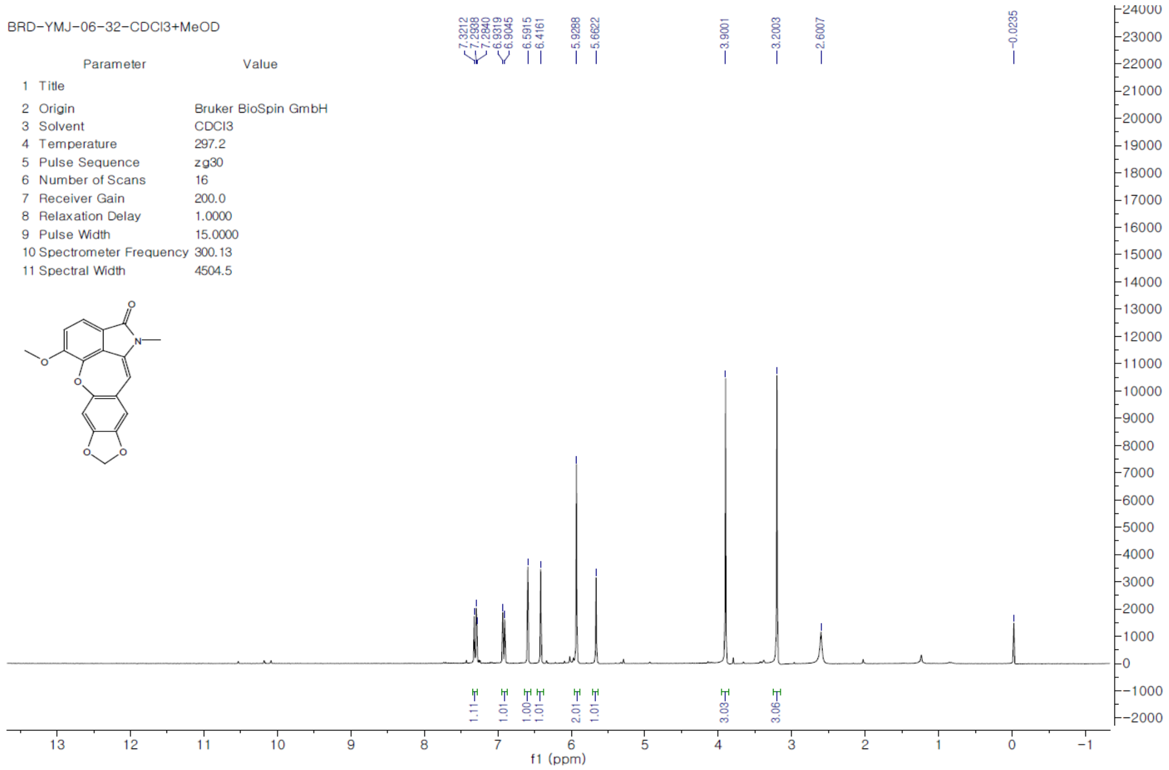

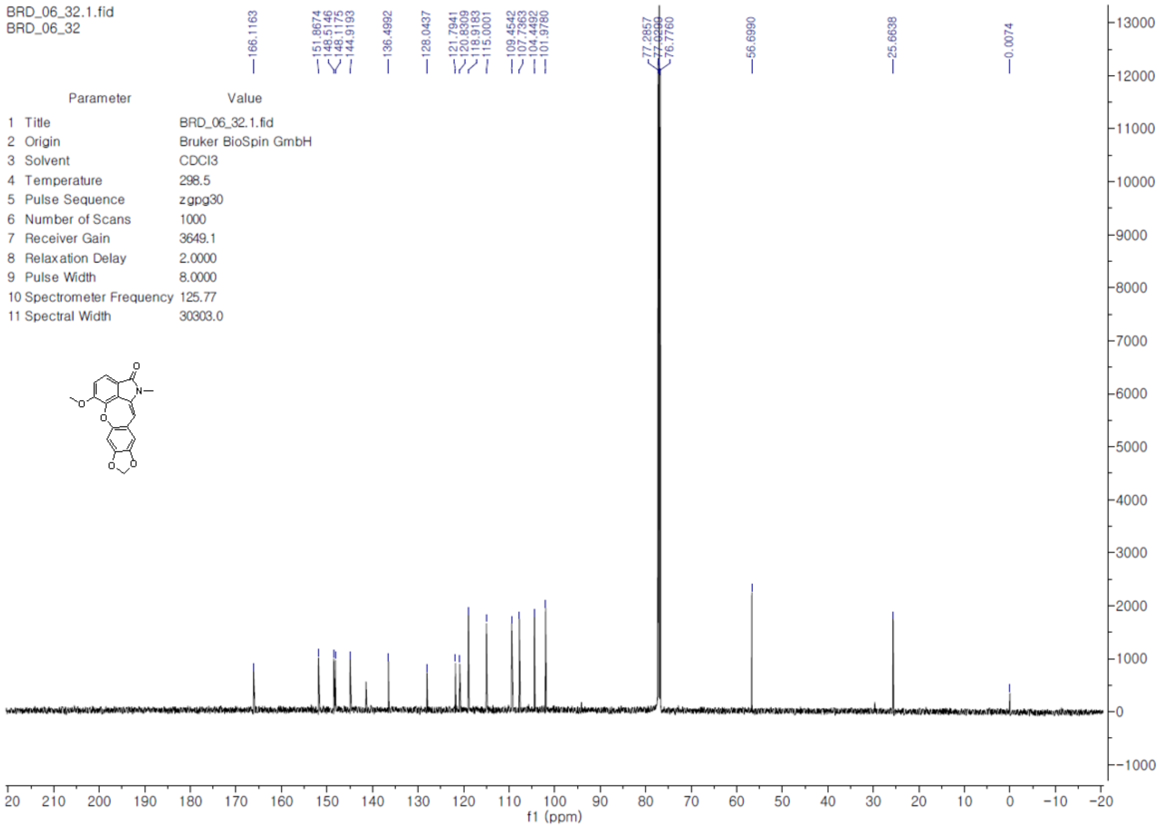

Compound 8bi

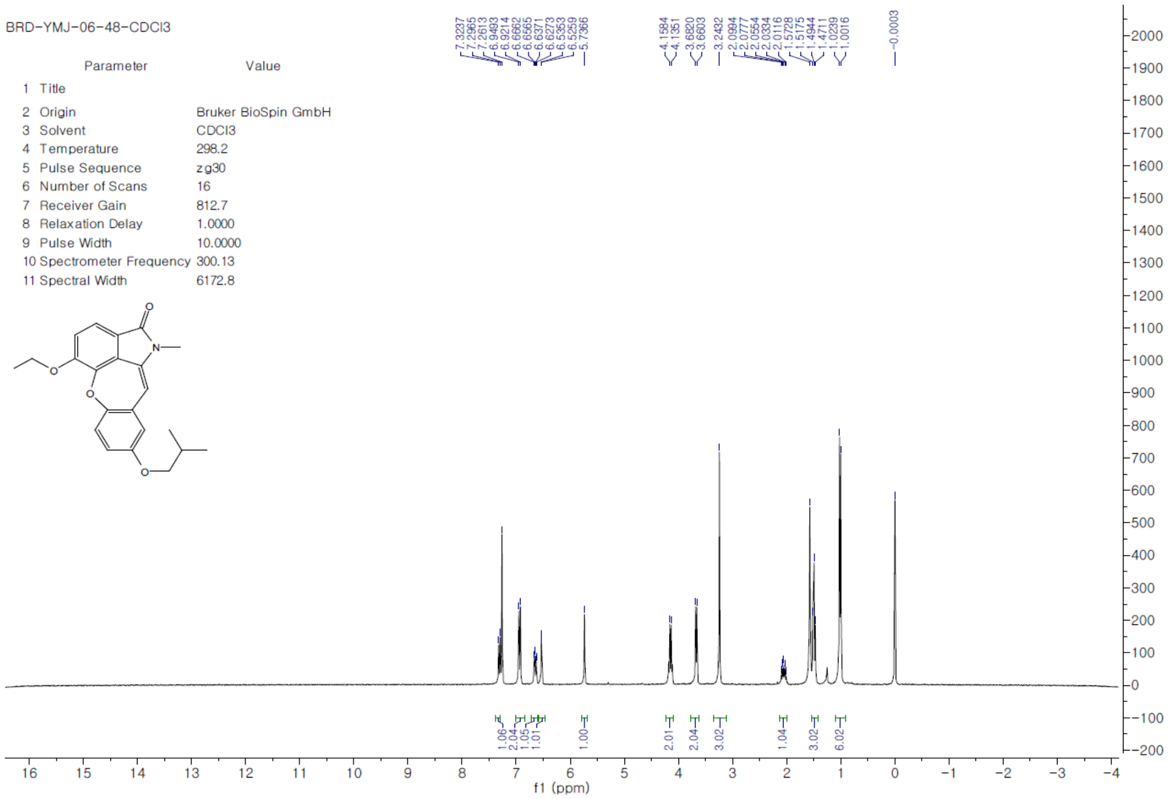

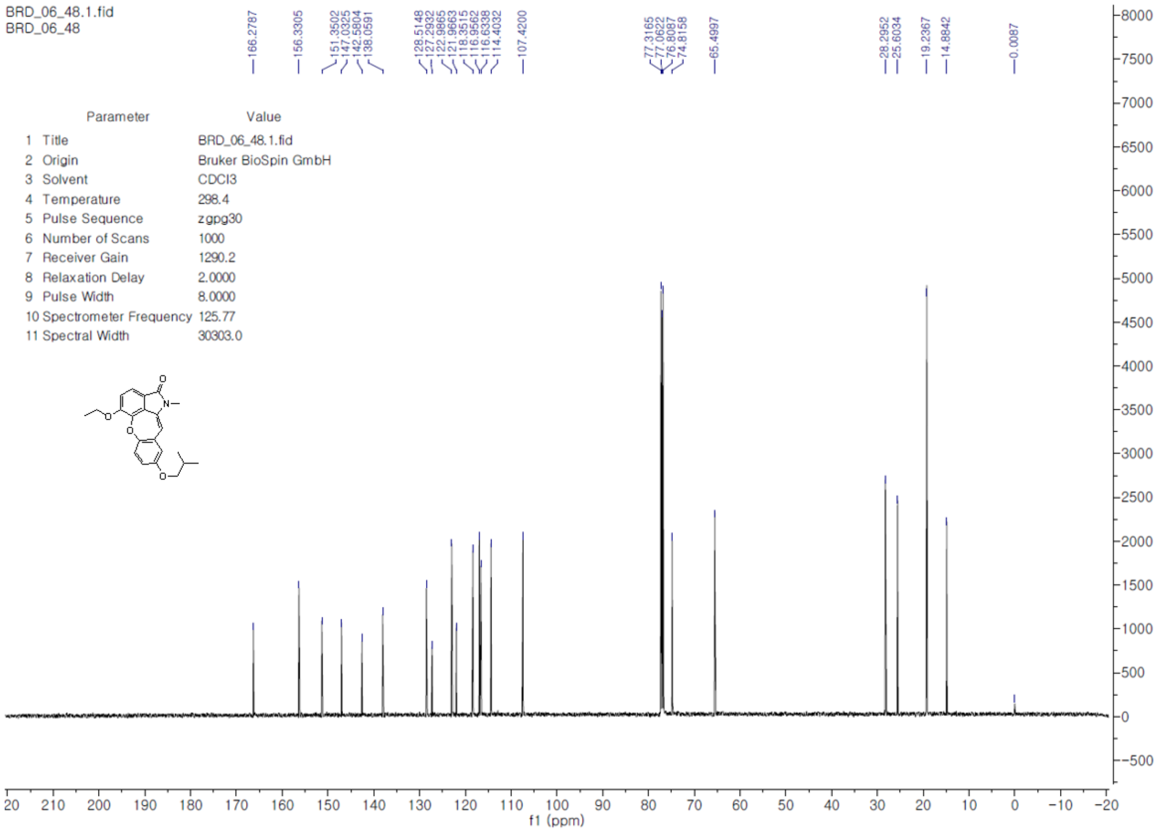

Compound 8bj

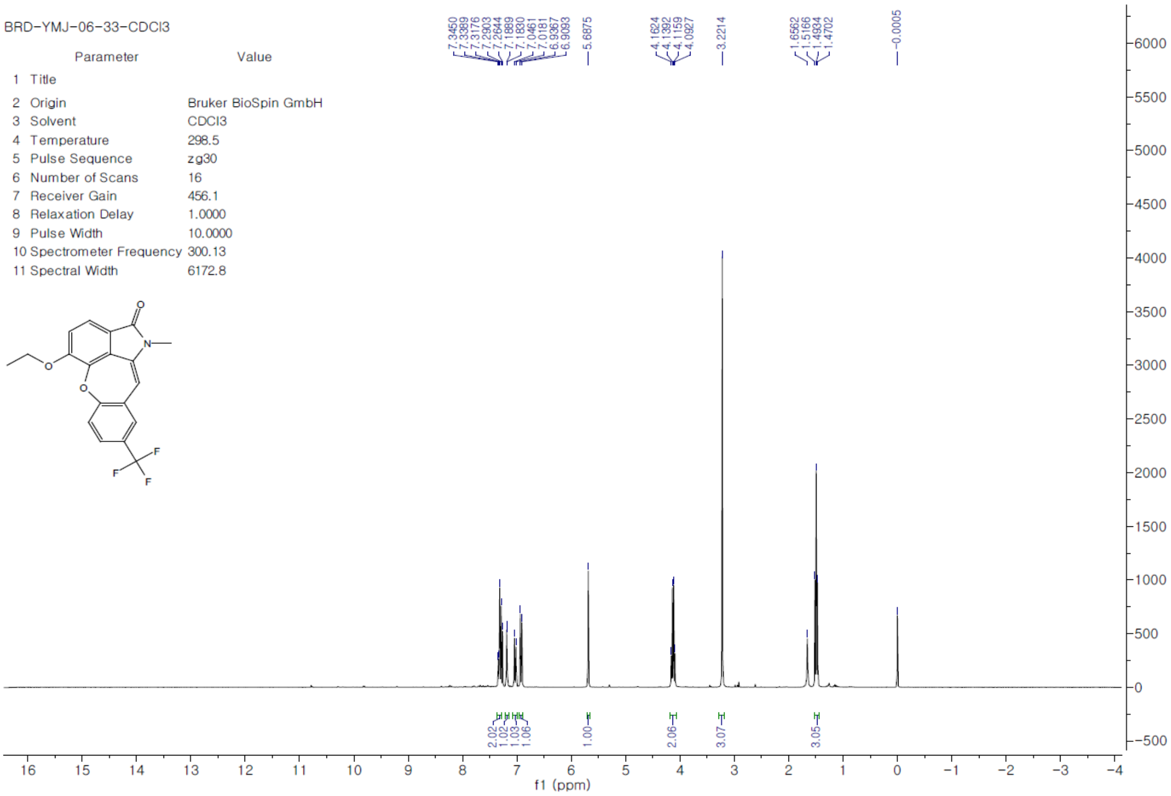

	Parameter	Value
1	Title	
2	Origin	Bruker BioSpin GmbH
3	Solvent	CDCl3
4	Temperature	298.5
5	Pulse Sequence	zg30
6	Number of Scans	16
7	Receiver Gain	456.1
8	Relaxation Delay	1.0000
9	Pulse Width	10.0000
10	Spectrometer Frequency	300.13
11	Spectral Width	6172.8

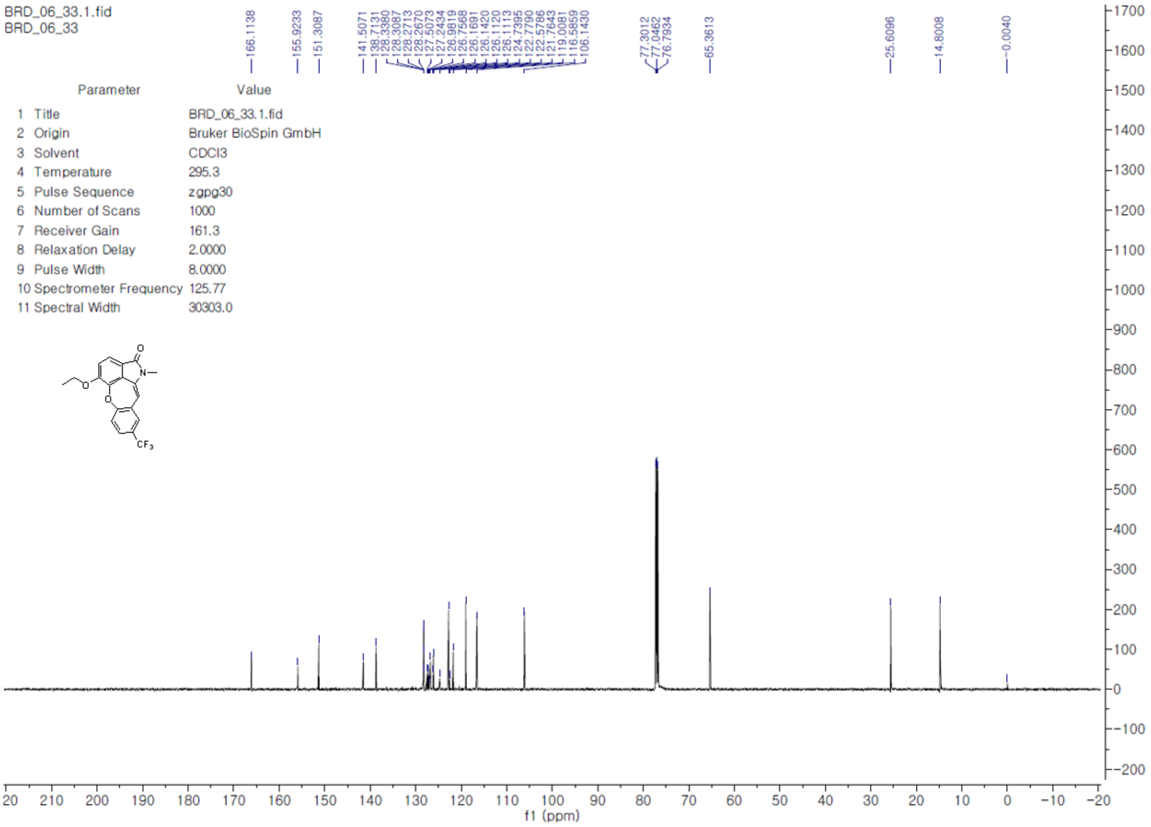

	Parameter	Value
1	Title	BRD_06_33.1.fid
2	Origin	Bruker BioSpin GmbH
3	Solvent	CDCl3
4	Temperature	295.3
5	Pulse Sequence	zgpg30
6	Number of Scans	1000
7	Receiver Gain	161.3
8	Relaxation Delay	2.0000
9	Pulse Width	8.0000
10	Spectrometer Frequency	125.77
11	Spectral Width	30303.0

Compound 8bk

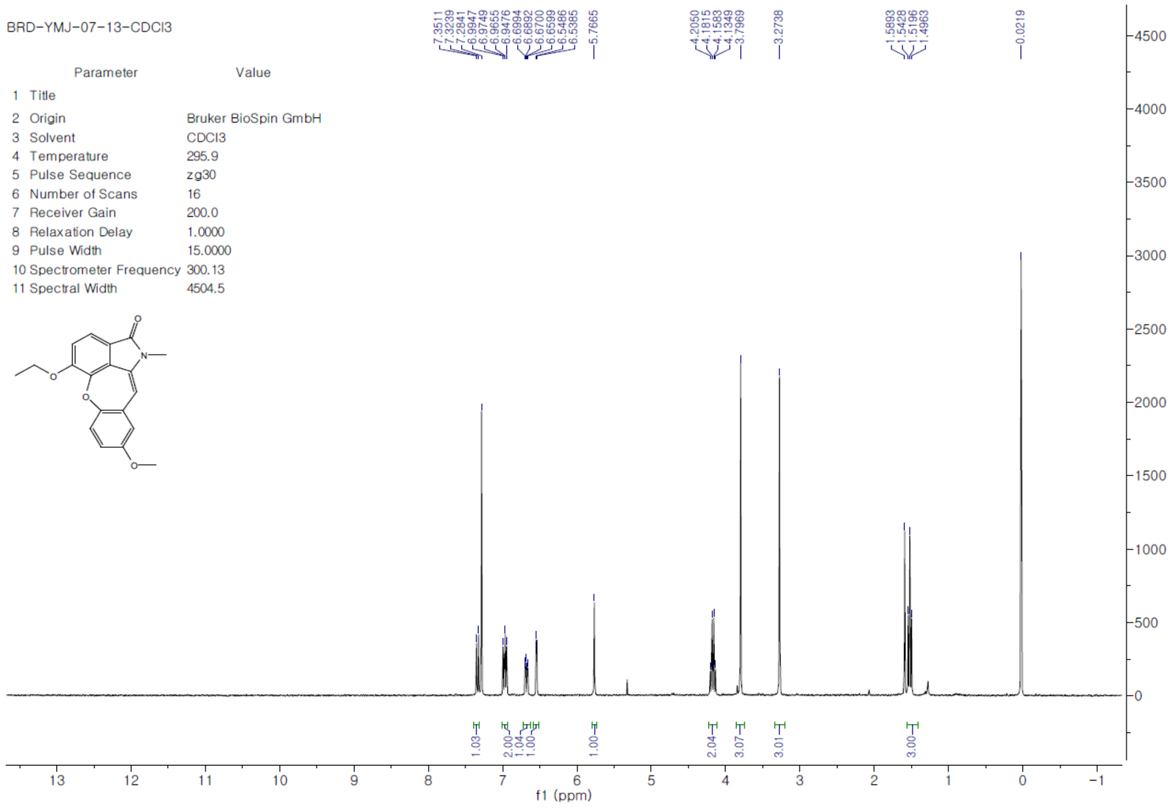

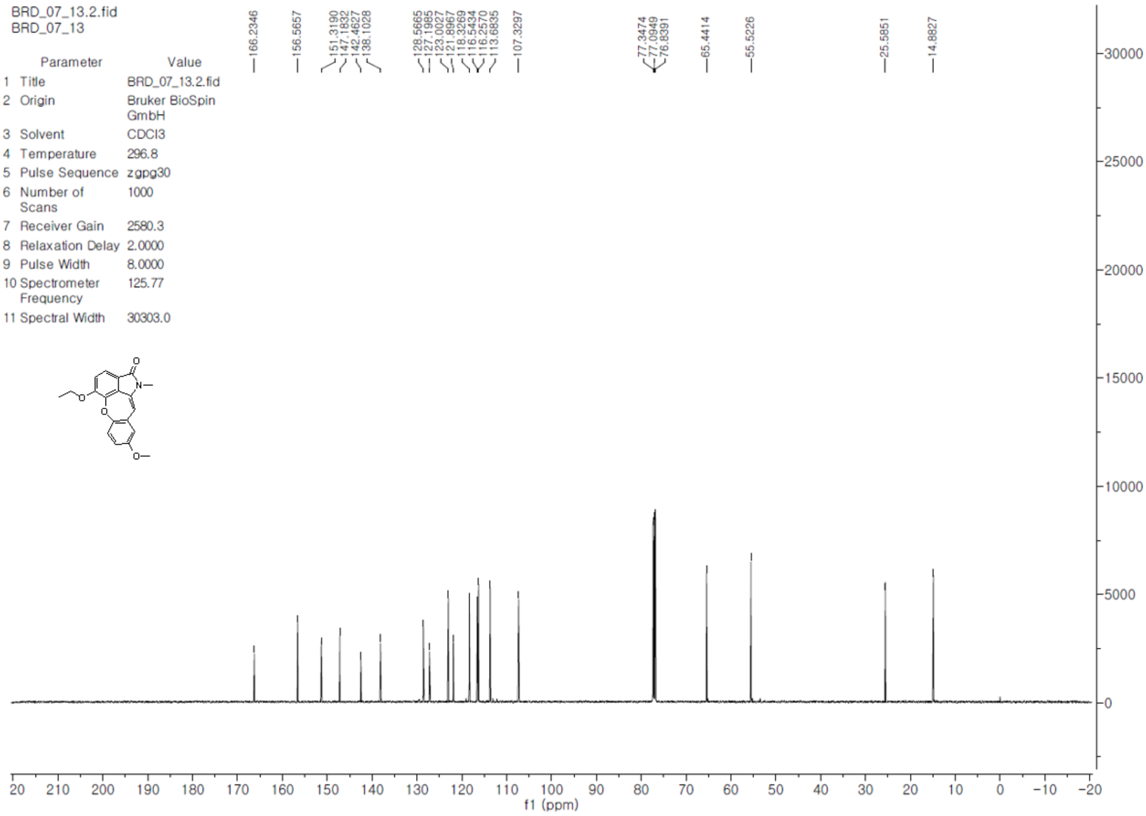

Compound 8bl

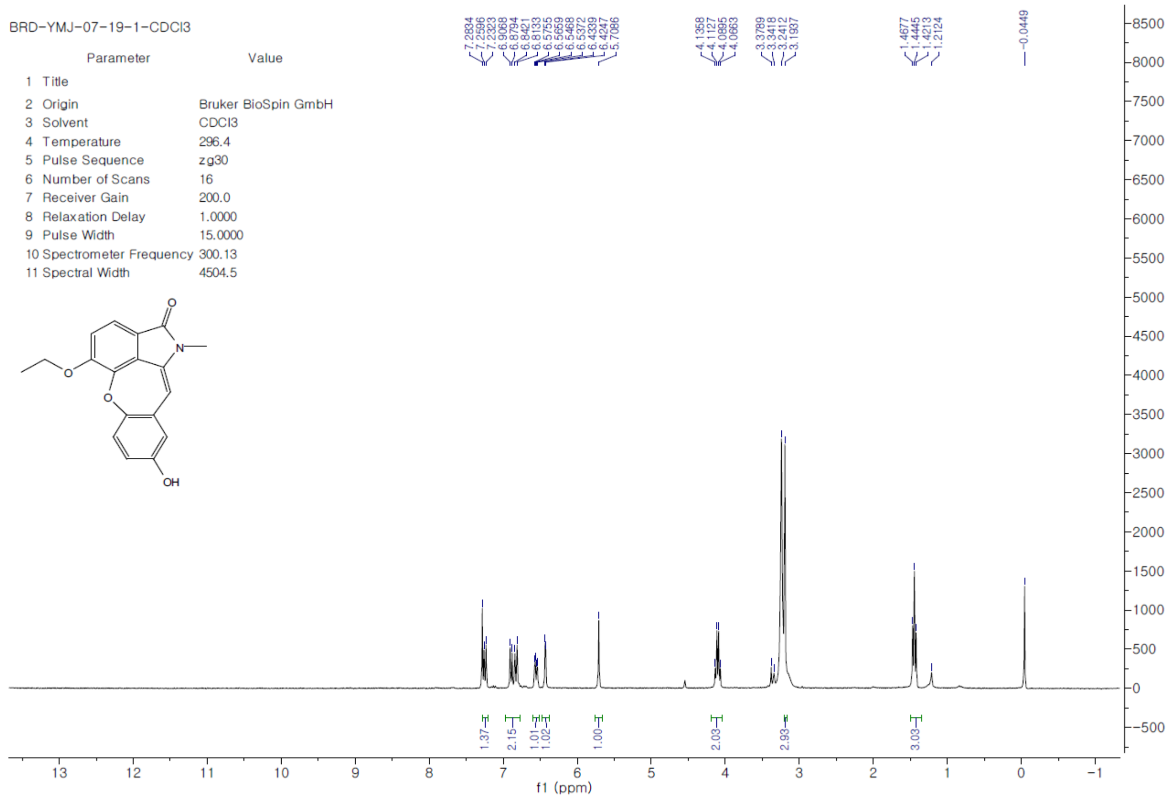

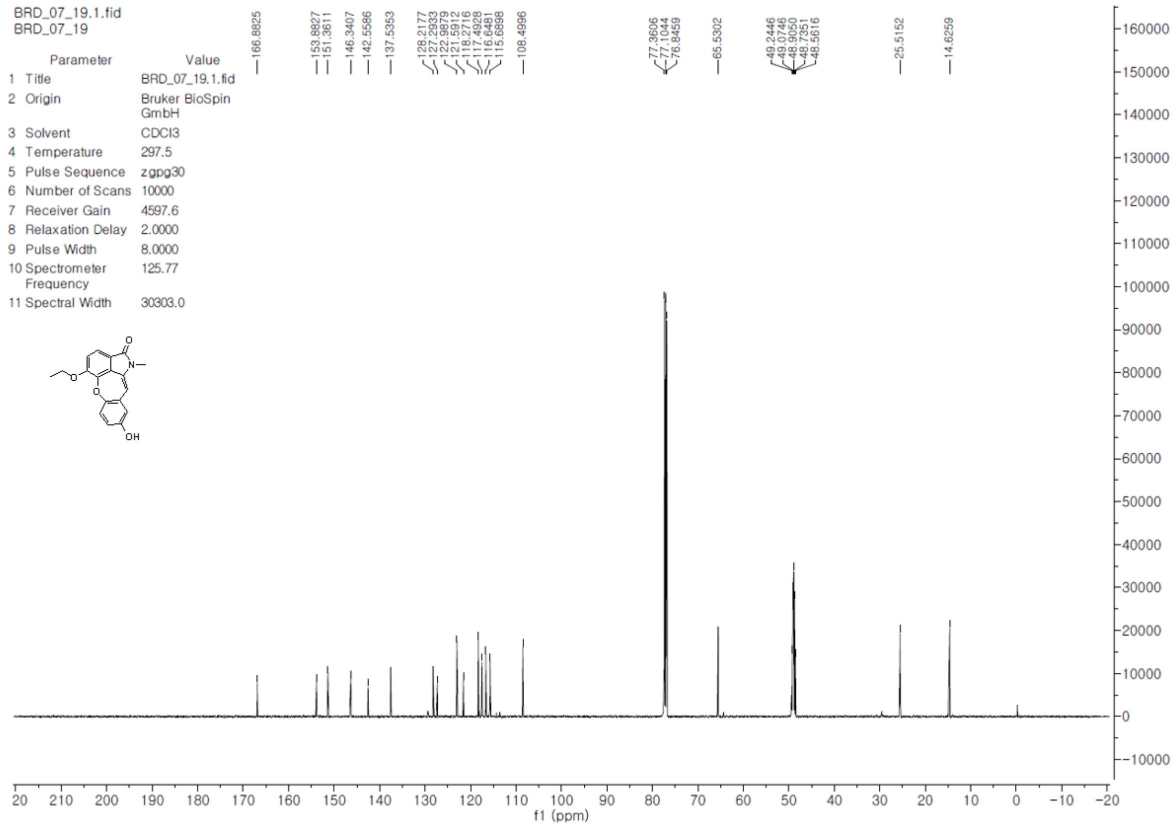

Compound 8bm

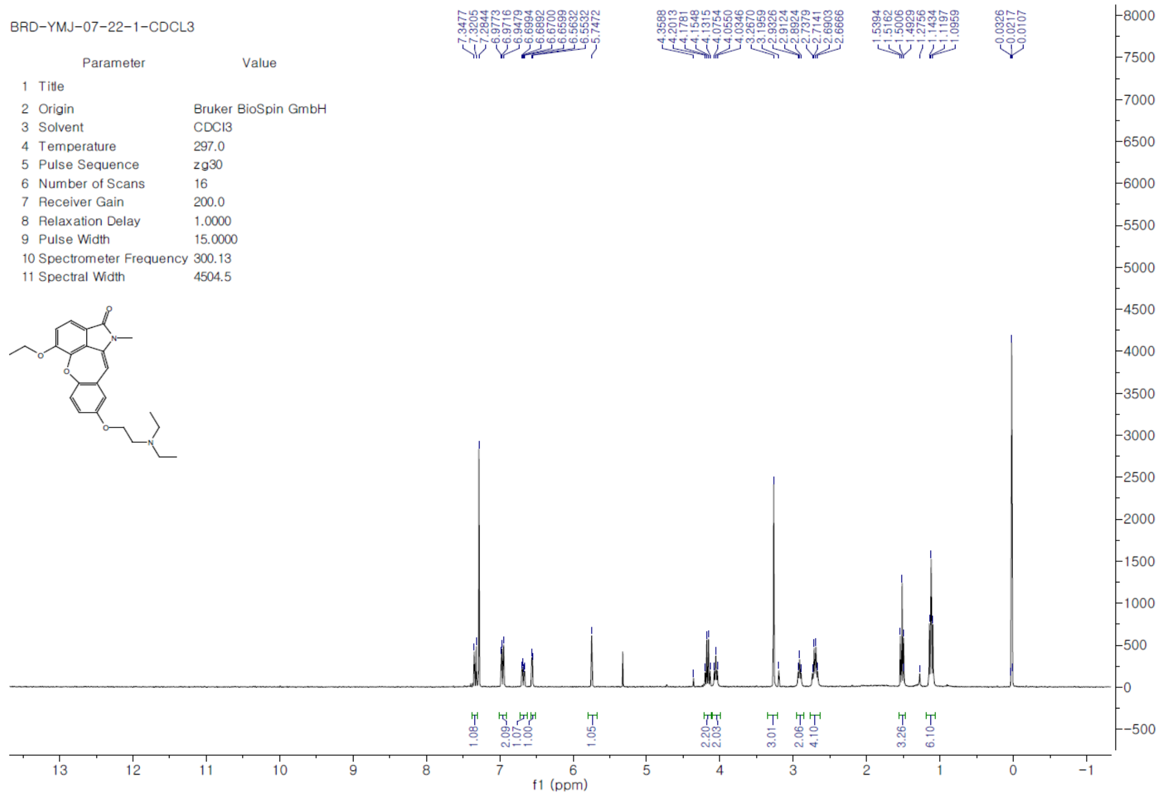

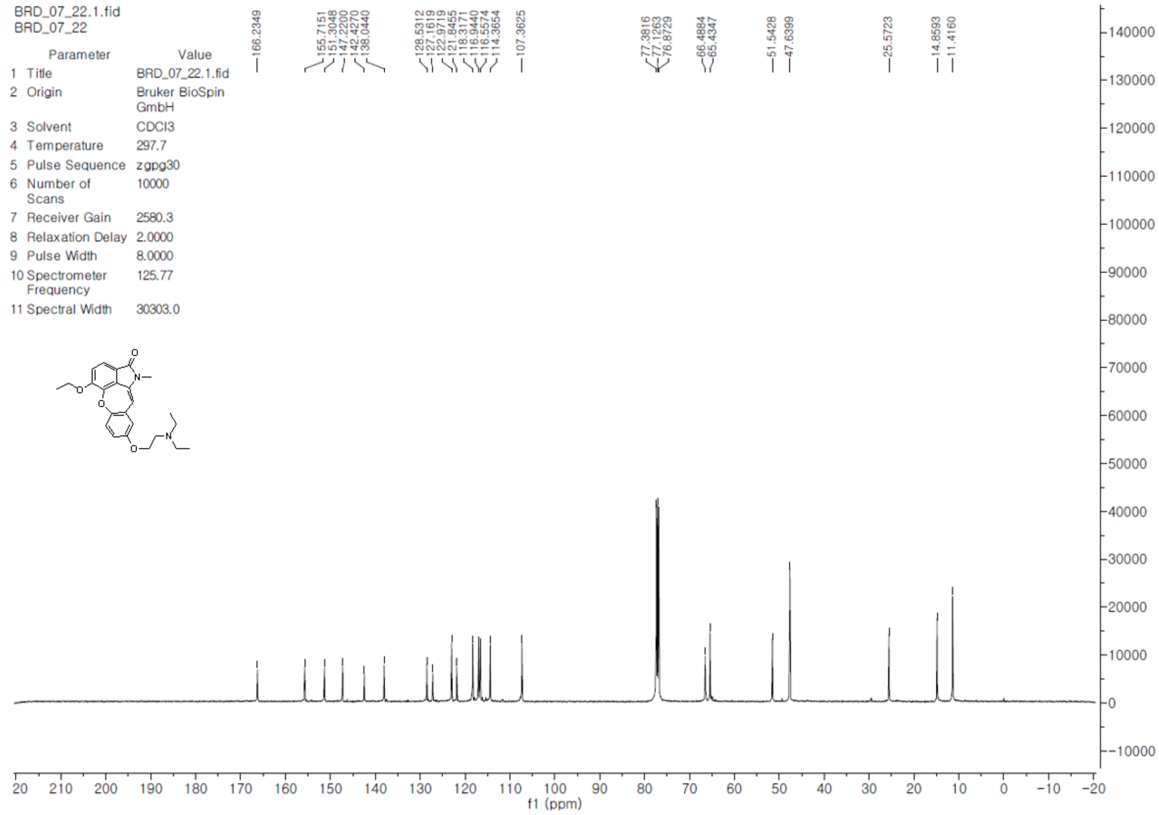

Compound 8bn

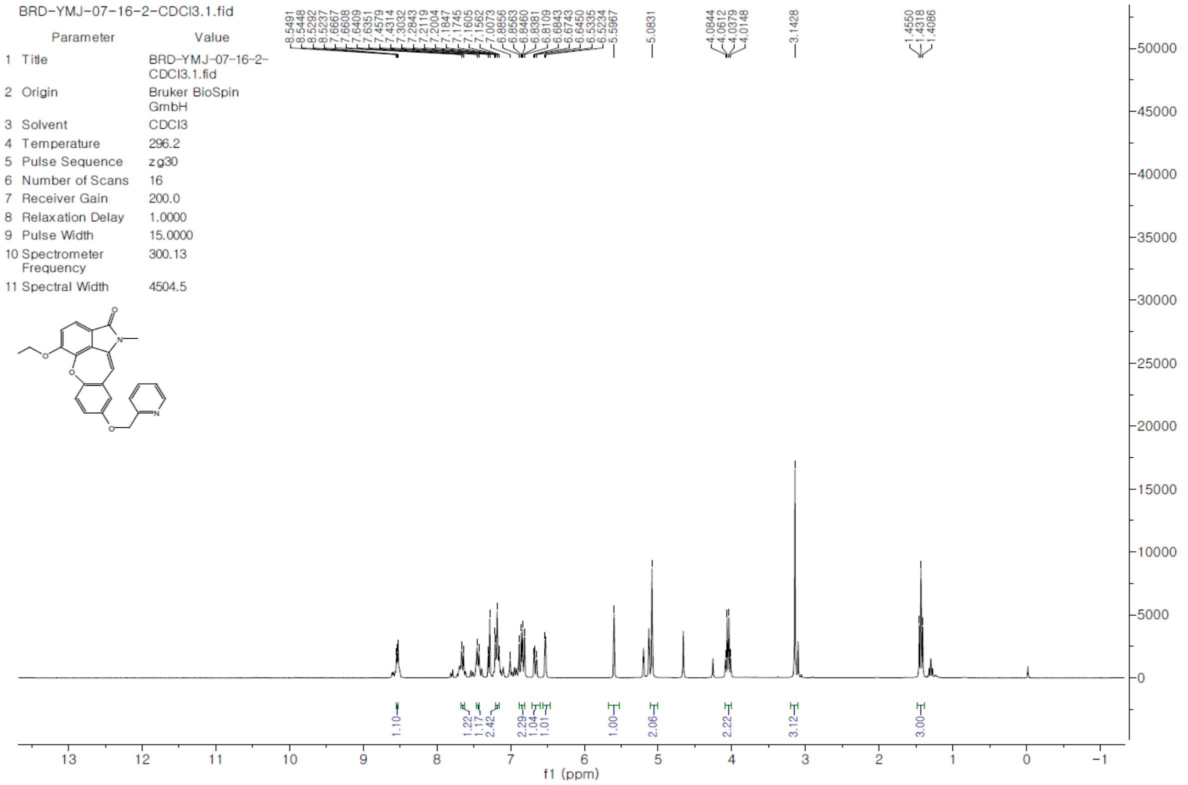

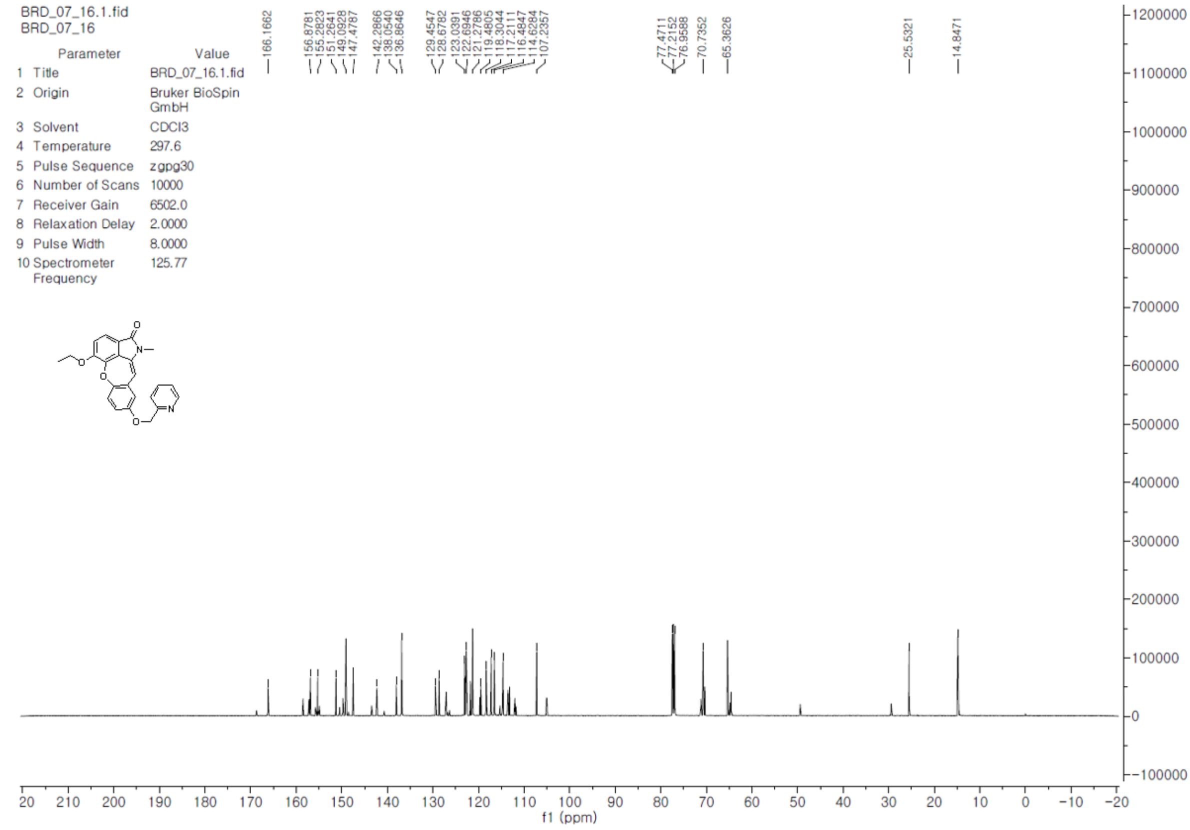

Compound 8mo

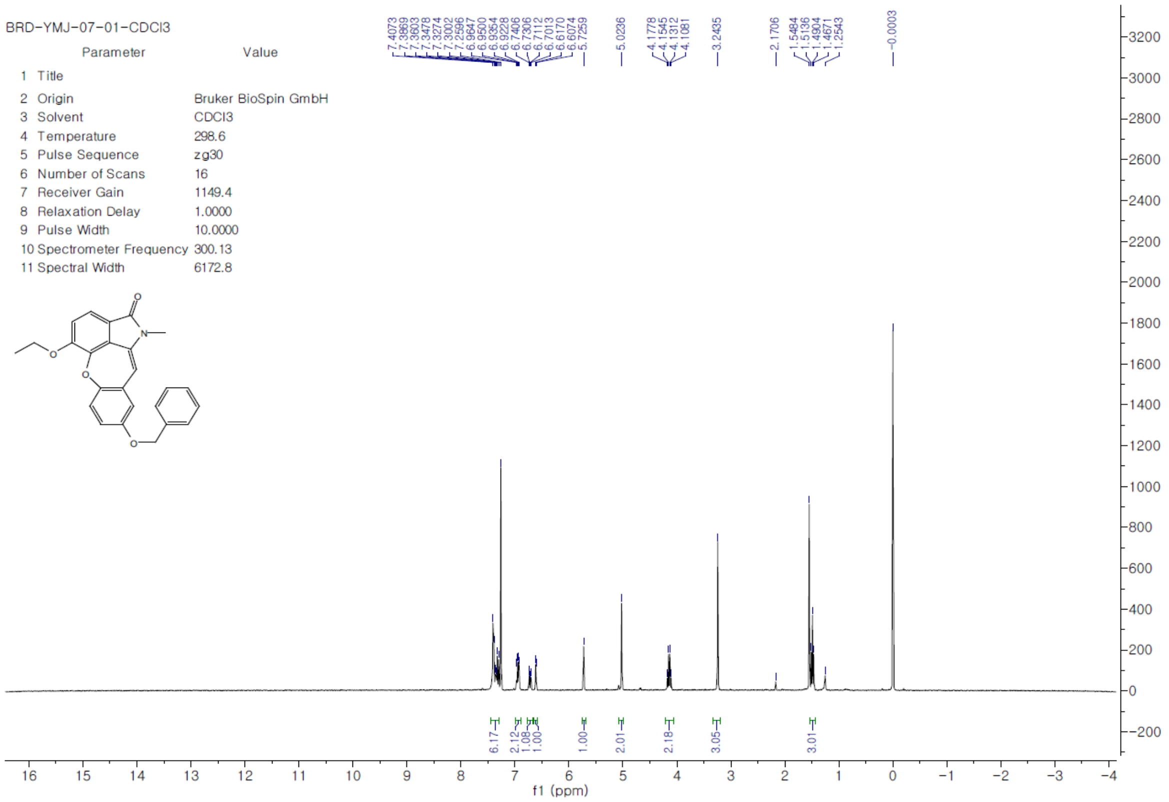

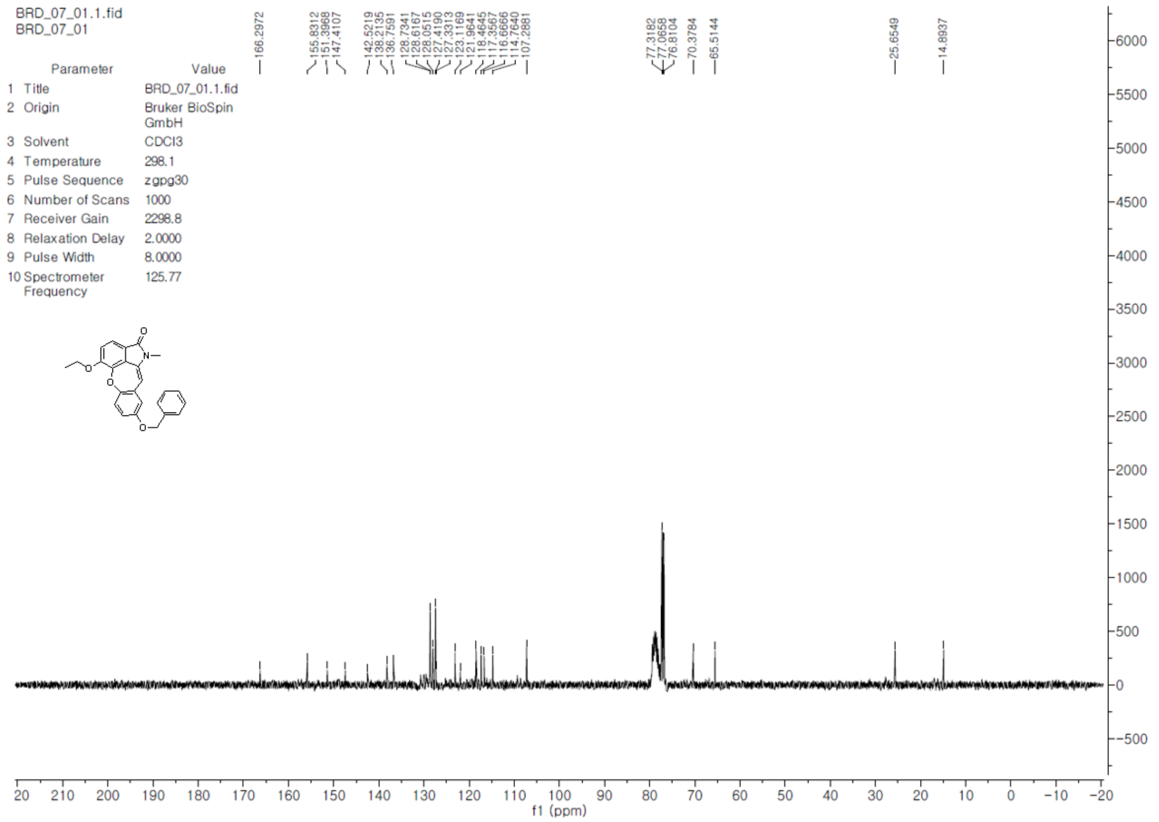

Compound 8bp

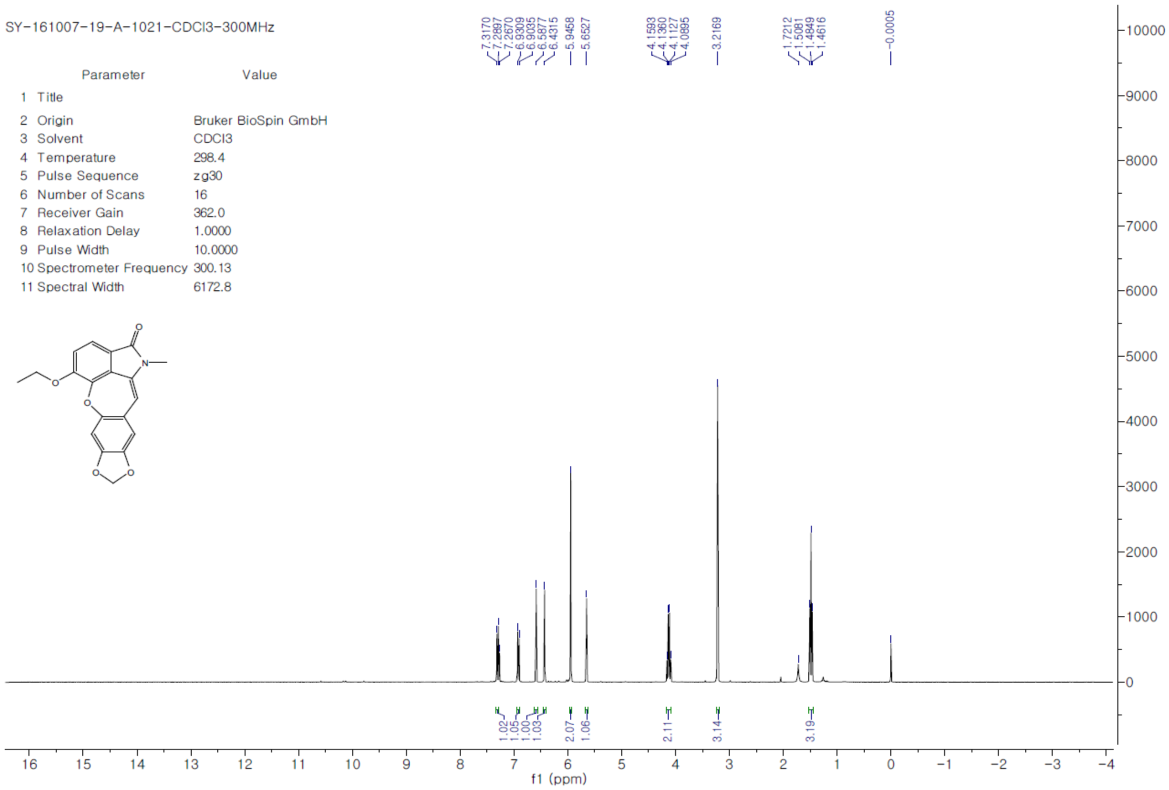

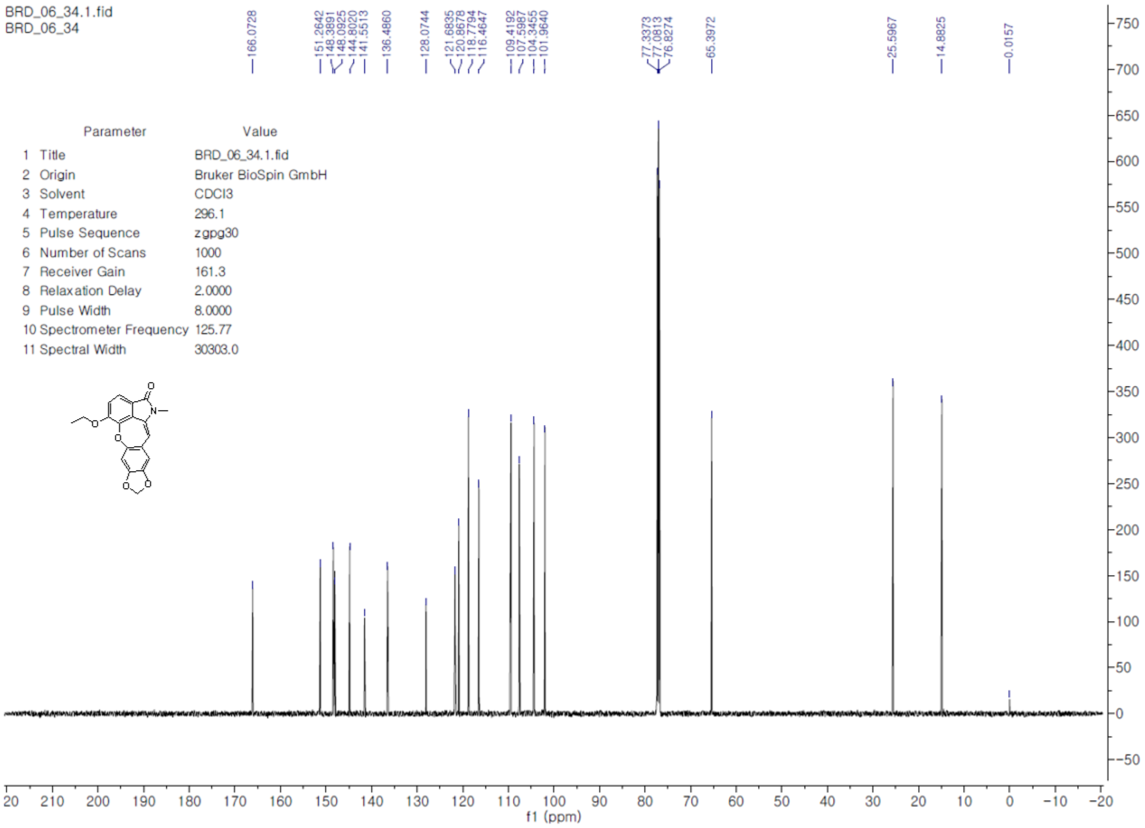

Compound 8ca

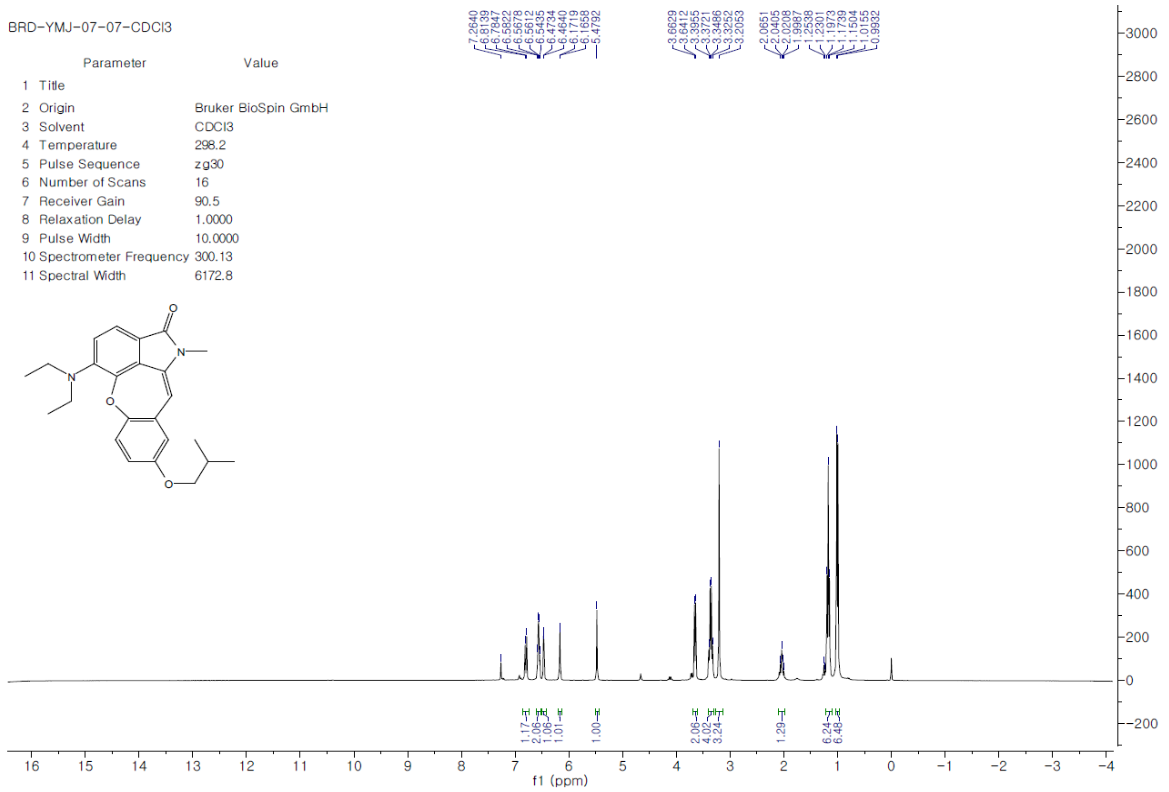

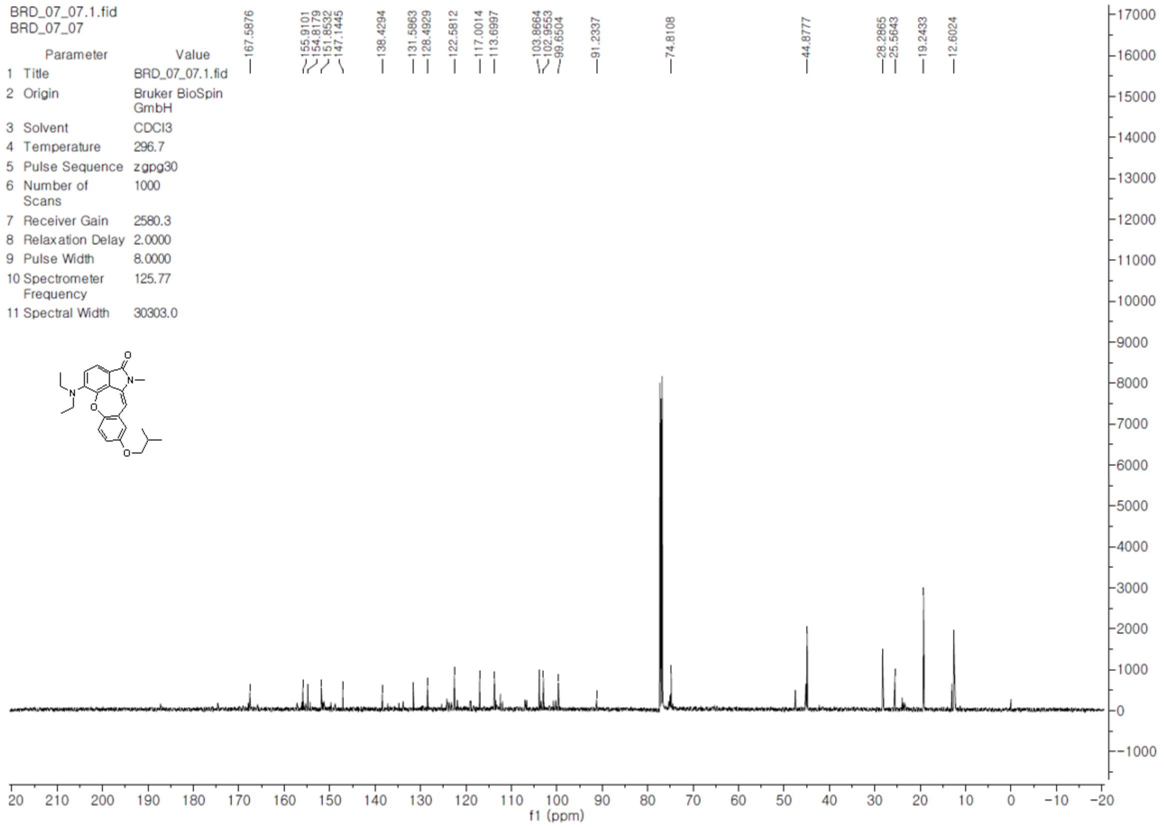

Compound 8cb

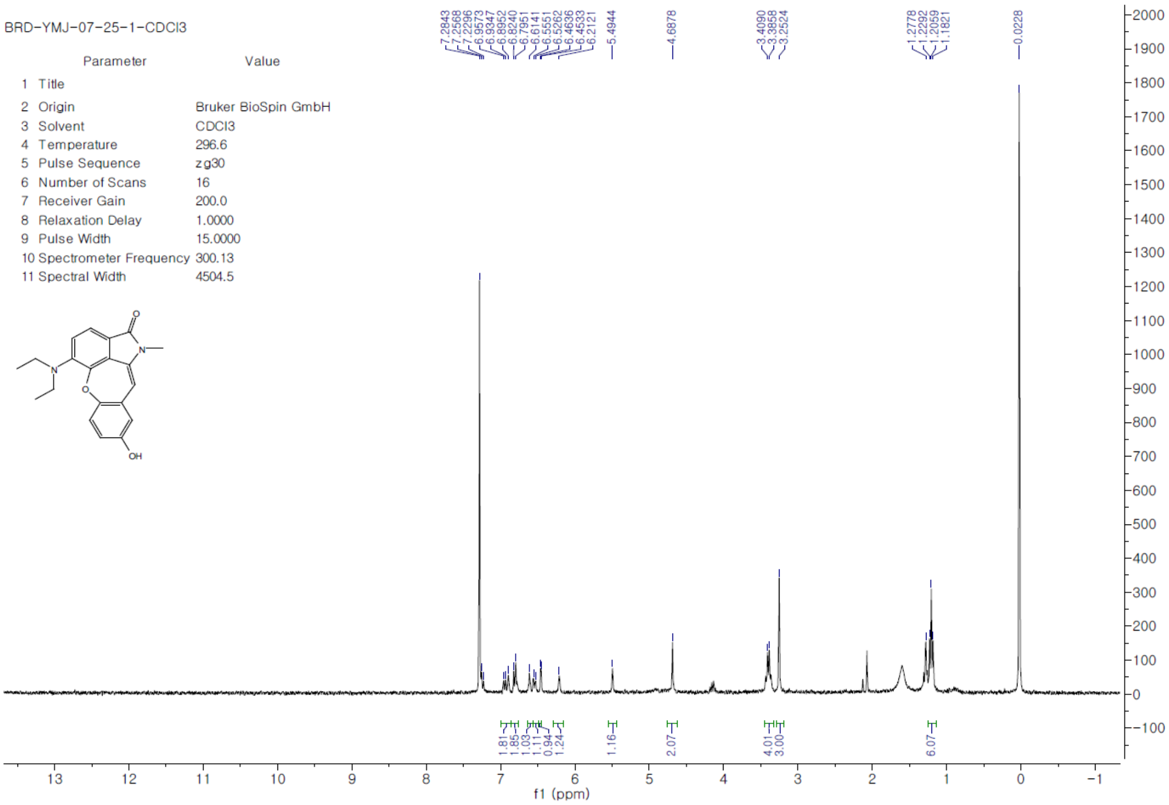

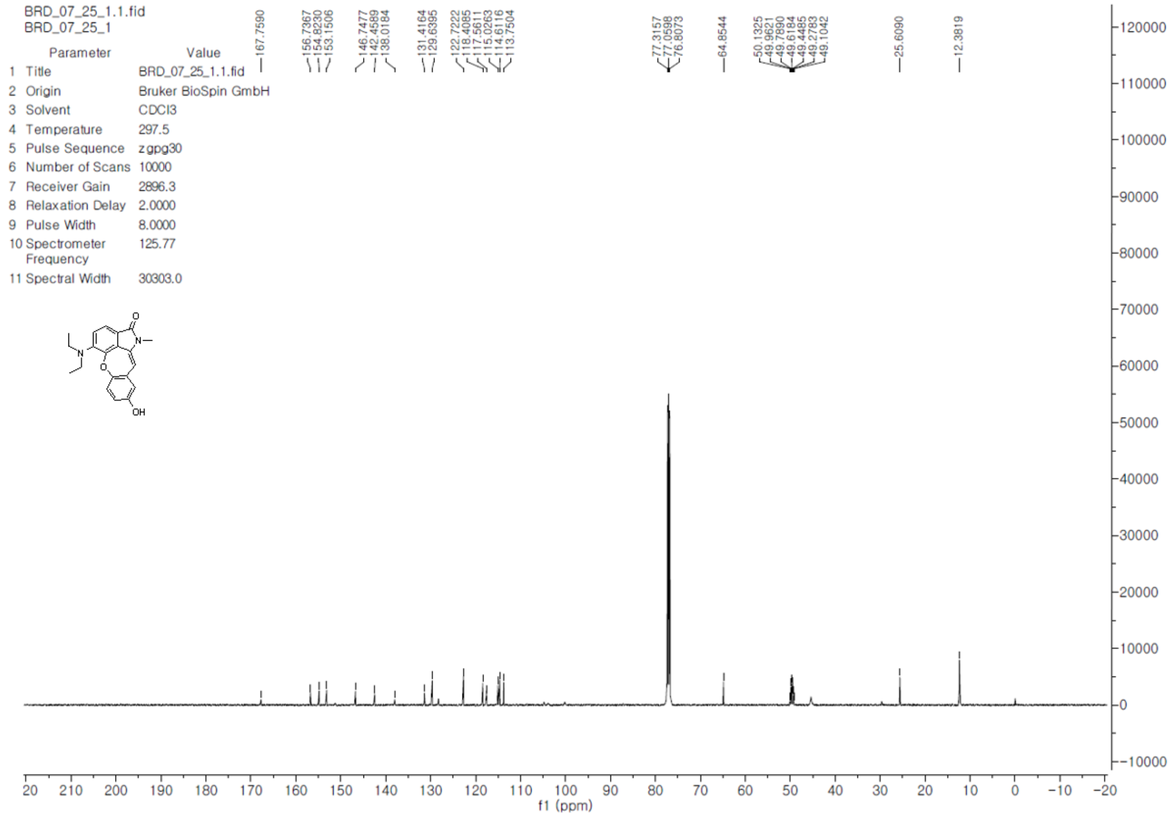

Compound 8cc

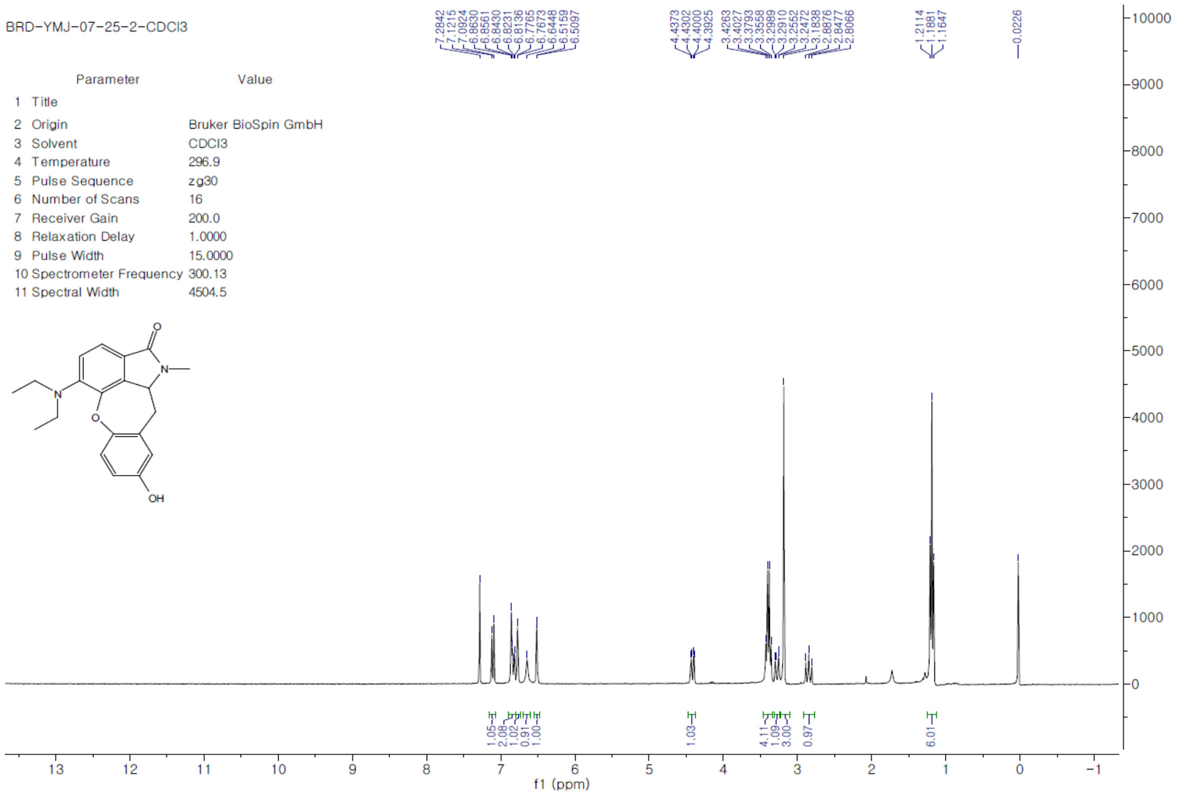

	Parameter	Value
1	Title	
2	Origin	Bruker BioSpin GmbH
3	Solvent	CDCl3
4	Temperature	296.9
5	Pulse Sequence	zg30
6	Number of Scans	16
7	Receiver Gain	200.0
8	Relaxation Delay	1.0000
9	Pulse Width	15.0000
10	Spectrometer Frequency	300.13
11	Spectral Width	4504.5

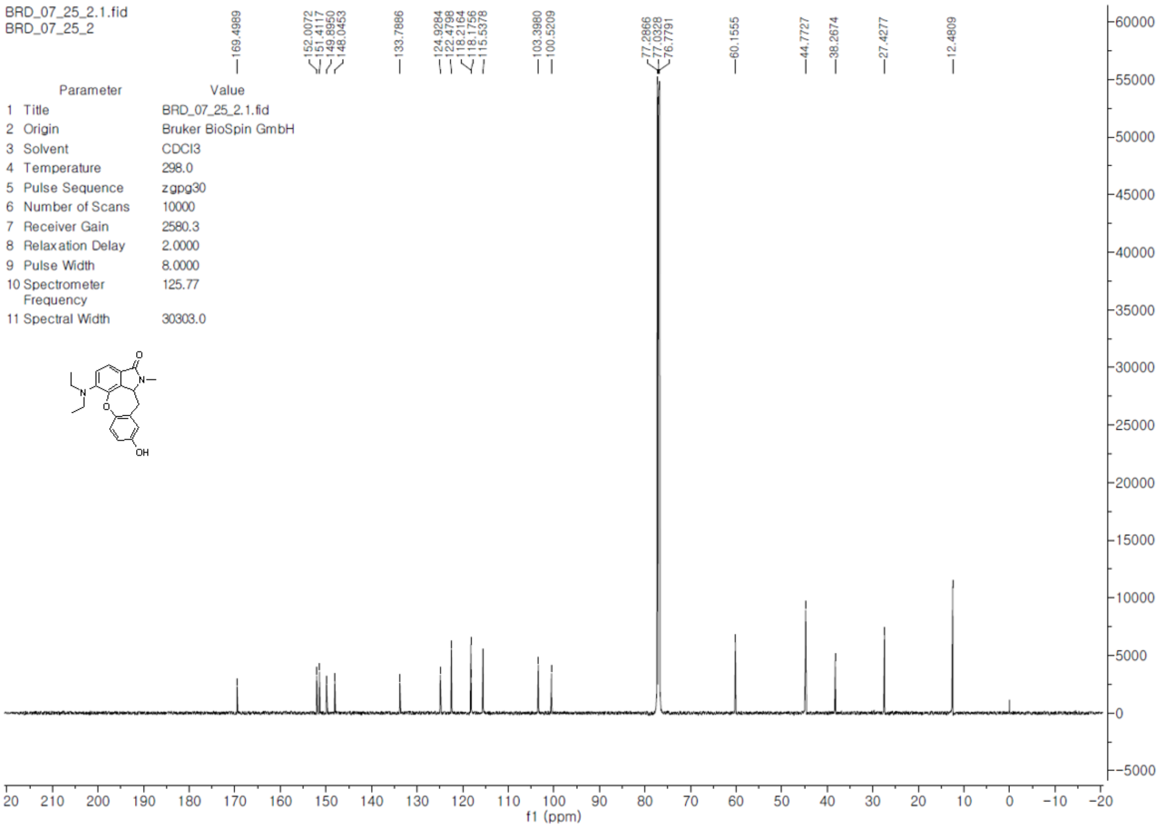

	Parameter	Value
1	Title	BRD_07_25_2.1.fid
2	Origin	Bruker BioSpin GmbH
3	Solvent	CDCl3
4	Temperature	298.0
5	Pulse Sequence	zgpg30
6	Number of Scans	10000
7	Receiver Gain	2580.3
8	Relaxation Delay	2.0000
9	Pulse Width	8.0000
10	Spectrometer Frequency	125.77
11	Spectral Width	30303.0

Compound 8cd

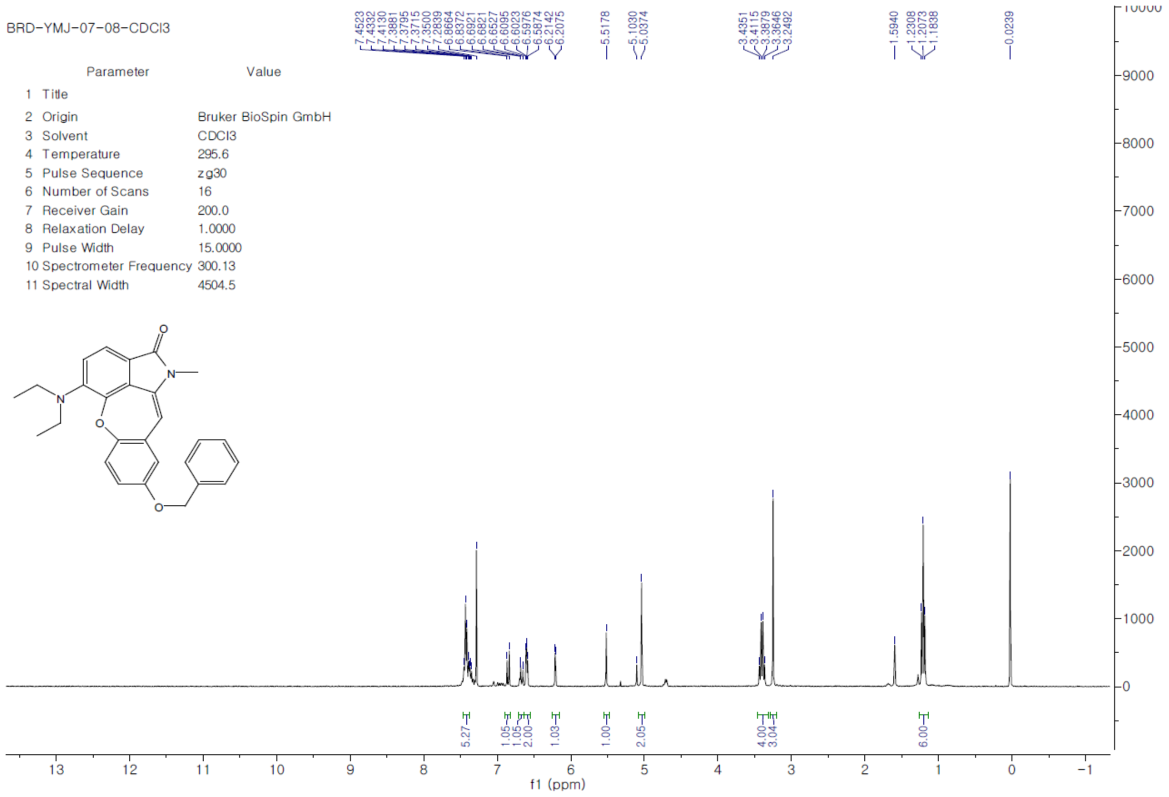

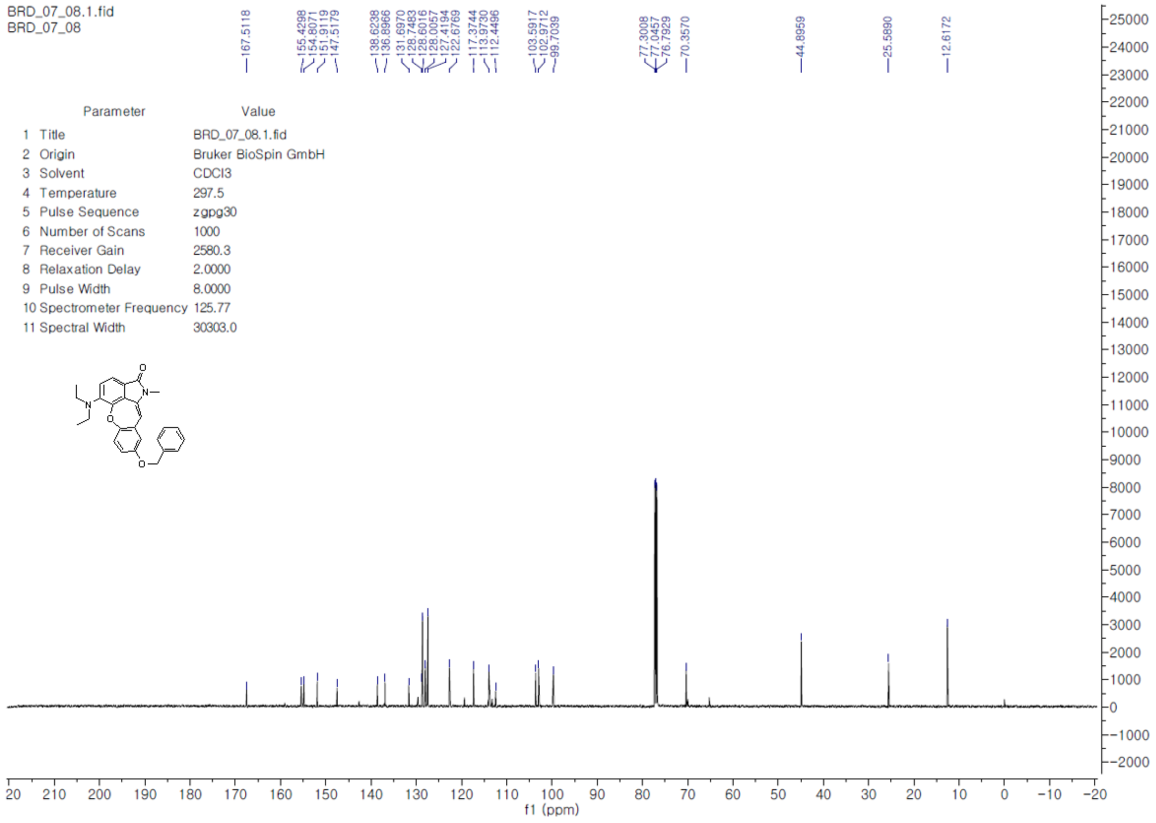

Compound 8ce

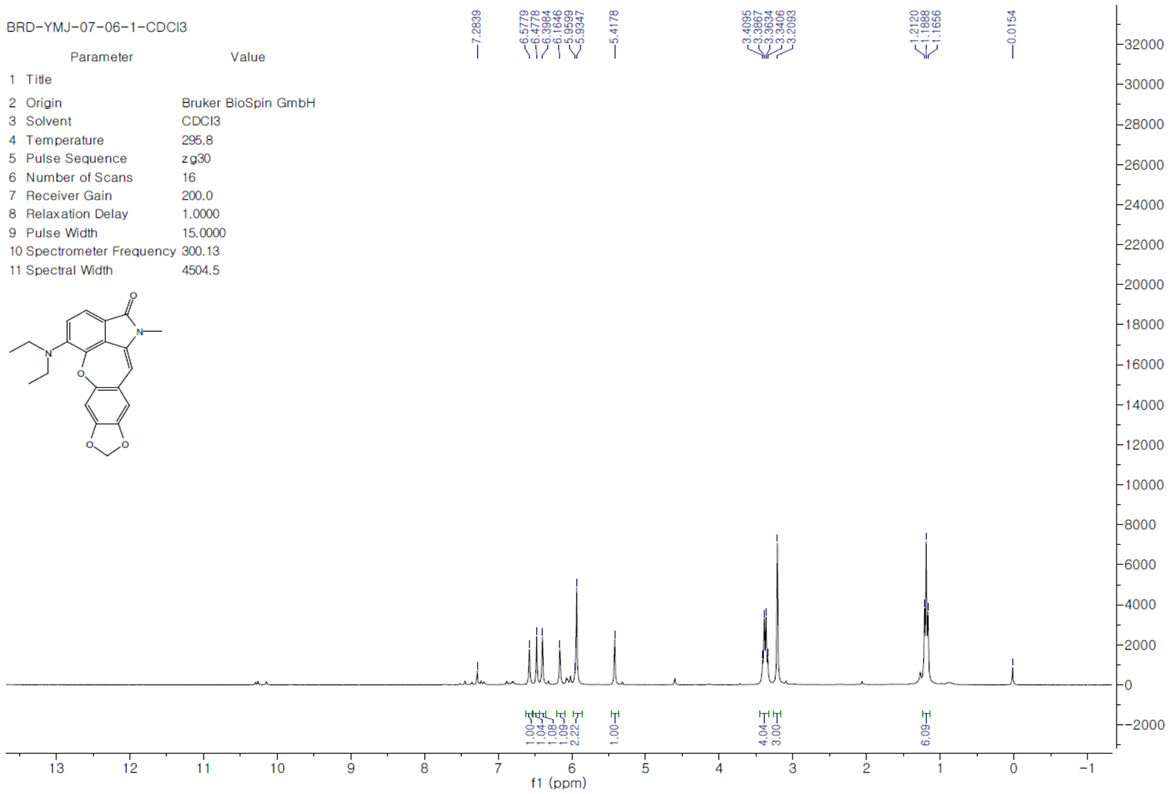

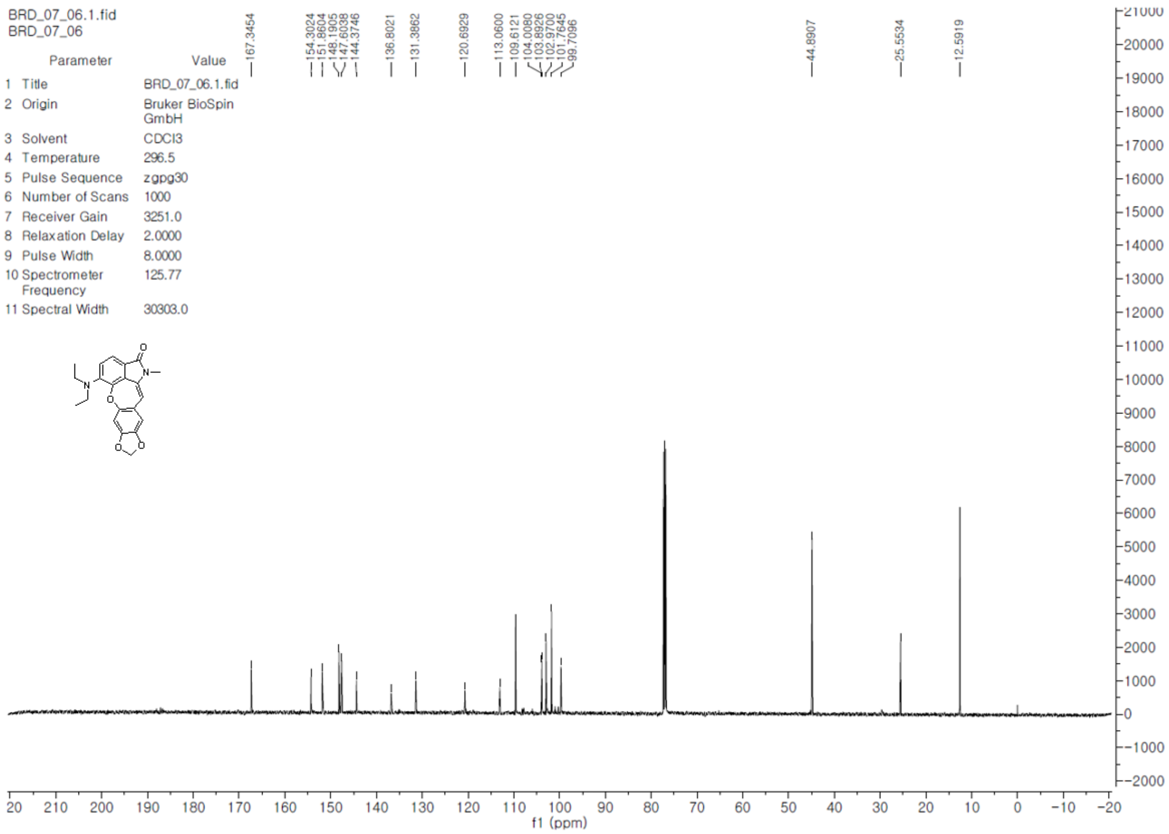

Compound 8da

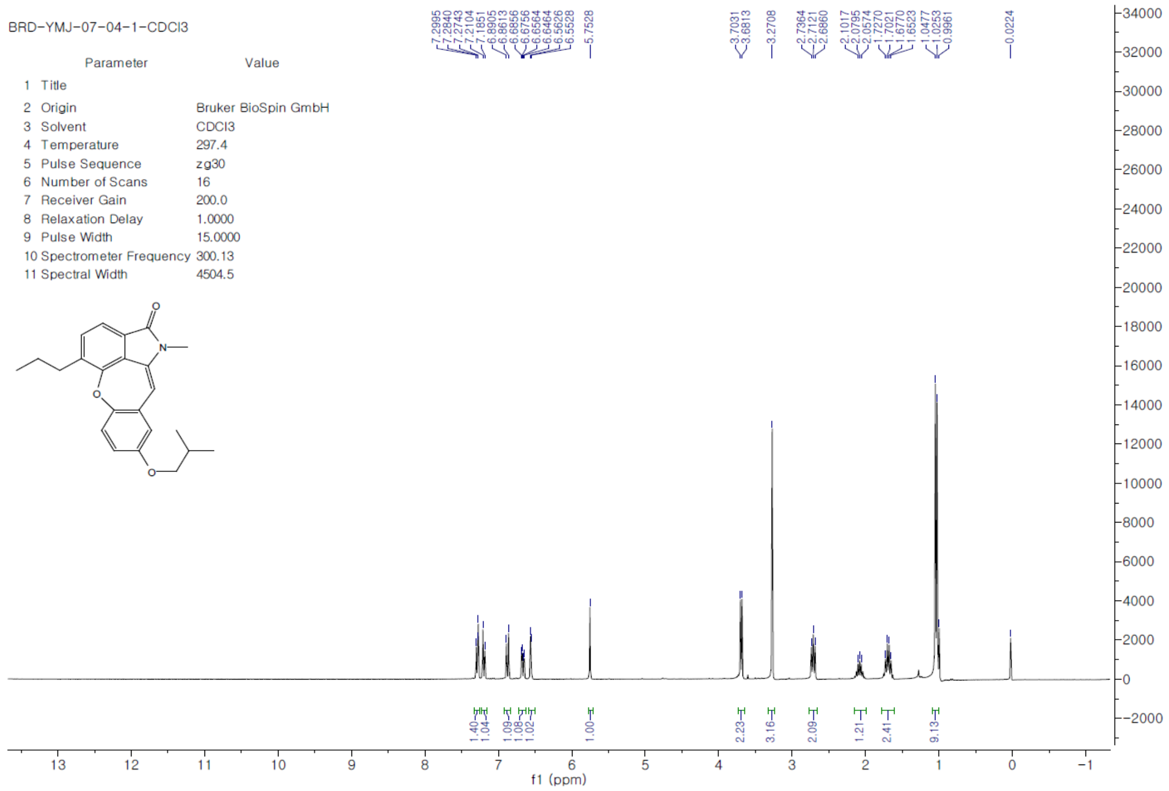

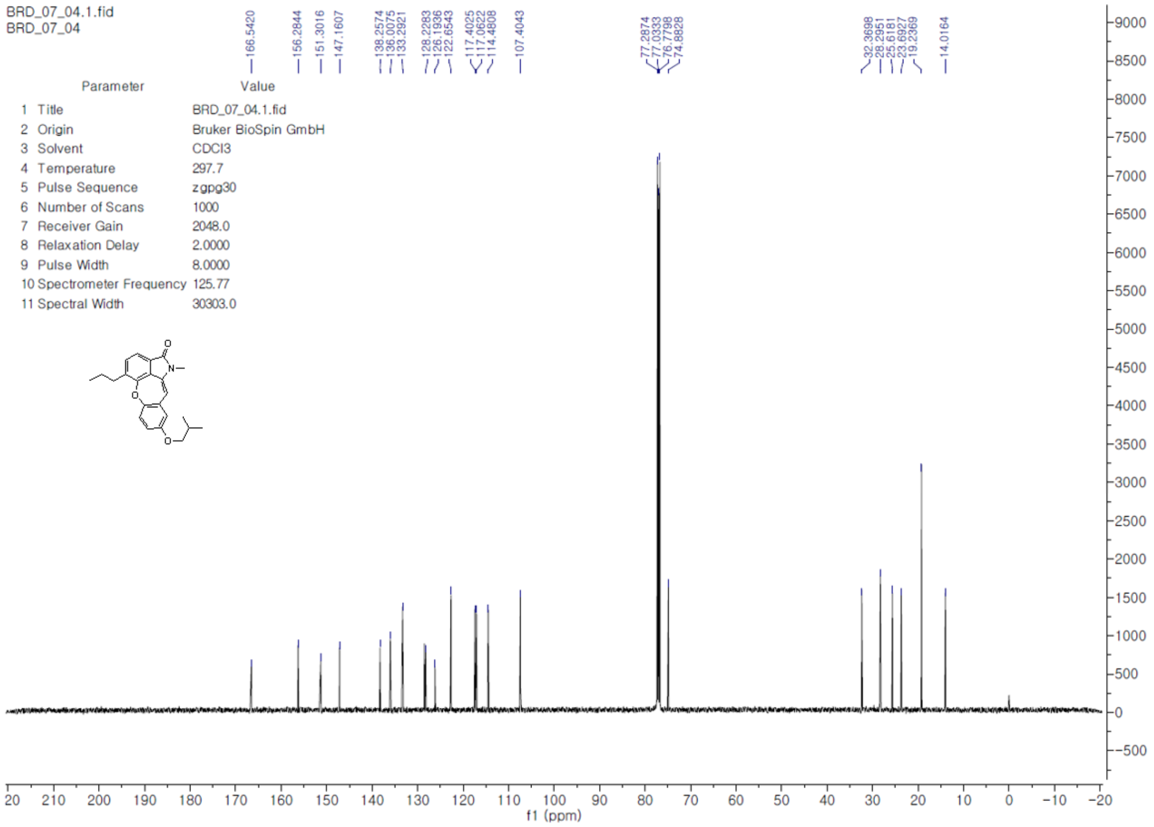

Compound 8db

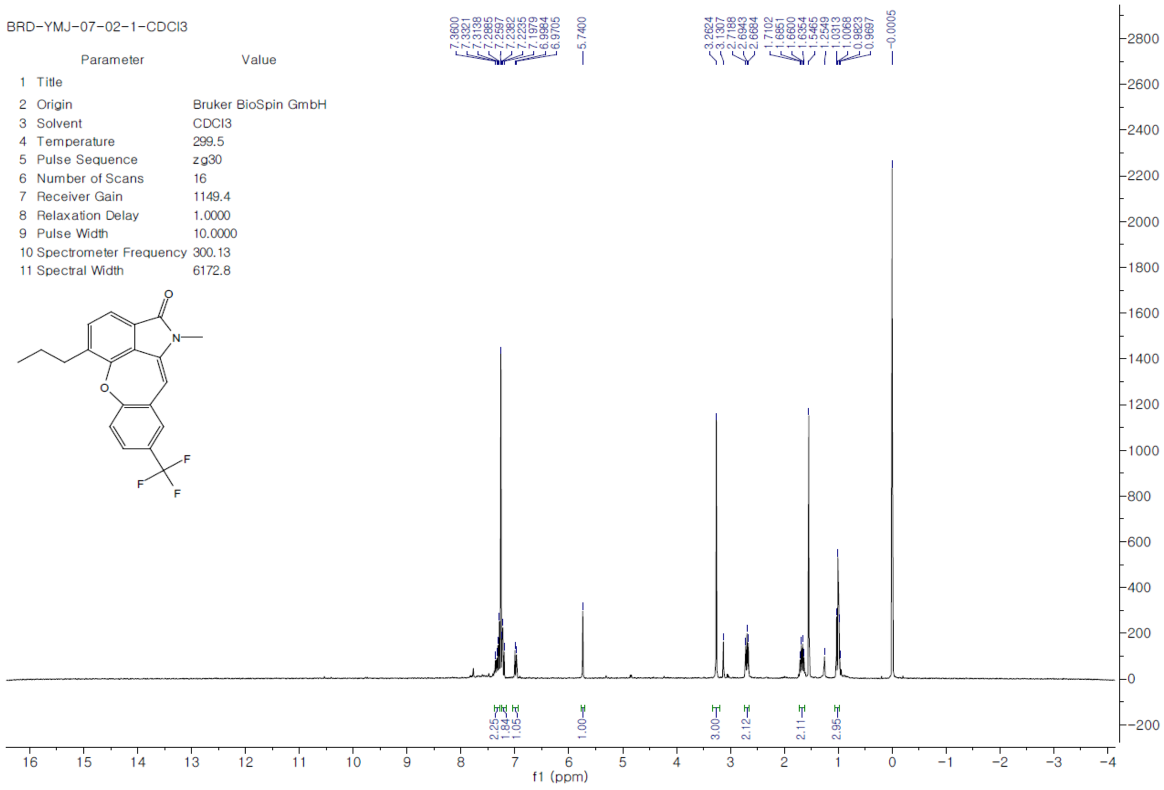

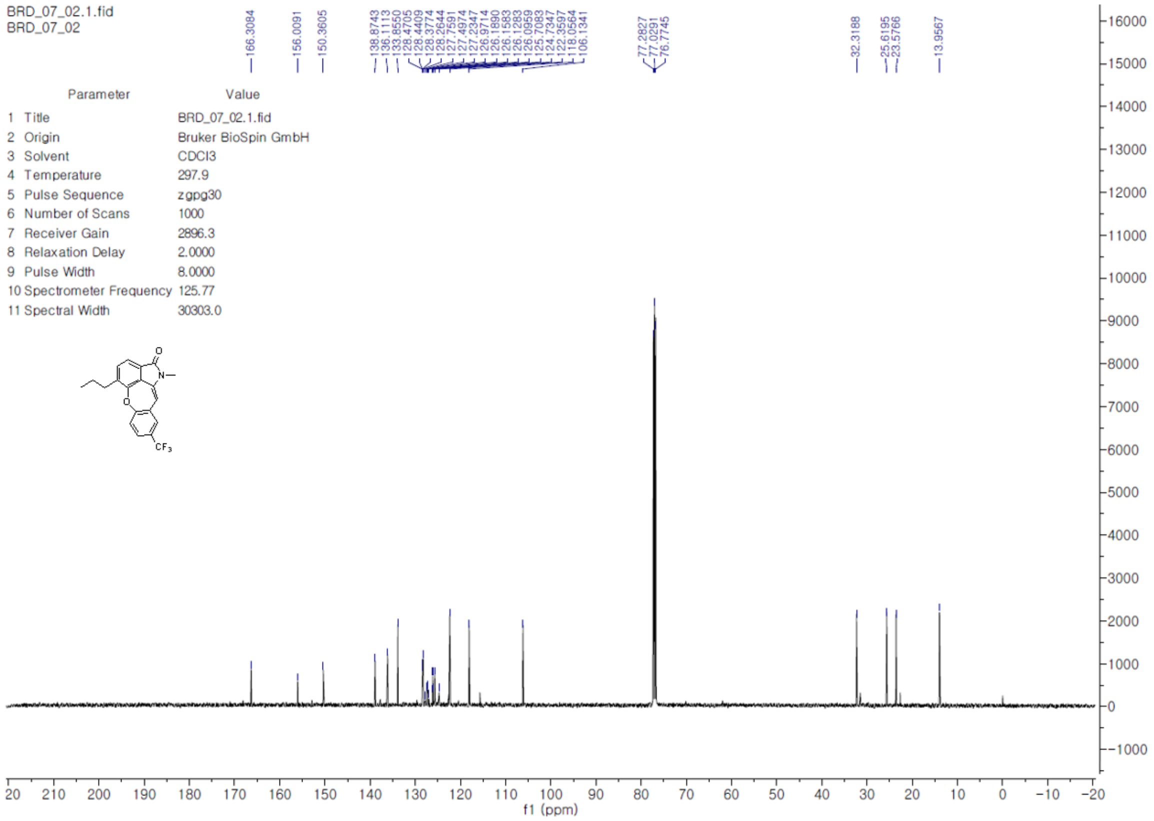

Compound 8dc

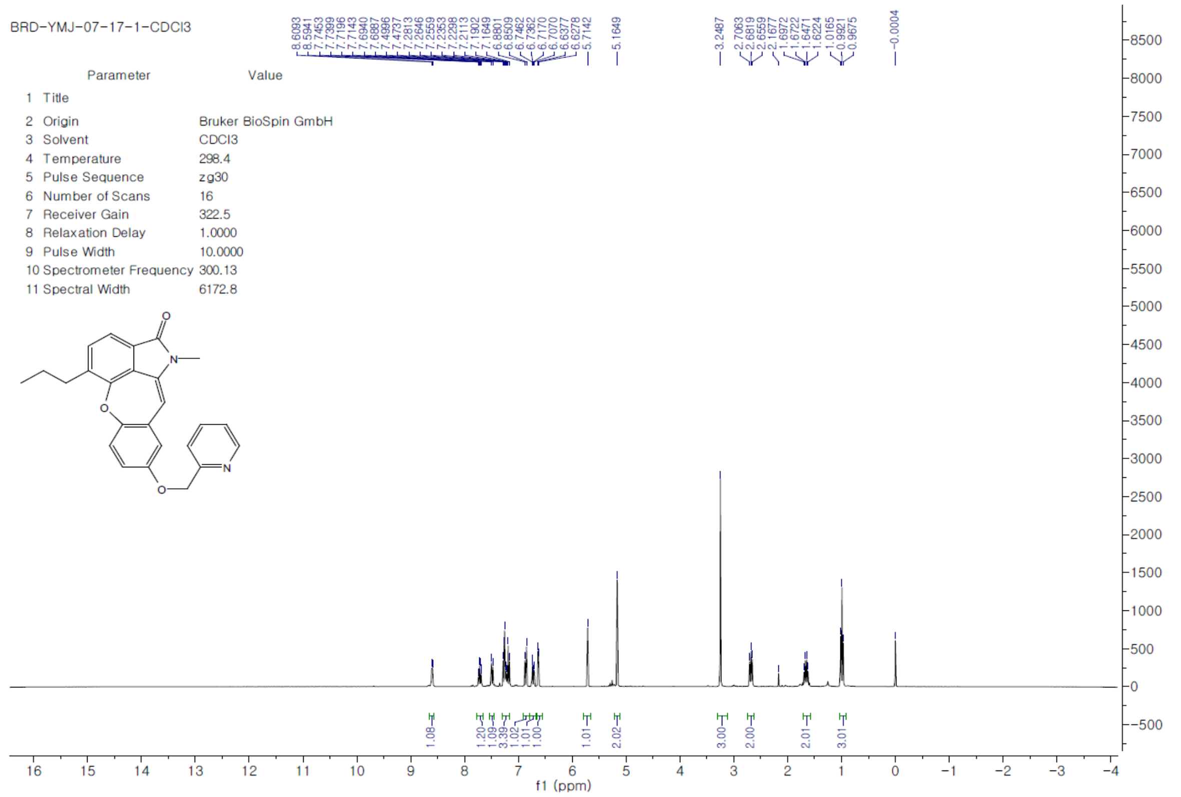

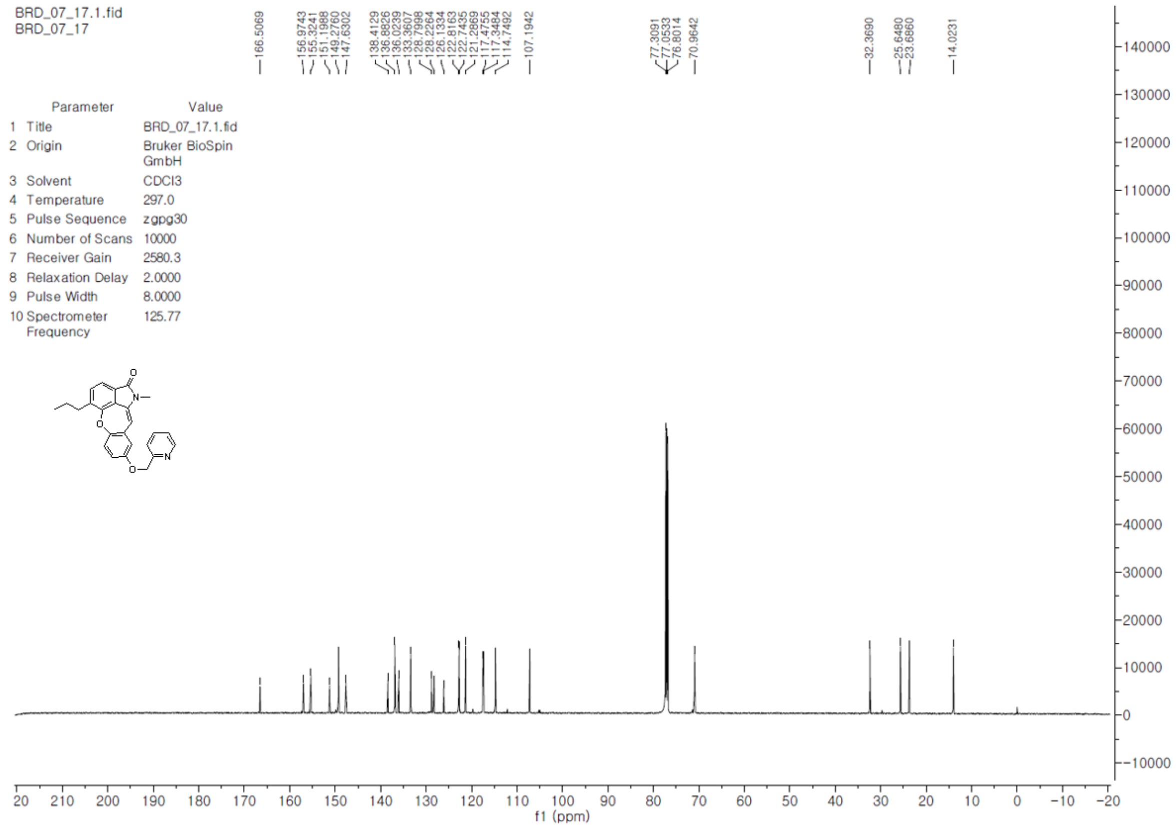

Compound 8dd

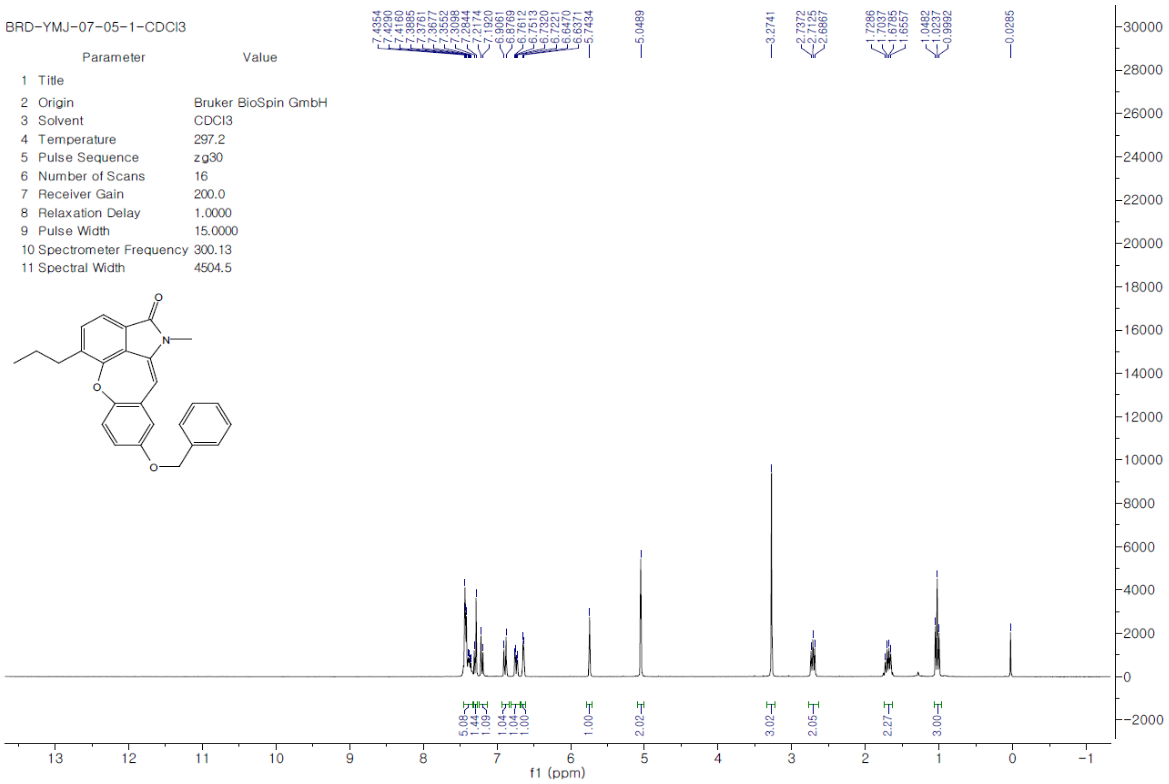

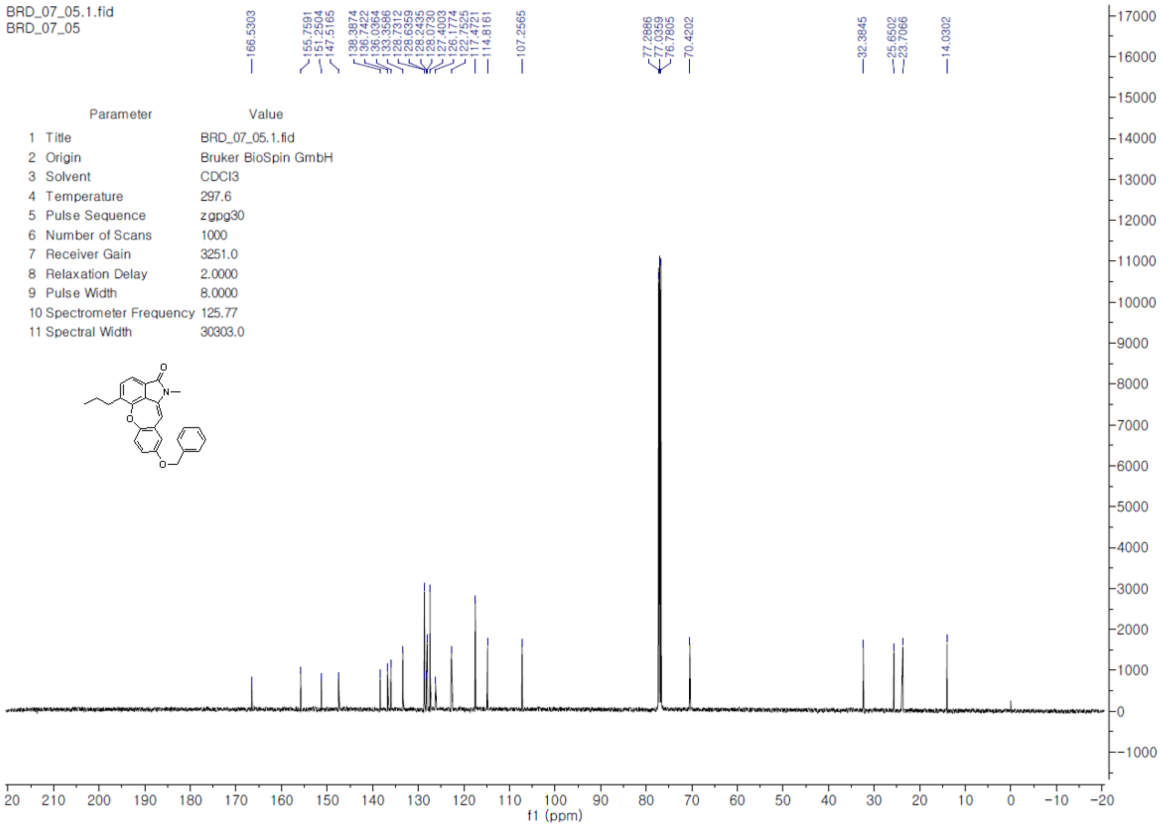

Compound 8de

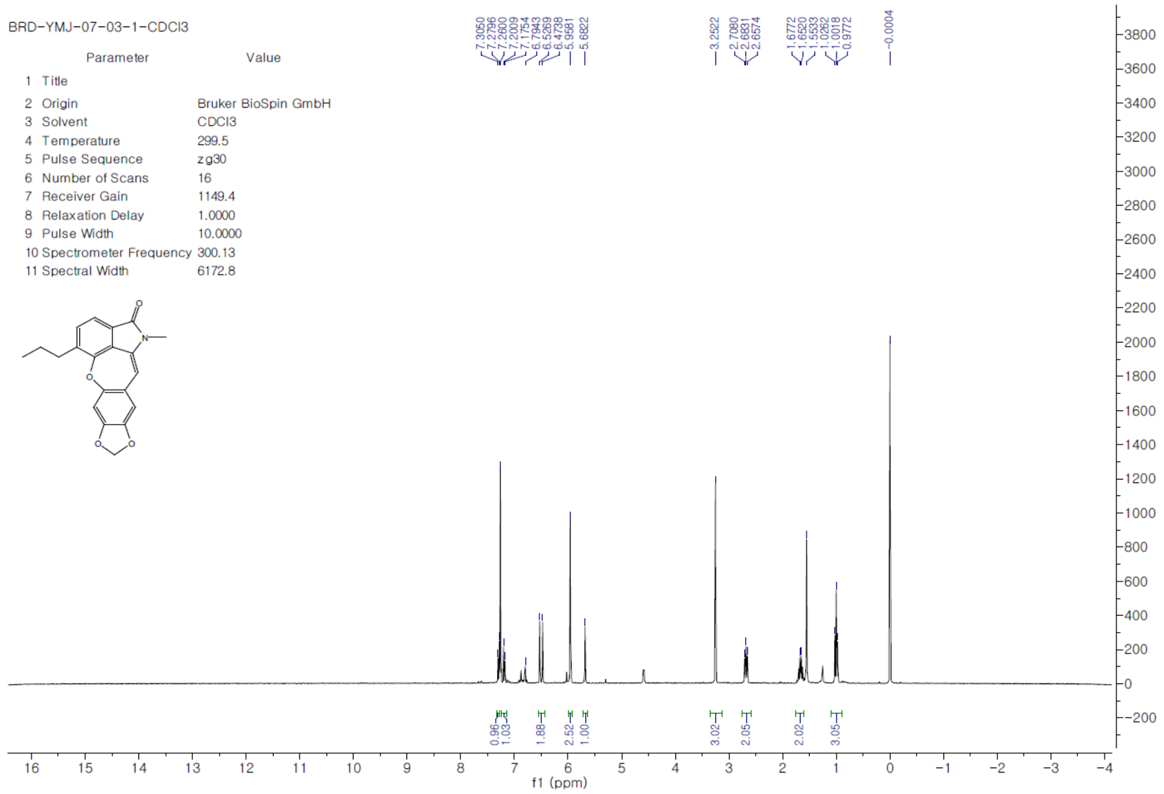

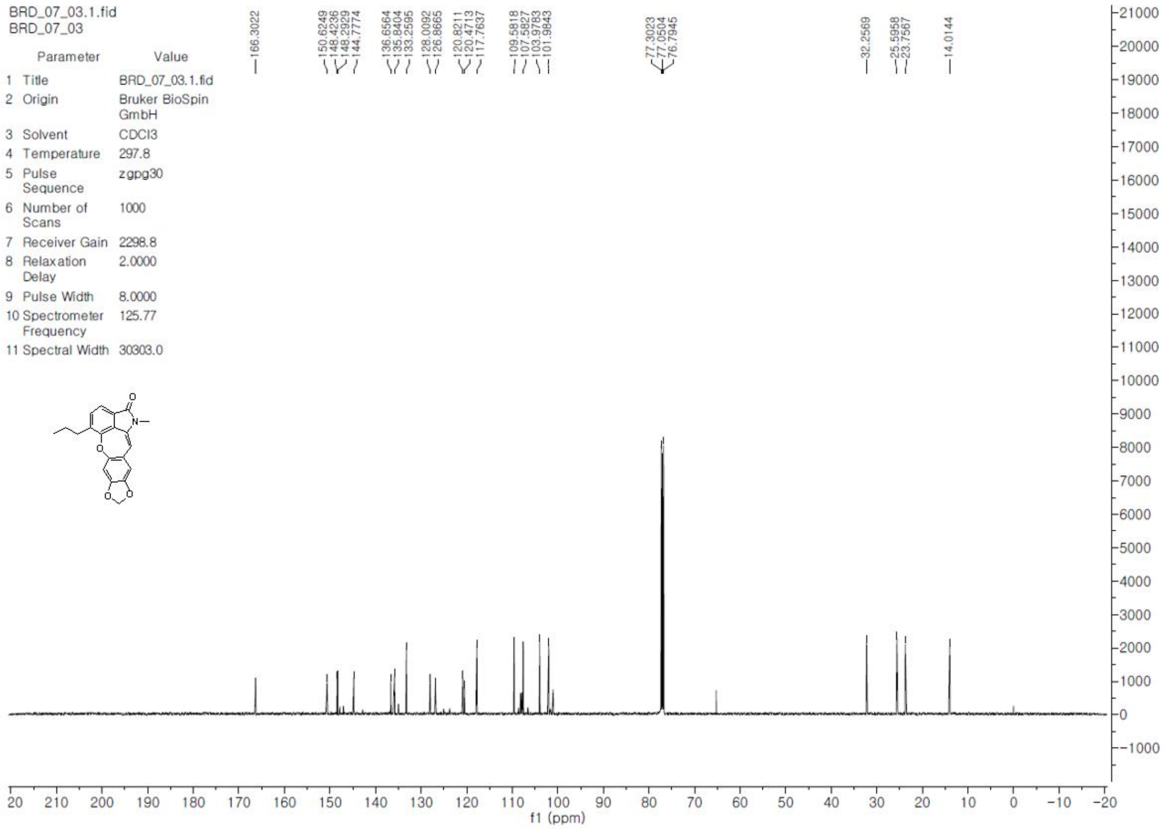

Compound 8df

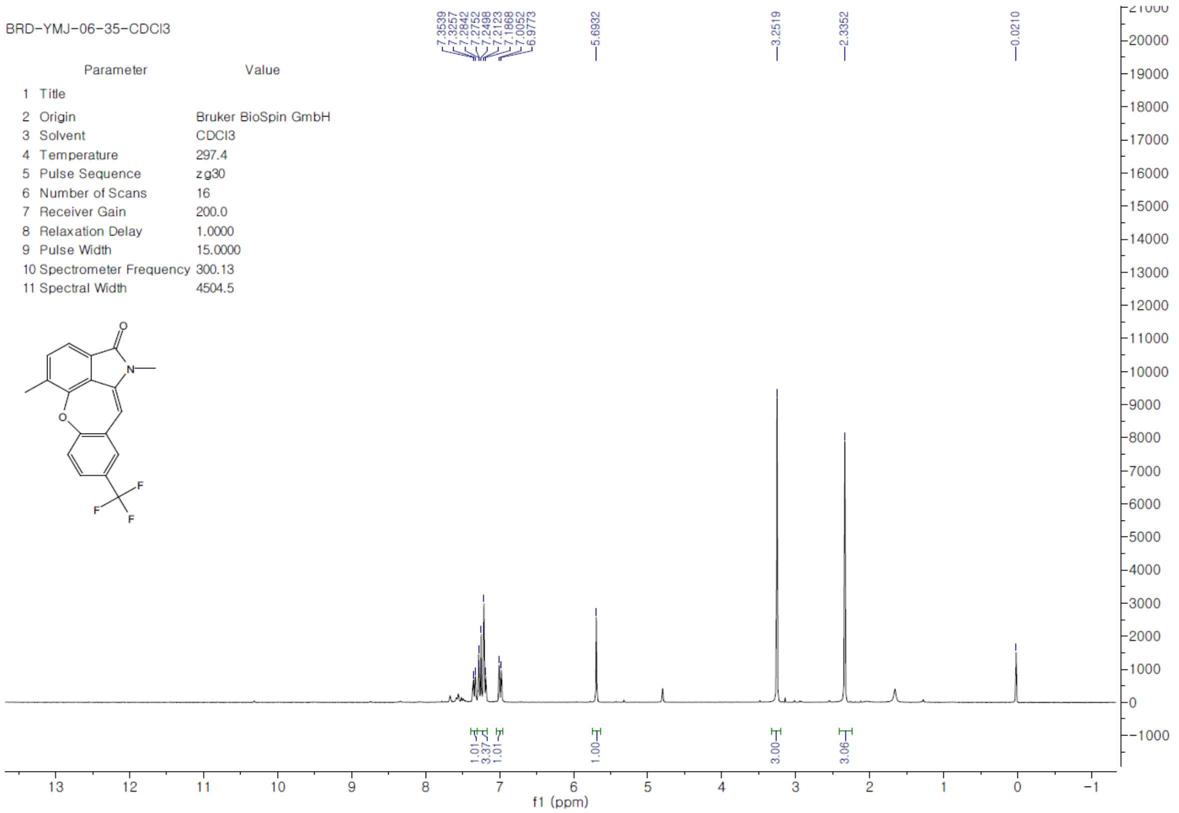

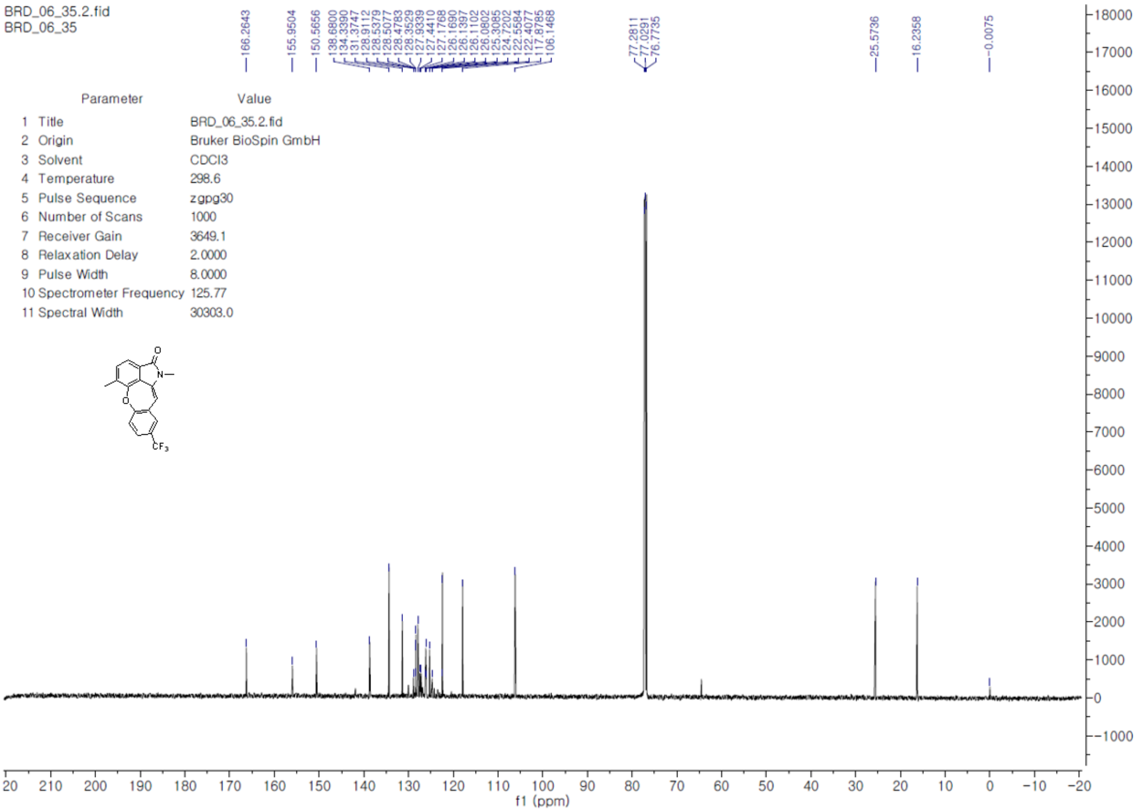

Supplement: Supplementary file 1 [file molecules-26-01686-s001.zip › molecules-1133239-supplementary.pdf]
